# Binding of BRACO19 to a Telomeric G-Quadruplex DNA Probed by All-Atom Molecular Dynamics Simulations with Explicit Solvent

**DOI:** 10.3390/molecules24061010

**Published:** 2019-03-13

**Authors:** Babitha Machireddy, Holli-Joi Sullivan, Chun Wu

**Affiliations:** College of Science and Mathematics, Rowan University, Glassboro, NJ 08028, USA; babitha.machireddy@gmail.com (B.M.); hollisullivan27@gmail.com (H.-J.S.)

**Keywords:** BRACO19, human telomeric G-quadruplexes, molecular dynamics binding simulations

## Abstract

Although BRACO19 is a potent G-quadruplex binder, its potential for clinical usage is hindered by its low selectivity towards DNA G-quadruplex over duplex. High-resolution structures of BRACO19 in complex with neither single-stranded telomeric DNA G-quadruplexes nor B-DNA duplex are available. In this study, the binding pathway of BRACO19 was probed by 27.5 µs molecular dynamics binding simulations with a free ligand (BRACO19) to a DNA duplex and three different topological folds of the human telomeric DNA G-quadruplex (parallel, anti-parallel and hybrid). The most stable binding modes were identified as end stacking and groove binding for the DNA G-quadruplexes and duplex, respectively. Among the three G-quadruplex topologies, the MM-GBSA binding energy analysis suggested that BRACO19′s binding to the parallel scaffold was most energetically favorable. The two lines of conflicting evidence plus our binding energy data suggest conformation-selection mechanism: the relative population shift of three scaffolds upon BRACO19 binding (i.e., an increase of population of parallel scaffold, a decrease of populations of antiparallel and/or hybrid scaffold). This hypothesis appears to be consistent with the fact that BRACO19 was specifically designed based on the structural requirements of the parallel scaffold and has since proven effective against a variety of cancer cell lines as well as toward a number of scaffolds. In addition, this binding mode is only slightly more favorable than BRACO19s binding to the duplex, explaining the low binding selectivity of BRACO19 to G-quadruplexes over duplex DNA. Our detailed analysis suggests that BRACO19′s groove binding mode may not be stable enough to maintain a prolonged binding event and that the groove binding mode may function as an intermediate state preceding a more energetically favorable end stacking pose; base flipping played an important role in enhancing binding interactions, an integral feature of an induced fit binding mechanism.

## 1. Introduction

The first therapeutically important G-quadruplex sequence was located in the single stranded 3′ overhang of human telomeric DNA [1,2], and contains numerous repeats of d(TTAGGG)_n_ sequences capped by Shelterin complexes [3,4,5,6]. The Shelterin complexes provide protection against nuclease attacks, chromosomal end-to-end fusion and gene erosion at cell divisions [7]. After each cell replication the telomere truncates by 50–200 base pairs, when the telomere is exhausted and the Hayflick limit is reached, cell senescence and apoptosis are triggered [8,9]. In cancer cells, a reverse transcriptase telomerase adds nucleotides to the telomere immortalizing the cells [10,11]. Telomerase is found to be overexpressed in 80–85% of tumor cells underscoring why telomerase inhibition is a logical therapeutic approach in cancer treatment. Despite the potential of this approach challenges include: (i) a time delay in which the telomere length needs to be established for the ultimate apoptosis trigger [8,12,13] and (ii) that studies suggest an alternate mechanism for telomerase maintenance might be activated upon telomerase inhibition [14,15,16]. However, it has been reported that the telomere cannot be hybridized by telomerase when the single stranded 3′ overhang folds into a G-quadruplex [17], instead the telomeric G-quadruplex is perceived as DNA damage and stimulates cell level apoptosis [2,18]. Accentuating how a ligand that stabilizes the telomeric G-quadruplex can be an efficacious anti-cancer therapy.

Extensive research has been performed that show G-quadruplexes are highly polymorphic and their topological fold depends on factors such as nucleic acid sequence, ions and the presence of small molecules [19,20,21,22,23]. Though studied for decades, the most biologically relevant topological fold of human telomeric G-quadruplex remains an elusive and controversial debate. In 1993, Wang and Patel published the first solution structure of the human telomeric sequence d[AG3(T2AG3)3] in Na^+^ containing solution which adopts anti-parallel topology (PDB ID: 143D); formally referred to as 3(-lwd+ln) [24]. In 2002, Parkinson and coworkers published a K^+^ containing crystal structure of the human telomeric DNA in a parallel topological fold, referred to as 3(-p-p-p) in Table 1 using the nomenclature recently described by Dvorkin et al. [25] (PDB ID: 1KF1) [26]. The parallel crystal structure published by Parkinson and coworkers was different from the preceding studies which reported the DNA in an anti-parallel topological fold in Na^+^ containing solution [24,27,28]. In the years following, experiments providing evidence for both topological folds continued to publish. The parallel topological fold was suggested the most biologically relevant form in K^+^ containing crystal because the polymorphism of the G-quadruplex structure was lost in 40% PEG or 50% ethanol solutions, ie. dehydrated solutions [29]. Heddi and Phan studied the human telomeric sequence under crowded conditions with NMR, using the same dehydrating crowding agents used in X-ray crystallography, and found that the parallel conformation predominated [30]. In contrast, solution studies using NMR and 125I-radioprobing were also published providing evidence for the anti-parallel topology in both K^+^ and Na^+^ containing solution, several of which reported the parallel and anti-parallel topologies coexist under both ionic conditions [31,32,33,34]. Not long after Parkinson and coworker’s parallel crystal structure was published solution studies began to identify additional topological folds for the human telomeric DNA [35,36,37,38,39,40]. Yang and coworkers [41] showed the same sequence with two additional naturally occurring adenine residues at the 5′ and 3′ termini in K+ solution folds into a hybrid scaffold in 2007 (PDB ID: 2HY9), formally referred to as 3(-p-lw-ln) in Table 1. The skepticism regarding the predominating topology in physiological conditions has led to studies suggesting that rather than the parallel topology, both the anti-parallel [34,35,42] and hybrid [37,41] forms are most physiologically relevant.

The contradicting results being published gave rise to discussion regarding the experimental accuracy of using dehydrating crowding agents like PEG to report the structure of the highly flexible and dynamic DNA G-quadruplex. In 2005, Li and coworkers published work that directly refuted the biological relevance of the parallel stranded crystal structure of the human telomeric DNA G-quadruplex reported by Parkinson and coworkers delineating that by using certain dehydrating solvents, like PEG, crowding conditions are not being mimicked but rather conditions of extreme water depletion that is misrepresentative of physiological conditions [35]. Using acetyl nitrile, a non-dehydrating crowding agent, Miller et al. proposed the structure of the human telomeric sequence was not identical to the structure published in crystalline state, supporting the role of hydration in the stability and conformation of this human telomeric sequence. Using Ficoll and *Xenopus laevis* egg extract compared to PEG, Hansel and coworkers (doi:10.1093/nar/gkr174) suggested the parallel scaffold is not the preferred topology under physiological conditions, but rather the parallel, anti-parallel, and hybrid topologies co-exist under native conditions. Stating that in Ficoll or cellular extracts, the more predominate conformations in the co-existing equilibrium mixture are likely the anti-parallel and/or the hybrid topologies. Analyzing the studies overall, evidence suggests that this sequence forms multiple intramolecular G-quadruplex scaffolds in K+ solution and the intramolecular parallel G-quadruplex observed in the K+-containing crystal appears unlikely to be the major form in K+-containing solution [32,33,43,44,45,46,47]. Given the broad range of evidence to support each of the three scaffolds and without evidence to rule out the predominance of any of the reported scaffolds, one of each the parallel, anti-parallel, and hybrid topological folds were modeled in this study.

**Figure 1:**
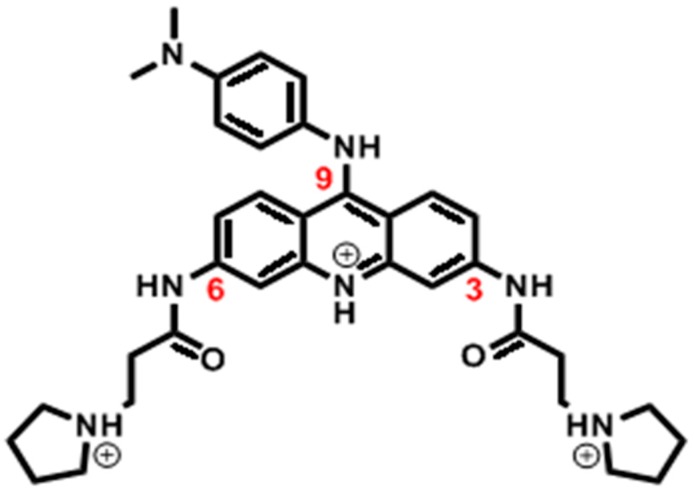
Chemical structure of BRACO19.

Based on structural requirements of the parallel-stranded telomeric G-quadruplex binding site, BRACO19, a tri-substituted acridine (Figure 1), was rationally designed with computer modelling [48,49] and has since been one of the most widely studied G-quadruplex binders. BRACO19 has been reported to inhibit telomerase which causes telomere shortening [50]; its experimental in-vivo activity against a variety of cell lines is reported (Table 2). Furthermore, BRACO19 have been shown effective in anti-viral, and anti-parasitic treatments. BRACO19 also demonstrates broad anti-viral activity by stabilizing the G-quadruplexes found in pro-viral genomes [51] such as the viruses: HIV-1, HSV-1, EBV, HHV-6, and HBV [52]. BRACO19 also showed in vitro anti-parasitic activity in bloodstream forms of *T. brucei brucei*, against promastigotes of *L. major*, against *P. falciparum* [53], as well as against a human non-tumoral lung cell line (MRC-5) [54]. A major factor that has prevented BRACO19 from clinical usage is a low selectivity towards G-quadruplex over duplex DNA (*K*_quad/*K*_dup = 40-fold, *K*: ligand binding constant) [49], which has the potential to cause reverse effects. To achieve higher selectivity (e.g., 10^5^-fold), better understanding of the binding nature of BRACO19 with DNA G-quadruplex and duplex DNA is required.

Despite the high interest of BRACO19 in complex with biologically relevant single stranded intramolecular DNA G-quadruplex formed by one chain (e.g., d(AGGGTTAGGGTTAGGGT TAGGG)), there is no high-resolution structure of BRACO19 binding to the antiparallel and the hybrid topology. The only high-resolution structure available is a bimolecular parallel G-quadruplex in complex with BRACO19 (PDB ID: 3CE5), where the intermolecular G-quadruplex is formed from two 12 residue chains (i.e., d(TAGGGTTAGGGT)) [71]. Because bimolecular (12mer) or intramolecular (22mer) adopt the same parallel topology, suggested by Parkinson et al. [26] and later confirmed by Phan et al. [30] in both Na+ or K+ in solution under crowded conditions, this crystal structure provides the following invaluable interaction insights: BRACO19 interacts asymmetrically with the guanine bases of the intermolecular G-quadruplex through π–π interactions and the nitrogen atom of the acridine ring aligns with the K^+^ cations within the ion pore. Nonetheless the additional 5′ and 3′ residues at the two ends could introduce artifacts when comparing the BRACO19 binding modes on the biologically relevant unimolecular parallel scaffold formed the single stranded telomeric sequence. To remove the artifacts, we used another crystal structure (PDB ID: 1KF1) containing the apo form of the parallel intramolecular telomeric G-quadruplex in our BRACO19 binding studies. Furthermore, because the most biologically relevant form may not be the parallel form, the binding of BRACO19 to the antiparallel and the hybrid form are required to better understand its biology relevant binding mode leading to its anti-cancer properties [19,35].

Computationally, molecular docking and molecular dynamics (MD) stability simulations have been widely used in studying G-quadruplexes in complex with BRACO19 and other ligands. Haider and Neidle studied the stability of human telomeric DNA G-quadruplex repeats using molecular models of dimer and tetramer telomeric G-quadruplex repeats followed by MD simulations [72]. Collie et al. used MD simulation to study the stability of BRACO19 and three naphthalene diimide ligands in complex with a parallel stranded telomeric DNA G-quadruplex (PDB ID: 1KF1), using the solved binding pose from the crystal structure of BRACO19 (PDB ID: 3CE5) as a starting point [73]. Xu et al. [74] used molecular docking to study a propeller-shaped trinuclear Pt^II^ complex with the anti-parallel G4 (PDB ID: 143D) and showed binding to the major groove closest to the 5′ end. Moore et al. conducted MD simulations to investigate the structure-activity relationships of BRACO19 analogs and a modeled 22mer parallel G-quadruplex [75]. Hou et al. revealed hydrogen bonds to be the major contributor of stability for the G-quadruplex and ligand-quadruplex complex by conducting stability simulations on G-quadruplex-ligand complexes involving BRACO19 and 5 other ligands [76]. Dhamodharan et al. suggested end-stacking as the favored binding mode after docking bis-quinolinium and bis-pyridinium derivatives of 1,8-naphthyridine onto anti-parallel G-quadruplex and further conducting MD simulations [77]. Jain et al. reported that both end-stacking and groove-binding were favored after docking dimeric 1,3-phenylene-bis(piperazinyl benzimidazole)s to a 22mer parallel G-quadruplex followed by MD simulations [78]. Ungvarsky et al. characterized the binding poses of a novel set of BRACO19 derivatives to the human telomeric parallel G-quadruplex by successfully employing docking and MD simulations [79]. Also, Diveshkumar et al. conducted a docking and MD simulation study on various G-quadruplexes (PDB IDs: 2L7V, 2O3M, 1KF1, 143D, and 2MB3) and identified indolyl, methylene-indanone scaffolds which demonstrate selectivity towards parallel promoter G-quadruplexes over telomeric DNA quadruplex or duplex DNA [80]. Nonetheless, these stability simulations do not provide detailed information on the binding pathway and low selectivity.

**Figure 2:**
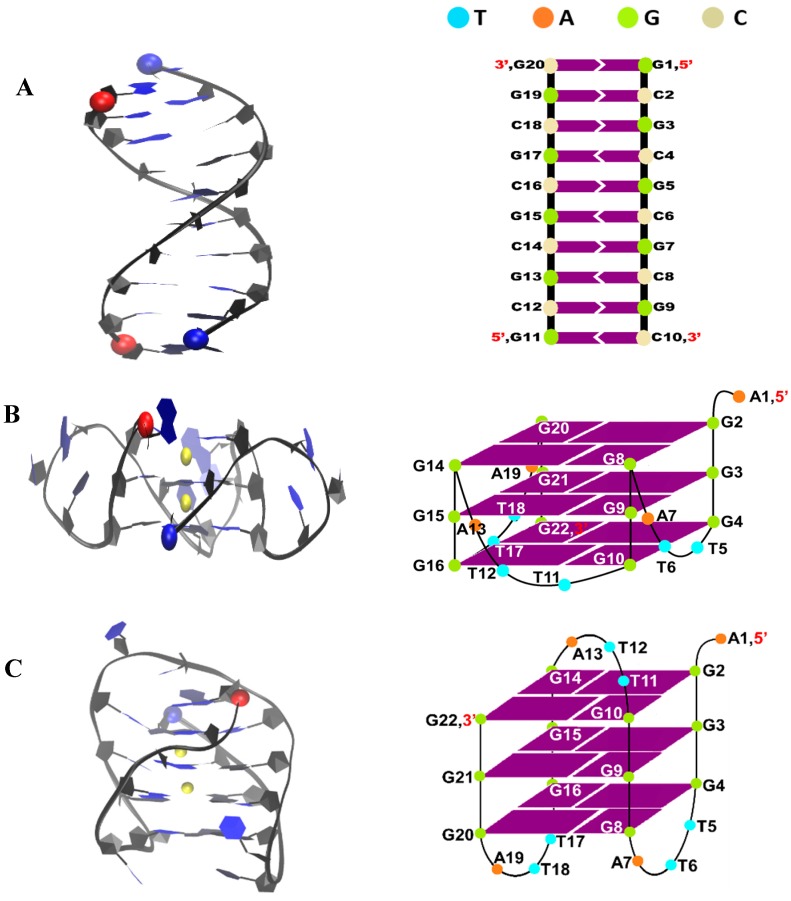

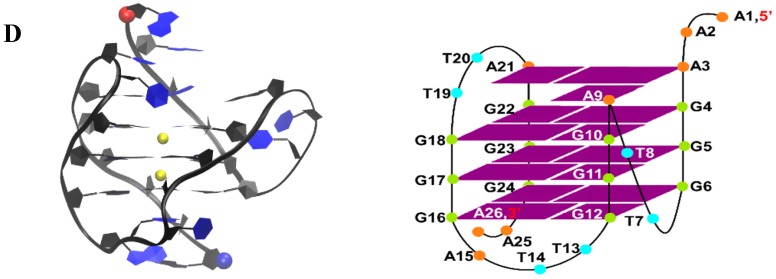
Structure of human telomeric duplex DNA (**A**), human telomeric parallel DNA G-quadruplex (PDB ID: 1KF1) 3(-p-p-p) (**B**), human telomeric anti-parallel DNA G-quadruplex (PDB ID: 143D) 3(-lwd+ln) (**C**), and human telomeric hybrid DNA quadruplex (PDB ID: 2HY9) 3(-p-lw-ln) (**D**). 5′ and 3′ of the DNA chain are indicated by a red and blue ball, respectively.

Deng et al. showed that using two statistical mechanics-based free energy simulation methods (potential of mean force and double-decoupling method) allows for binding affinity calculations at various binding sites in the c-MYC G-quadruplex which is in agreement with experimental findings [81]. The use of µs-scale simulations with the latest AMBER force fields have shown to provide a good evaluation of the loop conformation of the G-quadruplexes [58]. Using µs-scale simulations with the latest AMBER force fields in our previous work produced detailed and experimentally verified predictions [82,83,84,85,86]. In this work, by using free ligand MD binding simulations with AMBER OL15 DNA and GAFF2 ligand force fields [84] (Table 1), we were able to predict a binding mode of BRACO19 to the double stranded parallel telomeric G-quadruplex that is consistent with the crystal complex structure (PDB ID: 3CE5). Furthermore, the binding modes and the ligand binding pathways were characterized in detail. We extended our free ligand MD simulations to characterize the binding pathway of BRACO19 to the parallel, anti-parallel, hybrid DNA G-quadruplexes and duplex DNA (Figure 2). Major binding poses, (top binding, bottom binding and groove binding) were identified and detailed binding pathways were characterized. The dynamic and energetic properties of the three major binding modes were thoroughly studied. We suggest that the similar binding energy of the groove binding pose to the duplex and of the top stacking pose to the parallel G-quadruplexes may be responsible for the low selectivity (40-fold) of BRACO19. This study may provide insight into the further modification of BRACO19 and other G-quadruplex binders to enhance overall selectivity and efficacy.

## 2. Results

### 2.1. Multiple Drug Binding Modes Were Observed in Free Ligand Binding Simulations

**Figure 3:**
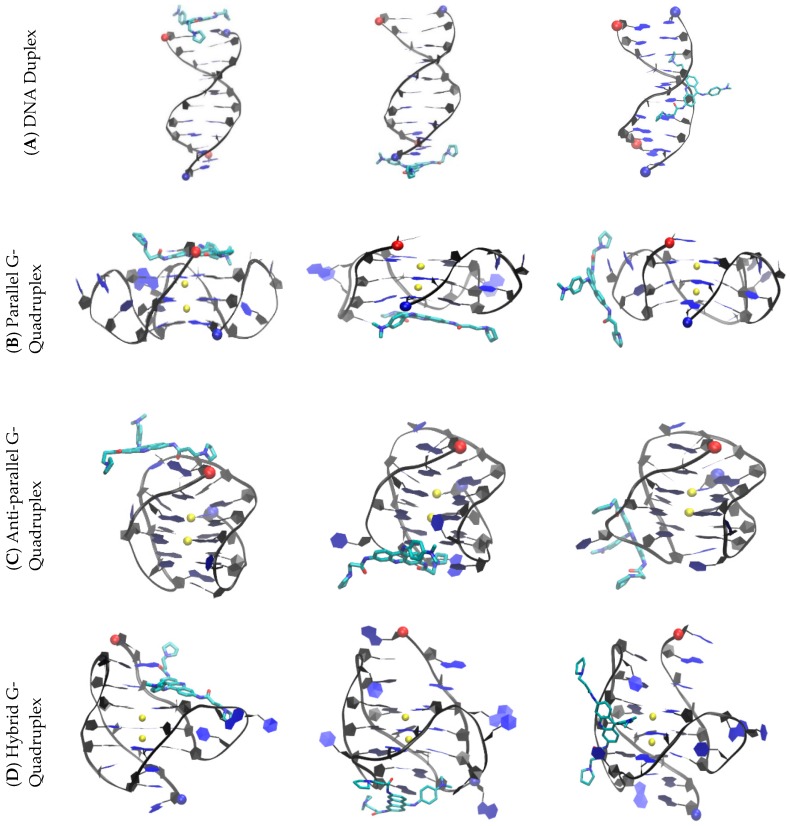
The major binding modes of BRACO19 in complex with the human telomeric DNA. (**A**) Duplex; (**B**) Parallel G-quadruplex; (**C**) Anti-parallel human telomeric G-quadruplex; (**D**) Hybrid human telomeric G-quadruplex. (**A**–**D**) Top binding (**left**), Bottom binding (**middle**) and groove binding (**right**); 5′ end and 3′ end are represented by the red and blue ball respectively.

Starting from an unbound state, we simulated ten 500 ns production runs for each system. The convergence of the binding simulations was confirmed (see the method section), a sampling plot was generated to trace the position of one atom of BRACO19 through the length of the entire simulation period for each system (Figure S31). The last snapshots from each simulated trajectory of the duplex-BRACO19 system is listed in Figure S10 and indicate the stability of the DNA structures where the base pairing was maintained. The last snapshots of all the simulated trajectories of the G-quadruplex-ligand systems are listed in Figures S11–S13 and indicate the stability of the G-quadruplex structures where the G-tetrads were maintained. The major binding modes of BRACO19 in complex with the parallel, anti-parallel and hybrid telomeric DNA is presented in Figure 3. Multiple binding sites were observed in the ten duplex DNA-BRACO19 trajectories. The clustering analysis described in the methods section was employed to categorize the stable complexes that were extracted from the trajectories into structural families. By setting a threshold of 1% population, 14 structural families of complexes were identified (Figure S14). These eight structural families were further merged into three binding modes: groove binding, top stacking and bottom stacking. Binding to the groove of the duplex accounted for 81% of the total population. Additionally, end stacking to the top of the duplex accounted for 4% and end stacking to the bottom of the duplex made up 2% of the total population (Figure S14). Three binding modes were observed in the ten parallel G-quadruplex DNA-BRACO19 trajectories. The clustering analysis was employed to categorize the stable complexes that were extracted from the trajectories into 11 structural families (Figure S15). The three binding modes observed were: top stacking, bottom stacking and groove binding. Top stacking to the parallel G-quadruplex DNA accounted for 28%, bottom stacking accounted for 41% and groove binding for 29% of the total population (Figure S15). Multiple binding sites were observed in the ten anti-parallel DNA G-quadruplex-BRACO19 trajectories. The clustering analysis was employed to categorize the stable complexes that were extracted from these trajectories into 9 structural families (Figure S16). Three binding modes were observed: top, bottom and groove binding. Bottom binding to the anti-parallel G-quadruplex DNA accounted for 46%, top binding for 25% and groove binding for 40% of the total population (Figure S16). Multiple binding sites were observed in the ten hybrid G-quadruplex DNA-BRACO19 trajectories. The same clustering analysis was employed to categorize the stable complexes that were extracted from these trajectories into 11 structural families (Figure S17). Three binding modes were observed: top, groove and bottom binding. Groove binding to the hybrid G-quadruplex DNA accounted for 43%, top binding for 33%, and bottom binding for 20% of the total population (Figure S17). Two dimensional interaction diagrams of BRACO19 in complex with each DNA system, in each major binding pose, is available in the supporting document (Figure S18).

### 2.2. VDW Interaction Contributes Most to the Total Binding Energy and in Ranking the Binding Poses for Each DNA-Ligand System

MM-GBSA binding energy calculations were carried out, as depicted in methods section, to examine the relative binding affinities of the major binding modes of BRACO19 with respect to the DNA and summarized in Table S1. The most favorable binding energy for the duplex-BRACO19 complex was the groove binding mode (−61.7 ± 8.0 kcal/mol), followed by the bottom stacking mode (−34.6 ± 5.7 kcal/mol) and the top stacking mode (−33.7 ± 5.3 kcal/mol). VDW packing, responsible for the VDW energy contribution, was the primary contributor to the binding energy of the three modes. BRACO19 bound to parallel G-quadruplex DNA in three binding pose where top stacking (−62.3 ± 4.5 kcal/mol) was the most energetically favorable, closely followed by the bottom binding mode (−61.8 ± 1.5 kcal/mol), and groove binding (−37.6 ± 7.2 kcal/mol) was the least stable of the three. BRACO19 bound to the anti-parallel G-quadruplex DNA in three binding poses where bottom binding (−53.9 ± 5.8 kcal/mol) was the most stable of the three, groove binding exhibiting a binding energy of −43.1 ± 7.2 kcal/mol and top binding had the lowest binding energy (−42.8 ± 4.1kcal/mol).

BRACO19 bound to the hybrid G-quadruplex DNA in three binding poses as well. Top binding (−40.5 ± 5.4 kcal/mol) was the most stable of the three, followed by groove binding (−35.7 ± 5.1 kcal/mol) and bottom binding (−29.0 ± 12.9 kcal/mol).

### 2.3. BRACO19 Binds to the Groove of the Duplex DNA, without Inducing Appreciable DNA Structure Fluctuation

**Figure 4:**
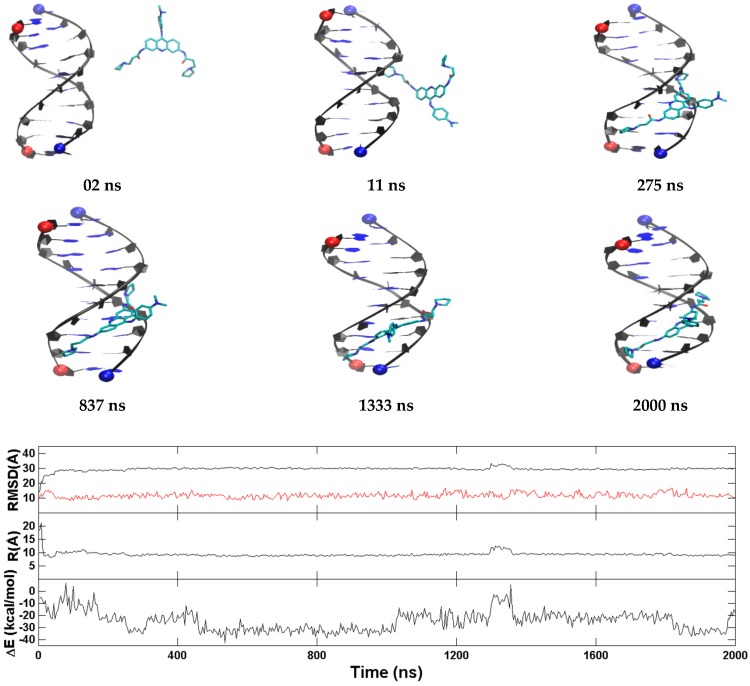
A representative groove binding trajectory of the duplex DNA. (**Top**) Representative structures with time annotation. 5′ and 3′ are indicated by a red and blue ball, respectively. (**Bottom**) receptor (red) and ligand (black) RMSD relative to the original crystal pose, center-to-center distance and MM-GBSA binding energy (ΔG) (cf. methods section for definition).

The representative trajectories for the three major binding modes of BRACO19 to the human telomeric duplex DNA are characterized in Figure 4 and Figure S19. In all ten binding trajectories, the DNA showed low structural fluctuation with a RMSD of 2.4 Å (Figure S2) where the hydrogen bonds between the base pairs were maintained. In the representative trajectory of BRACO19 binding to the groove of the human telomeric duplex DNA in Figure 4, an initial interaction was observed as early as 3 ns and the final binding pose was achieved at ~14 ns which was maintained throughout the remainder of the trajectory. The limited fluctuation in the five order parameters explained the limited structural dynamics. The other representative trajectory of BRACO19 groove binding (Figure S19) also exhibited rapid binding and limited dynamics.

### 2.4. Groove Binding of BRACO19 may be an Intermediate State for the top Stacking Mode of the Parallel G-Quadruplex

**Figure 5:**
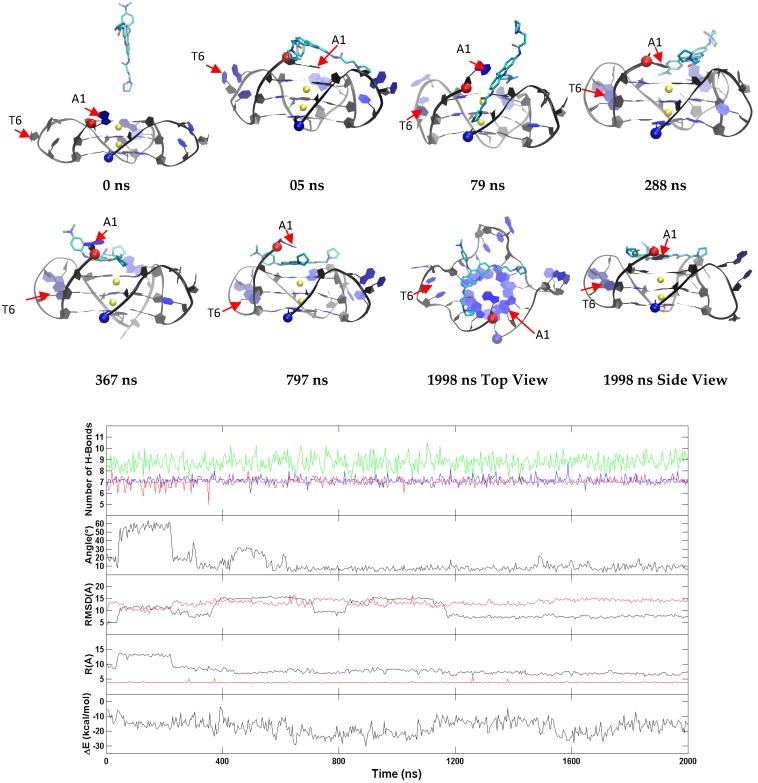
A representative top stacking trajectory of the parallel G-quadruplex. (**Top**) Representative structures with time annotation. 5′ and 3′ are indicated by a red and blue ball, respectively. K^+^ ions are represented in yellow. (**Bottom**) An order parameter plot depicting number of hydrogen bonds present in first G4 (green), second G4 (red) and third G4 (blue) tetrads of the DNA structure (Figure 2), the drug-base dihedral angle, receptor (red) and ligand (black) RMSD relative to the original crystal pose, center-to-center distance (R/black) and K^+^-K^+^ distance (R/red) and MM-GBSA binding energy (ΔG) (cf. methods section for definition).

The representative trajectories for the two major binding modes of BRACO19 to the parallel human telomeric G-quadruplex DNA are characterized in Figure 5 and Figure S20 (the top stacking mode) and Figure S21 (the bottom stacking mode). In all ten binding trajectories, the DNA showed low structural fluctuation with RMSD of 2.4 Å (Figure S4) and the hydrogen bonds in the three G-tetrads were maintained. In the representative trajectory of BRACO19 binding to the top of the human telomeric parallel G-quadruplex DNA in Figure 5, an initial interaction was observed as early as 2 ns. BRACO19 underwent several top pose adjustments until ~750 ns when the final binding pose was achieved and maintained throughout the remainder of the trajectory. Despite the repositions of BRACO19 in the early portion of the simulation, there were limited fluctuations in the order parameters. The other representative trajectory of the top stacking mode of BRACO19 binding to parallel G-quadruplex (Figure S20) also exhibited quick binding and limited dynamics, with the early interaction to the complex at 4 ns and attaining the stable top binding pose at 25 ns where it maintained the top staking pose with minor repositions until 1391 ns where it remained for the length of the trajectory. The representative trajectory for the bottom binding pose (Figure S21) achieved the final binding pose within 10 ns and displayed high stability as indicated by the limited fluctuations in the order parameter plot. The binding energy for top/bottom stacking fluctuated between −60 and −75 kcal/mol while groove stacking varied between −35 and −45 kcal/mol after attaining a stable binding pose.

**Figure 6:**
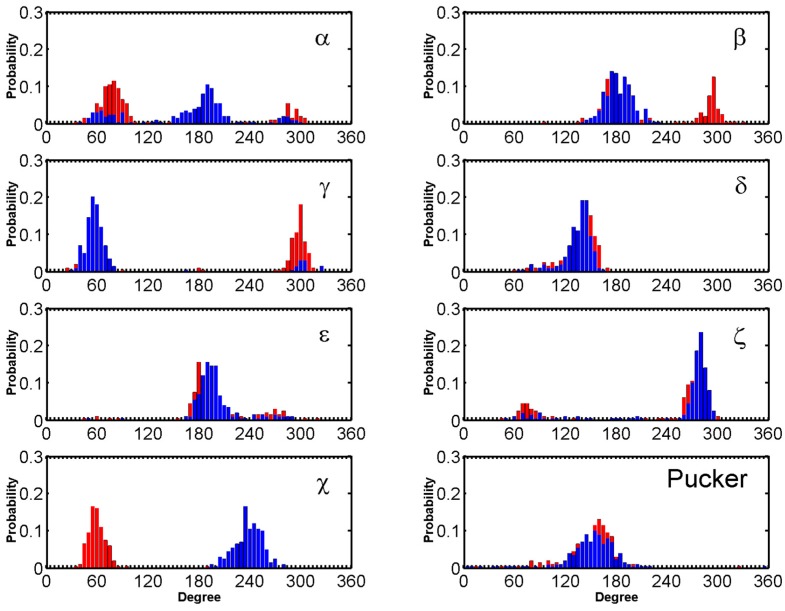
Histograms comparing the backbone torsion angles of residue T6 between the free ligand binding simulation (red) of the top stacking mode of the parallel G-quadruplex and the stability simulation of the crystal structure (black) of the parallel G-quadruplex within last 200 ns.

Major fluctuations were observed in the terminal residues, however T6 in particular is discussed here as it demonstrates highest fluctuation. T6 flipped out at 15 ns and flipped back at 45 ns, flipped out at 69 ns and flipped in at 100 ns and it finally flipped out at 114 ns and remained same throughout the rest of the trajectory. This flipping out of the base is mainly characterized by α, β, γ and χ (Figure 6). Another example of BRACO19 binding to the parallel scaffold facilitated by base flipping is illustrated in Figure S30A which shows the terminal residue A1 clearly flipping outward which provided adequate space for BRACO19 to bind to the top G-quartet, closest to the 5′ end.

### 2.5. BRACO19 Binds to the Anti-Parallel Telomeric G-Quadruplex DNA, with Inducing DNA Structure Fluctuation in Some Trajectories

**Figure 7:**
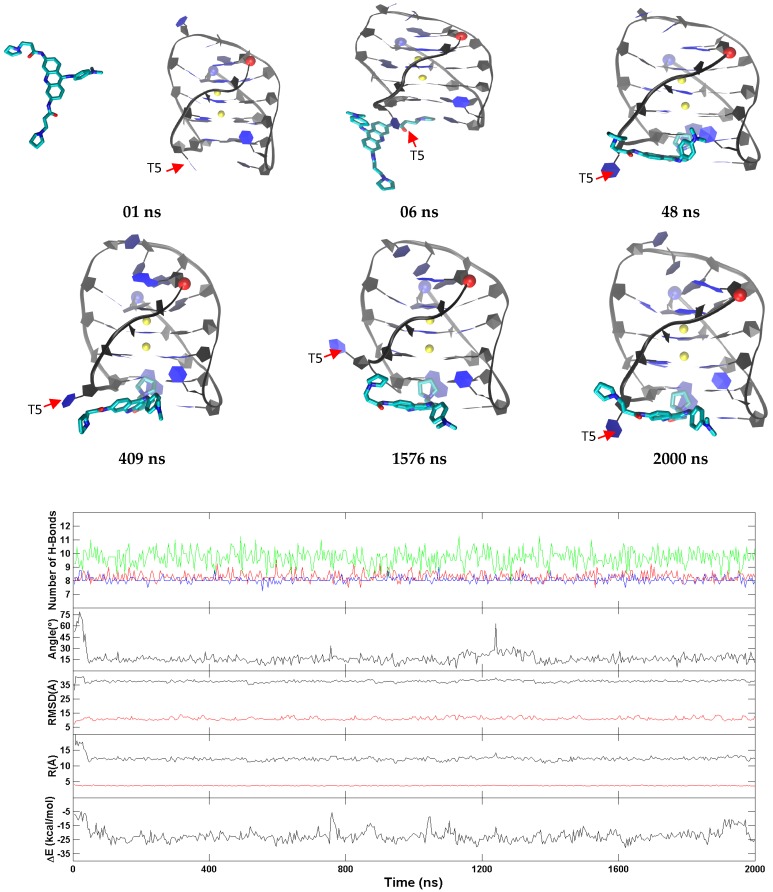
A representative bottom stacking trajectory of the anti-parallel G-quadruplex. (**Top**) Representative structures with time annotation. 5′ and 3′ are indicated by a red and blue ball, respectively. K^+^ ions are represented in yellow. (**Bottom**) An order parameter plot depicting number of hydrogen bonds present in first (red), second G4 (cyan), third G4 (blue), fourth G4 (black) and fifth (green) layers of the DNA structure, the drug-base dihedral angle, receptor (red) and ligand (black) RMSD relative to the original crystal pose, center-to-center distance (R/black) and K^+^-K^+^ distance (R/red) and MM-GBSA binding energy (ΔG) (cf. methods section for definition).

The representative trajectories for the three major binding modes of BRACO19 to the anti-parallel human telomeric G-quadruplex DNA are characterized in Figure 7 and Figure S23 (the bottom stacking mode) and Figure S22 (the top binding mode). In all ten binding trajectories, the DNA showed high structural fluctuation in four trajectories with RMSD of 3.2 Å (Figure S6), the hydrogen bonds in the three G-tetrads were maintained and the distance between K^+^ ions remained stable in all trajectories. The representative trajectory of the bottom stacking mode of BRACO19 (Figure 7) on the human telomeric anti-parallel DNA G-quadruplex showed an initial interaction at 5 ns. The final binding pose was achieved within 48 ns and was maintained throughout the rest of the trajectory. The limited structural dynamics were explained by the limited fluctuation in the five order parameters. The representative trajectory for the top binding mode (Figure S22) is similar to the bottom binding trajectories with a rapid binding and limited fluctuation of order parameters with first interaction at 5 ns and attainment of the final binding pose by 55 ns. The binding energy for bottom stacking and groove binding fluctuated between −55 and −65 kcal/mol while top stacking varied between −40 and −50 kcal/mol after attaining a steady binding pose.

**Figure 8:**
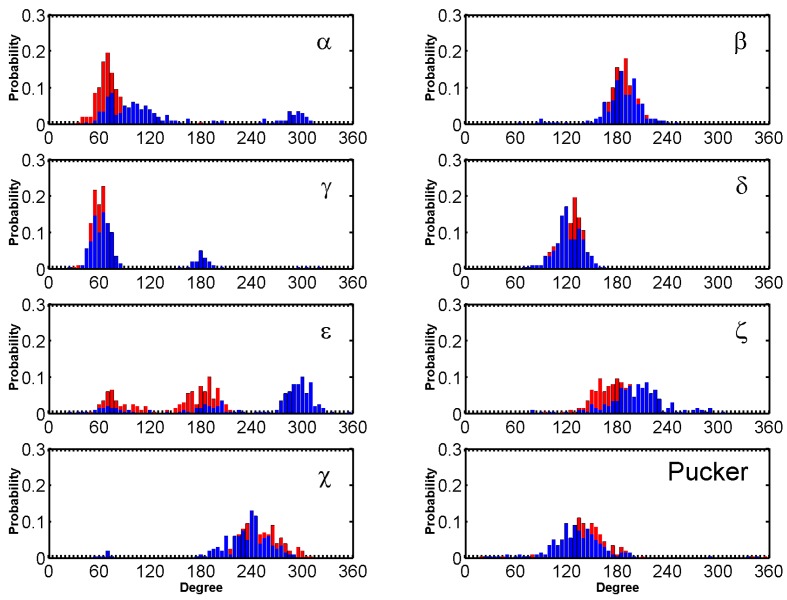
Histograms comparing the backbone torsion angles of residue T05 between the free ligand binding simulation (red) of the top stacking trajectory of the anti-parallel G-quadruplex and the stability simulation of the crystal structure (black) of the anti-parallel G-quadruplex.

The dihedral angles of the G-tetrads in free ligand binding simulations indicate low fluctuations and are consistent through the binding process. Major fluctuations were observed in the terminal residues, T5 in particular is discussed here as it demonstrates highest fluctuation. Through the binding process BRACO19s major interaction was with T5, which opened up as BRACO19 approached and at 29 ns flipped out to let BRACO19 in and flipped back at 40 ns and staying open after interacting with BRACO19. This flipping out of the base is mainly characterized by ε and ζ (Figure 8).

Another example of this base flipping for the anti-parallel topology is illustrated in Figure S30B, where BRACO19′s major interaction is with base A7. As a result of BRACO19′s interaction with base A7, the base T5 flips upward allowing base A7 to flip to the outside of BRACO19 maximizing the binding interactions between the G-quadruplex and BRACO19. The base T5 remains flipped up for the remainder of the trajectory and the interaction where A7 is partially intercalating BRACO19 onto the G-quadruplex is also maintained.

### 2.6. BRACO19 Binds to the Hybrid Telomeric G-Quadruplex DNA, Inducing big DNA Structure Fluctuation in Some Trajectories

**Figure 9:**
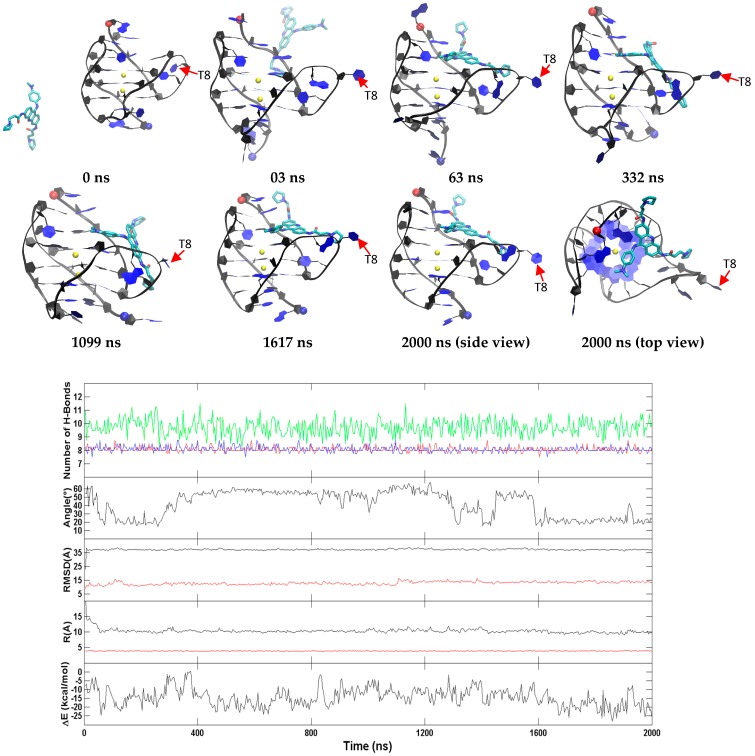
A representative top binding trajectory of the hybrid G-quadruplex. (**Top**) Representative structures with time annotation. 5′ and 3′ are indicated by a red and blue ball, respectively. K^+^ ions are represented in yellow. (**Bottom**) An order parameter plot depicting number of hydrogen bonds present in first (red), second G4 (cyan), third G4 (blue), fourth G4 (black) and fifth (green) layers of the DNA structure, the drug-base dihedral angle, receptor (red) and ligand (black) RMSD relative to the original crystal pose, center-to-center distance (R/black) and K^+^-K^+^ distance (R/red) and MM-GBSA binding energy (ΔG) (cf. methods section for definition).

The representative trajectories for the three-major binding modes of BRACO19 with respect to the hybrid human telomeric G-quadruplex DNA are characterized in Figure 9 and Figure S24 (the top binding mode), Figure S25 (the bottom binding mode) and Figure S26 (the groove binding mode). Out of the ten binding trajectories, the DNA showed high structural fluctuation in five trajectories with RMSD of 2.9 Å (Figure S8), the hydrogen bonds in the three G-tetrads were maintained and the distance between K^+^ ions remained stable in all trajectories. The representative trajectory of BRACO19 top stacking onto the hybrid G-quadruplex DNA showed an initial interaction at 3 ns, the final binding pose was attained as early as 30 ns and was maintained throughout the rest of the trajectory. The limited structural dynamics were explained by the limited fluctuation in the five order parameters. The representative trajectories of the bottom (Figure S25) and groove binding (Figure S26) are similar to the top binding trajectories in rapid binding and limited fluctuation of order parameters. Early interaction of BRACO19 with the quadruplex was at 9 ns and 2 ns respectively and the final binding pose was attained by 51 and 13 ns respectively. The binding energy for all binding modes varied between −55 and −65 kcal/mol after attaining the steady binding pose.

**Figure 10:**
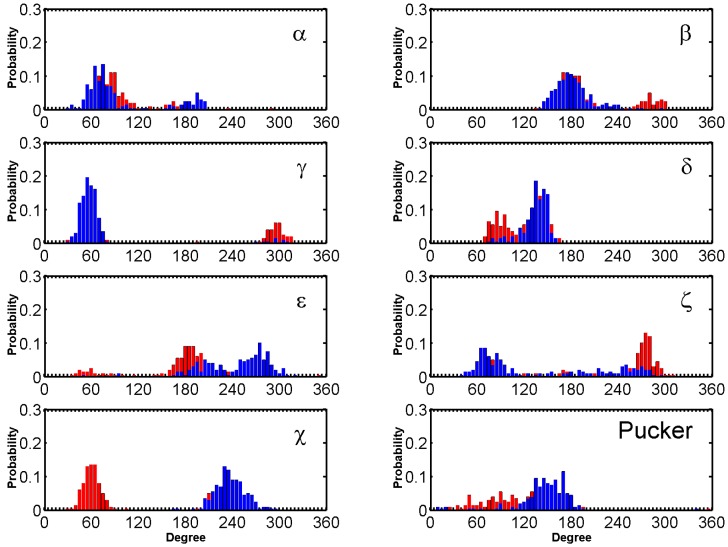
Histograms comparing the backbone torsion angles of residue T8 between the free ligand binding simulation (red) of the top binding trajectory of the hybrid G-quadruplex and the stability simulation of the crystal structure (black) of the hybrid G-quadruplex within the last 200 ns.

The dihedral angles of the G-tetrads in free ligand binding simulations indicate low fluctuations and are consistent through the binding process. Major fluctuations were observed in the terminal residues, T8 in particular is discussed here as it demonstrates highest fluctuation. T8 flipped out upon simulation and remained flipped through a majority of the simulation. This flipping out of the base is mainly characterized by α, δ, ε and ζ (Figure 10). Another example for the hybrid scaffold is presented in Figure S30C. The initial binding of BRACO19 was to the groove of the G-quadruplex before interacting with the 3′ terminal residue which appeared to steer BRACO19 toward a bottom binding interaction. In this trajectory, both the 3′ terminal residue A23 as well as loop residue T13 flip upward (clear in the 48 ns snapshot) which allowed BRACO19 to bind to the bottom of the G-quadruplex. Bases A23 and T13 made slight adjustments in their position for the remainder of the trajectory, whereas BRACO19 remained stably bound to the bottom of the G-quadruplex.

## 3. Discussion

After the discovery of the greater existence of G-quadruplexes in malignant tumors than in normal tissues, the interest in G-quadruplex DNA as a target for cancer therapeutics has increased. BRACO19, an effective G-quadruplex stabilizing ligand, is a promising anticancer drug candidate, yet its low preferential binding affinity (about ~40-fold) to the telomeric single-stranded G-quadruplex DNA over duplex DNA remains to be enhanced. There are the two lines of conflicting evidence on the major target form of BRACO19: (1) under solution conditions with cellular extracts as crowding agents, the more predominate conformations are likely the anti-parallel and/or the hybrid topologies. (2) There is no high-resolution complex structures of BRACO19 binding to antiparallel or the hybrid scaffold, except for parallel stranded. Our binding energy data suggest a hypothesis that reconciles the conflict: the relative population shift of three scaffolds upon BRACO19 binding (i.e., an increase of population of parallel scaffold, a decrease of populations of antiparallel and/or hybrid scaffold). This hypothesis appears to be consistent with the facts that BRACO19 was specifically designed based on the structural requirements of the parallel scaffold and has since proven effective against a variety of cancer cell lines as well as toward a number of scaffolds.

For better molecular insights, the binding of BRACO19 to a duplex 20mer DNA (d([GC]_10_)_2_) and to the parallel, anti-parallel and hybrid telomeric G-quadruplexes were investigated in this study using free ligand binding molecular dynamics simulations. Out of various binding modes for each system, the MM-GBSA binding energy calculations showed that the most stable binding pose was the groove binding mode for the duplex, the top/bottom stacking mode for the parallel G-quadruplex, the bottom stacking mode of the anti-parallel G-quadruplex and the top stacking mode of the hybrid G-quadruplex (Table S1). The order of the relative binding energy of BRACO19 to these DNA forms are as follows: −62.3 ± 4.5 kcal/mol of the top stacking to the parallel G-quadruplex (ΔΔG = 0 kcal/mol), −61.7 ± 8.0 kcal/mol of the groove binding to the duplex DNA (ΔΔG = 0.6 kcal/mol), −53.9.4 ± 5.8 kcal/mol of the bottom stacking to the anti-parallel G-quadruplex (ΔΔG = 8.4 kcal/mol) and −40.5 ± 5.4 kcal/mol of the top stacking to the hybrid G-quadruplex (ΔΔG = 21.8 kcal/mol). For all the systems, breaking down the binding energy indicated that the VDW term makes the biggest contribution to the total binding energy (Table S1). This indication suggests introducing target or drug specific packing optimization as a prospect for further stabilization of the G-quadruplex. A limitation of the MMPBSA binding energy calculations are that they do not include the conformational changes involved in the folding process of the G-quadruplexes nor do they consider the relative stability of the different scaffolds. Because of this, MMPBSA calculations alone may not be sufficient enough to predict the most favorable scaffold under physiological conditions.

With groove binding predicted to be the least energetically favorable, and based on our visual inspection of each trajectory, our data suggests that BRACO19′s groove binding pose is likely not stable enough to maintain a prolonged binding event and that under a more extended timeline the groove binding mode may function as an intermediate state preceding a more energetically favorable end stacking pose. To support this, Figure S29A–C provide representative snapshots of three simulation runs from each G-quadruplex system are presented. As for the anti-parallel system, we attribute the comparable top and groove binding poses to the anti-parallel topology. Based on our observations the diagonal loop (T11, T12, A13) atop the G-quartet, closest to the 5′ terminal, obstructs BRACO19′s ability to achieve a stable stacking pose on the top G-quartet. Therefore BRACO19′s major interaction with the top of the anti-parallel G-quadruplex is with the TTA diagonal loop, which offers no benefit over the groove binding pose in terms of binding interaction.

If these binding modes have comparable entropic energies then our relative binding energies suggest that BRACO19 binds preferentially to the parallel G-quadruplex over the anti-parallel and the hybrid G-quadruplexes if assuming equally abundant conformations in physiological condition. In the same way, our relative binding energy data shows that BRACO19 binds preferentially to the telomeric parallel G-quadruplexes over the DNA duplex. This qualitatively explains the experimental observation of weak preferential binding affinity difference of BRACO19 on the two DNA forms (40-fold of the selectivity). For that reason, it can be suggested that a ligand modification that destabilizes the duplex groove binding mode but stabilizes the G-quadruplex top stacking mode will enhance the binding selectivity of the ligand. For example, adding a rigid planar ring fragment to the acridine may facilitate top stacking rather than groove binding and increase the Van der Waals interactions in turn increasing selectivity and binding affinity of the prospective drug towards the G-quadruplex. This suggestion is consistent with the original SAR data in the development of BRACO19 from prototype BSU6048 in which the addition of the ring at position 9 (makings of BRACO19) increased the drug selectivity from 10-fold to 40-fold towards human telomeric G-quadruplexes over duplex DNA [48,87,88]. The addition of the methylated anilino group, as opposed to the hydrogenated aniline at the 9th position slightly decreased binding to the duplex, while maintaining binding to the G-quadruplex [89]. It is also to be noted that the sidechains on 3 and 6 contribute to the groove binding of both DNA duplex and G-quadruplex which could be the reason behind low selectivity. We observed the side chains of the 3rd and 6th position to play a role in BRACO19′s ability to fully bind to the groove of the duplex DNA, but the side chains appear to play less of a role in G-quadruplex binding; which we primarily observed as an interaction with the acridine core. Thus, suggestions can be made to reduce the length of these side chains. These side chains exist in protonated form at physiological pH however, Table S1 indicates that the contribution of electrostatic interactions to the binding affinity is very low and therefore modifications can be suggested to the substituents at 3rd and 6th position of the acridine. Modifications such as loss of positive charge which would increase the hydrophobicity which could in fact increase the Van der Waals interactions and reduction of the length of the side chains.

**Figure 11:**
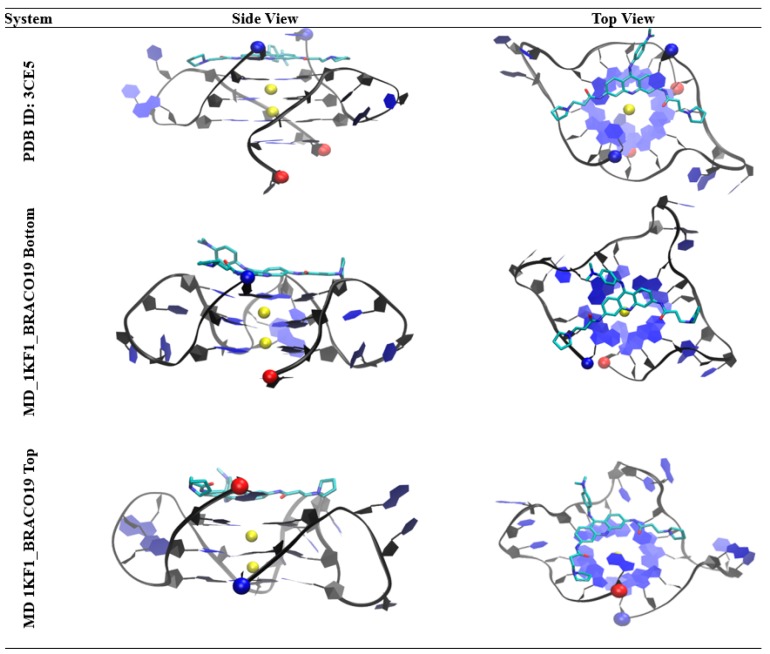
Comparison of the Double Stranded parallel G-quadruplex-BRACO19 complex (PDB ID: 3CE5) and both the Bottom and Top Binding Modes of the Single Stranded parallel G-quadruplex-BRACO19 complex. The 5′ residues are represented by a red ball and the 3′ residues in blue.

Encouragingly, the binding pose of BRACO19 to the parallel human telomeric G-quadruplex is consistent with to the only available crystal structure of BRACO19 (PDB ID: 3CE5) (Figure 11). In both the crystal structure and the structure from our study, the acridine core binds to the G-quartet closest to the 3′ terminal with the nitrogen from the acridine core facing inward in-line with the K^+^ cations. The anilino group at the 9th position faces away from the G4 core and the two substituents at the 3rd and 6th position are also positioned outward, allowing the drug to remain planar and stack onto the G-quartet. Although a top binding pose was not reported in the crystal structure of the double stranded parallel topology, our study suggests due to the symmetry of the single stranded parallel G-quadruplex topology both the top and bottom of the G-quadruplex core offer comparable binding opportunities for BRACO19. In support of this, our MM-GBSA analysis showed the most energetically favorable top and bottom binding modes were within 0.5 kcal/mol; where the top binding pose (−62.3 ± 4.5 kcal/mol) was slightly more favorable than the bottom binding pose (−61.8 ± 1.5 kal/mol). Our study also clearly showed BRACO19 in a top binding pose closely matching the description published for the bottom stacking in the crystal structure (Figure 11). Together this provides evidence to support that both end stacking modes could offer equal binding for BRACO19 in the single stranded parallel scaffold of the human telomeric DNA G-quadruplex. In addition to this, a crystal structure of a Pt-tripod in complex with the hybrid DNA G-quadruplex sequence was recently solved (PDB ID: 5Z80), which shows binding to the top of the G4 similar to the binding pose observed in our study (Figure S28).

Although longer simulation periods are required to confirm, evidence of an induced fit binding mechanism was observed in each of the BRACO19-G-quadruplex complexes. The representative trajectories in Figure 5, Figure 7 and Figure 9 show one example per system where a base flipping mechanism worked to enhance the binding of BRACO19 to the receptor. The base flipping mechanism was ongoing and recurrent through conformational changes that occurred during the binding event. Despite an absolute equilibrium not being reached under the restricted simulation period, the persistent use of the base flipping mechanism and the resultant beneficial binding interactions, as observed over the timeline of the MMGBSA energy analysis, suggest the potential use of an induced fit binding mechanism facilitated by base flipping. We also provide a detailed analysis of the dihedral angles of the residue showing the largest fluctuation in each system compared to the apo form. The dihedral angles helped to characterize the changes of the bases that may contribute to an induced fit binding mechanism. Including a description of the binding events of a second example from each system illustrated in Figure S30. This figure demonstrates three important characteristics that suggest the use an induced fit binding mechanism used by BRACO19. For the parallel topology (Figure S30A), the flipping out of the 5′ terminal base A1 led to the repositioning of BRACO19 on top of the top G-quartet. We observed two possible outcomes for the mechanism involving 5′ terminal base flipping: (i) the 5′ base will flip back on top of BRACO19 intercalating it onto the top G-quartet or (ii) the 5′ terminal base will position itself in plane with BRACO19 and base pair; both mechanisms enhance the binding interactions between BRACO19 and the DNA G-quadruplex. The anti-parallel DNA G-quadruplex (Figure S30B) provides an example of two bases from the same loop changing position in order to enhance the binding of BRACO19 to the DNA G-quadruplex. In this case, the flipping upward of base T5 during the simulation run allowed base A7 to flip outward and reposition itself around the outside of BRACO19 so that BRACO19 was partially intercalated to the groove of the DNA G-quadruplex which maximized its binding interactions. The hybrid topology (Figure S30C) provides an example of both a terminal and loop residues flipping outward to allow BRACO19 to reposition into a binding pose that enhances its binding interactions. In this case, the 3′ terminal residue A23 and loop residue T13 both flip outward allowing BRACO19 to stack to the bottom of the G-quadruplex. Residue A23 flips back on top of BRACO19 intercalating it while T13 remains flipped outward to provide sufficient room for BRACO19. Together with the analysis of the dihedral angles, the flexibility of both the terminal and loop residues -which through their conformational changes allow BRACO19 to positon itself in a more favorable binding pose and enhance its binding interactions show characteristics of an induced fit binding mechanism. It was by use of the free ligand MD binding simulations, as opposed to rigid body docking, that we were able to observe the flipping of the terminal and loop bases during the binding process which we suggest are integral for BRACO19 to achieve the most favorable binding pose.

## 4. Methods

### 4.1. Simulation Systems

A total of nine systems were constructed: a BRACO19 only system, a B-DNA duplex structure of d([GC]_10_)_2_, the X-ray crystal structure of the parallel telomeric DNA G-quadruplex, the NMR solved anti-parallel telomeric DNA G-quadruplex and the NMR-solved hybrid telomeric DNA G-quadruplex and four DNA-ligand systems (Table 2). The B-DNA duplex structure of d([GC]_10_)_2_ was built using Maestro program. The four free ligand-DNA systems were constructed with a free BRACO19 molecule that was 10 Å away from the DNA (Figure S1). A water box of truncated octahedron with 10 Å water buffer was used to solvate the unbound system and was neutralized by K^+^ and 0.15 M KCl was added. The DNA structures were represented by a refined version of the AMBER DNA OL15 (i.e., parm99bsc0 [90] + χ_OL4_ [91] + ε/ζ_OL1_ [92] + β_OL1_ [93] updates), water was represented by TIP3P model [94] and the K^+^ ions were represented by the K^+^ model developed by Cheatham group [95]. The standard AMBER protocol was used to obtain the force field for the BRACO19 molecule: after the geometry optimization of BRACO19 at the HF/6-31G* level, the molecular electrostatic potential (MEP) of the BRACO19 molecule was calculated at the same theory level; then the partial charges of BRACO19 atoms were determined by MEP using Restrained Electrostatic Potential/RESP method with two stage fitting [96]; and the AMBER GAFF2 [97] force field provided the rest of the force field parameters. The supporting document of our previous paper [85] provides the BRACO19 force field in Mol2 format. The nucleic acid simulations have been widely practiced in AMBER DNA force fields [81,98,99,100,101]. In our previous studies, the binding pathway of doxorubicin [101] and Telomestatin [83], anti-cancer drugs to the B-DNA fragment [102] and to the human telomeric hybrid G-quadruplex [83], respectively have been simulated.

### 4.2. Simulation Protocols

The ten production runs for all complex systems were conducted using the AMBER 16 simulation package [97]. The detailed protocol followed our previous studies [83,102]. After minimizing the energy, the Maxwell-Boltzmann distribution was used to conduct all simulation runs with different initial velocities, which were assigned based on random seeds. For the free binding system, an extra 500 ps pre-run at high temperature (500 K) was carried out to randomize the position and orientation of the free ligand, while the receptor was fixed. Better sampling of binding poses and pathway was enabled by multiple independent simulations. To equilibrate the system density, a short 1.0 ns MD simulation in the NPT ensemble mode (constant pressure and temperature) was conducted, where the DNA and ligand were subjected to Cartesian restraints (1.0 kcal/mol/Å). For the 500 ns production run, the NVT ensemble mode (constant volume and temperature) was used to enhance the simulation code stability. The representative trajectory for major binding modes was picked from each system and extended to 2000 ns. All bonds connecting hydrogen atoms were constrained by SHAKE [103] which enabled a 2.0 fs time step in the simulations. Long-range electrostatic interactions under periodic boundary conditions were treated using the particle-mesh Ewald method [104] (the fourth order of the B-spline charge interpolation, charge grid spacing of ~1.0 Å; and direct sum tolerance of 10^−5^). The cutoff distance for short-range non-bonded interactions was 10 Å, with the long-range Van der Waals interactions based on a uniform density approximation. To reduce the computation cost, a two-stage RESPA approach [105] was used to calculate non-bonded forces where the short range forces was updated once per time step and the long range forces was updated twice per time step. The Langevin thermostat with a coupling constant of 2.0 ps was used to control the temperature. The trajectories were saved at 50.0 ps intervals for analysis.

### 4.3. Convergence of Simulations

The initial structure was used as a reference to calculate the root mean square deviation (RMSD) of DNA backbone. The stability of the DNA structures was indicated by the flat and small RMSDs (Figures S2, S4, S6 and S8). An atom-to-atom distance cutoff of 3.0 Å was used to calculate atom contacts between the DNA structure and BRACO19. The stable contact number indicated the steady state of the simulation systems (Figures S3, S5, S7 and S9). We defined a stable complex as one with greater than 10 atom contacts.

### 4.4. Binding Mode Identification

Accounting to the stability of the DNA backbone in the binding process, the DNA backbone of the stable complexes was aligned by a least square fitting. Daura algorithm [106] was used to cluster the aligned complexes into different structural families based on the 2 Å pair-wise RMSD cutoff of the BRACO19 only without ligand fit. The centroid structure was defined as a structure with the largest number of neighbors in the structural family and was used to represent that structural family. Based on visual inspection, super-families corresponding to major binding modes were formed by merging the centroid structures (Figure 3; Figures S14–S17).

### 4.5. Order Parameters to Characterize DNA-Drug Binding Pathway

The DNA-drug binding process was characterized by using five order parameters: hydrogen bond analysis, drug-base dihedral angle, DNA/ligand RMSD, center-to-center and K^+^-K^+^ distance (R) and MM-GBSA binding energy (ΔG). A hydrogen bond was defined by 3.5 Å distance cutoff between H-bond donor and H-bond acceptor and 120° donor-H-acceptor angle cutoff. The hydrogen bonds were calculated for the top/first, middle/second and bottom/third base tetrads. For the three G-quadruplexes, the three G-tetrads were defined so that 5′ is close to the first G-tetrad. The dihedral angle between the plane of the stable G-tetrad of the DNA that is close to drug binding site and the BRACO19′s ring plane was defined as the dihedral angle. After aligning the DNA, the ligand RMSD was calculated with reference to the first frame of the trajectory. The length from the DNA center to the drug molecule center was defined as the center-to-center distance (R). The distance between the K^+^ ions present in the DNA G-quadruplex was defined as K^+^-K^+^ distance. The energetics of the bound complexes were analyzed using the Molecular Mechanics Generalized Born-Surface Area (MM-GBSA) ^54^ module in the AMBER package (GB1 model with salt concentration of 0.15 M, mBondi radii set, and surface tension of 0.0072 kcal/Å^2^) to avoid the large energy fluctuation of the explicit solvent.

It was reported that even when considering the relative solvation free energy, good predictions can be made for charged molecules by the GB models on the hydration free energy [107]. Under this assumption, ions were removed from charged DNA systems in this study. This approach was validated in our previous study, in which this MM-GBSA protocol successfully assessed the binding energy of doxorubicin, an anti-cancer drug, to a B-DNA fragment (d(CGATCG)2) [102]. Under comparable entropic terms, the relative binding free energy estimated by the MM-GBSA binding energies can be used to rank drugs or their binding poses if a single molecule is considered [106]. It has been established by systematic benchmarking studies up to 1864 crystal complexes that ranking of the ligand binding affinity can be achieved by relative MM-GBSA binding energy calculations [108,109,110]. In a previous work we studied the use of MM-GBSA versus MM-PBSA as a predictor of BRACO19′s relative binding energy (ΔΔG) over a range of ionic strengths [85]. The highly comparable relative binding energies in both the MM-GBSA and MM-PBSA calculations supports the use of MM-GBSA in ranking the binding poses of BRACO19 in this study. The MM-GBSA binding energy for each system was calculated from three simulations [84]: ligand only, DNA only and DNA-ligand complex using Equation (1). Equation (2) is made up of four components: Van der Waals interaction energy (VDW), hydrophobic interaction energy (SUR), electrostatic interaction (GBELE) and the change of the conformation energy for DNA and ligand. These terms were calculated using Equations (3) and (4).

(1) $$\Delta E=E_{complex}-E_{DNA\_free}-E_{lig\_free}$$

(2) $$\Delta E=\Delta E_{vdw}+\Delta E_{SUR}+\Delta E_{GBELE}+\Delta E_{comformation}$$

(3) $$\Delta E_{x}=E_{x\_complex}-E_{x\_DNA\_complex}-E_{x\_lig\_complex},\;x=VDW,\;SUR\;and\;GBELE$$

(4) $$\Delta E_{Comformation}=E_{DNA\_from\_complex}+E_{lig\_from\_complex}-E_{DNA\_free}-E_{lig\_free}$$

The standard backbone dihedral angles (α, β, γ, δ, ε and ζ) around the covalent bonds of the deoxyribose and χ about the glycosidic bond were defined (Figure S27) to characterize the conformational changes.

## 5. Conclusions

The detailed structural knowledge of the intramolecular human telomeric G-quadruplexes in complex with a ligand is required for the rational design of human telomeric G-quadruplex binding drugs. Despite high interest in the G-quadruplex binder BRACO19, a detailed binding mechanism remains to be solved. In this study, free ligand molecular dynamics binding simulations were used to probe and understand the binding nature of BRACO19, a potent human telomeric G-quadruplex drug, to a B-DNA duplex and the three scaffolds of a single stranded human telomeric G-quadruplex. The most stable binding mode, indicated by the MM-GBSA binding energy analysis, for the duplex DNA is the groove binding mode, end stacking for the parallel G-quadruplex, bottom stacking for the anti-parallel G-quadruplex and top stacking of hybrid G-quadruplexes. The similar binding affinity of BRACO19′s groove binding mode with respect to both the duplex and the G-quadruplexes may explain its lack of preferential binding selectivity. Therefore, a ligand modification that destabilizes the duplex groove binding mode but stabilizes the G-quadruplex top stacking mode will improve the binding selectivity of the ligand. Encouragingly, our study was able to produce both a top and bottom binding mode that closely matched the description of BRACO19 in complex with a double stranded parallel scaffold; the only available complex crystal structure with BRACO19. Our study suggests that both ends of the single stranded parallel scaffold offer equal binding opportunity for BRACO19 and is further supported by the 0.5 kcal/mol absolute binding energy difference (ΔΔG) which slightly favors the top stacking pose over the bottom. For the hybrid scaffold, BRACO19 was observed in a comparable binding pose to the crystal structure of a Pt-tripod in complex with the same hybrid scaffold of the human telomeric DNA G-quadruplex. Consistent use of a base flipping mechanism provided evidence of an the use of an induced fit binding mechanism through the flexibility of both terminal and loop residues that underwent conformational changes ultimately enhancing the binding interactions between the DNA G-quadruplexes and BRACO19. From our analysis of the base flipping we identify two primary outcomes: (i) the base that flips outward will flip back after BRACO19 repositions and enhance interactions through base pairing or intercalation interactions, (ii) the base that flips outward provides sufficient room for BRACO19 to reposition and enhance its binging interactions with other bases of the G-quadruplex. Our study presents a successful example of the ability of molecular dynamic simulations with the latest AMBER force field to facilitate detailed structural and dynamic information which will further decipher the binding nature of DNA ligands.

## Figures and Tables

**Table 1:** Molecular dynamics simulations.

System ID	DNA	No. of Ligand	No. of Run	Drug Initial State	NPT eq. (ns)	NVT (ns)	Total Time (µs)
1	n/a	1	2	Free	1	500	1
2	Duplex(d([GC]_10_)_2_)	0	2	Free	1	500	1
3	3(-p-p-p) (1KF1)	0	2	Free	1	500	1
4	3(-lwd+ln) (143D)	0	2	Free	1	500	1
5	3(-p-lw-ln) (2HY9)	0	2	Free	1	500	1
6	Duplex(d([GC]_10_)_2_)	1	9 + 1	Free	1	500 + 2000	6.5
7	3(-p-p-p) (1KF1)	1	8 + 2	Free	1	500 + 2000	8.0
8	3(-lwd+ln) (143D)	1	9 + 1	Free	1	500 + 2000	6.5
9	3(-p-lw-ln) (2HY9)	1	9 + 1	Free	1	500 + 2000	6.5

**Table 2:** In vivo activity of BRACO19 against various cancer cell lines.

Cell Lines	Tissue Type	IC_50_	References
MCF7	Breast cancer (human)	2.5 μM	[55,56]
A549	Lung cancer (human)	2.4 μM	[55,57]
DU145	Prostate cancer (human)	2.3 μM	[55,58]
HT-29	Colon cancer (human)	2.7 μM	[55,59]
HGC-27	Gastric carcinoma	2.6 μM	[55,60]
A2780	Ovarian cancer (human)	2.5 μM	[55,61]
WI-38	Lung fibroblast (human)	10.7 μM	[55,62]
IMR90	Lung fibroblast (human)	>25 μM	[55,63]
U87	Glioblastoma (human)	1.45 μM	[64,65]
U251	Glioblastoma (human)	1.55 μM	[64]
SHG-44	Glioma (human)	2.5 μM	[64]
UXF1138L	Uterus carcinoma (human)	2.5 μM	[66]
CH1	Lymphoma (mouse)	10.1 μM	[67]
SKOV3	Ovarian cancer (human)	13.0 μM	[67,68]
CLL	Chronic lymphocytic leukemia	80 μM	[69,70]
AML	Acute myeloid leukemia	80 μM	[70]
--	Prolymphocytic leukemia	80 μM	[70]

## Supplementary Materials

Supplementary Materials are available online. The structure pdb file and the movie file of the most stable binding pose for each DNA system is included in the supporting material. Also available is the summary of BRACO19s activity against various cancer cell lines, RMSD and ligand-DNA contact plot for each trajectory, representative structures of the most populated structural families for each system, additional representative trajectories are included in the supporting document.
