# Supplementary material for: Binding of BRACO19 to a Telomeric G-Quadruplex DNA Probed by All-Atom Molecular Dynamics Simulations with Explicit Solvent
Source: Molecules. 2019 Mar 13;24(6):1010. doi: 10.3390/molecules24061010 (PMC6471034; doi:10.3390/molecules24061010)

Supporting Information for "Binding of BRACO19 to a Telomeric G-Quadruplex DNA Probed by All-Atom Molecular Dynamics Simulations with Explicit Solvent" by Babitha Machireddy[1], Holli-Joi Sullivan[1] and Chun Wu*

Table S1. MM-GBSA binding Energy (kcal/mol) of BRACO19 to human telomeric DNA duplex and Quadruplexes.

System	Pose	[1]ΔE_{VDW}	[2]ΔE_{SUR}	[3]ΔE_{GBELE}	[4]ΔE_{CONF}	[5]ΔG_{TOT}	[6]$\Delta\Delta G_{TOT}$
**DNA Duplex**	Top Stacking	-28.9±4.0	-2.3±0.5	-5.2±3.1	2.7±1.2	-33.7±5.3	28.6
	Bottom Stacking	-28.4±4.1	-2.2±0.5	-4.8±3.2	0.8±3.8	-34.6±5.7	27.7
	Groove Binding	-57.9±9.5	-5.2±0.7	-3.9±4.5	5.2±2.8	**-61.7±8.0**	**0.6**
**Parallel G4**	Top Stacking	-63.1±5.7	-5.2±0.6	-1.9±4.4	7.9±5.2	**-62.3±4.5**	**0**
	Bottom Stacking	-44.5±3.3	-3.79±0.2	-11.35±2.0	-2.2±3.9	**-61.8±1.5**	0.5
	Groove Binding	-37.0±6.4	-3.1±0.4	-8.9±4.5	11.4±4.5	-37.6±7.2	24.7
**Anti-parallel G4**	Top Stacking	-29.1±9.0	-2.5±0.8	-9.2±3.0	-2.1±4.4	-42.8±4.1	19.5
	Bottom Binding	-41.5±11.4	-4.0±1.1	-8.5±4.3	0.1±4.6	**-53.9±5.8**	**8.4**
	Groove Binding	-43.0±6.0	-3.4±0.5	-7.5±2.6	10.9±2.3	-43.1±7.2	19.2
**Hybrid G4**	Top Stacking	-44.2±11.4	-4.3±1.0	-12.1±5.0	20.0±9.2	**-40.5±5.4**	**21.8**
	Bottom Binding	-25.7±5.8	-2.8±0.7	-16.3±6.0	15.8±8.5	-29.0±12.9	33.3
	Groove Binding	-40.5±6.6	-4.0±0.5	-14.9±5.2	23.7±3.6	-35.7±5.1	26.6

[1] Change of Van der Waals energy in gas phase upon complex formation
[2] Change of surface area term change upon complex formation
[3] Change of GBELE generalized Born term + gas phase electrostatic energy upon complex formation
[4] Change of conformational energy upon complex formation
[5] Change of total potential free energy in water upon complex formation (VDW+SUR+GBELE+CONF)
[6] Change in binding free energy in reference to parallel G-quadruplex top binding

DNA Duplex + Unbound BRACO19	3(-p-p-p) DNA G-Quadruplex + Unbound BRACO19
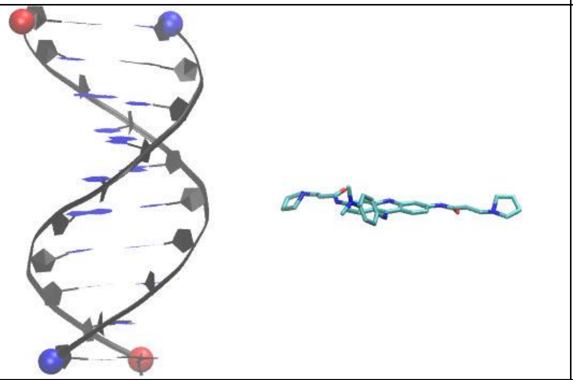	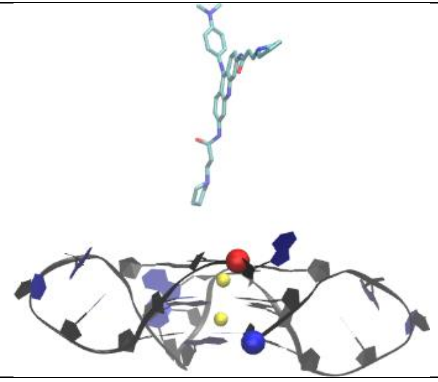
3(-lwd+ln) DNA Quadruplex + Unbound BRACO19	3(-p-lw-ln) DNA Quadruplex + Unbound BRACO19
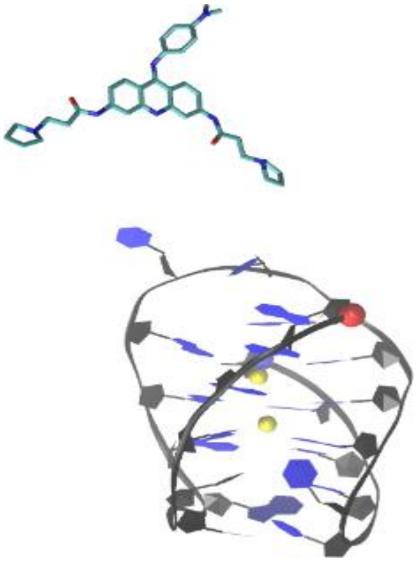	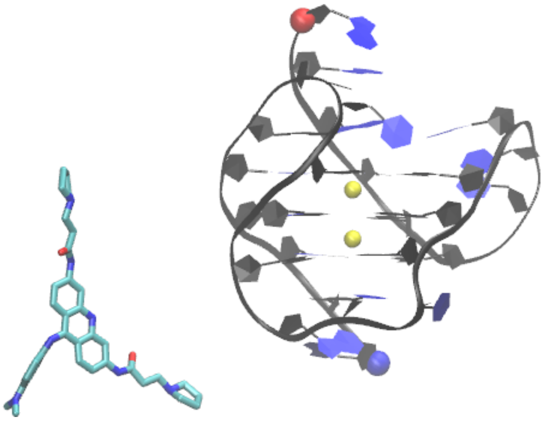

Figure S1. Initial structures of the simulation systems. 5’ and 3’ are indicated by a red and blue ball, respectively.

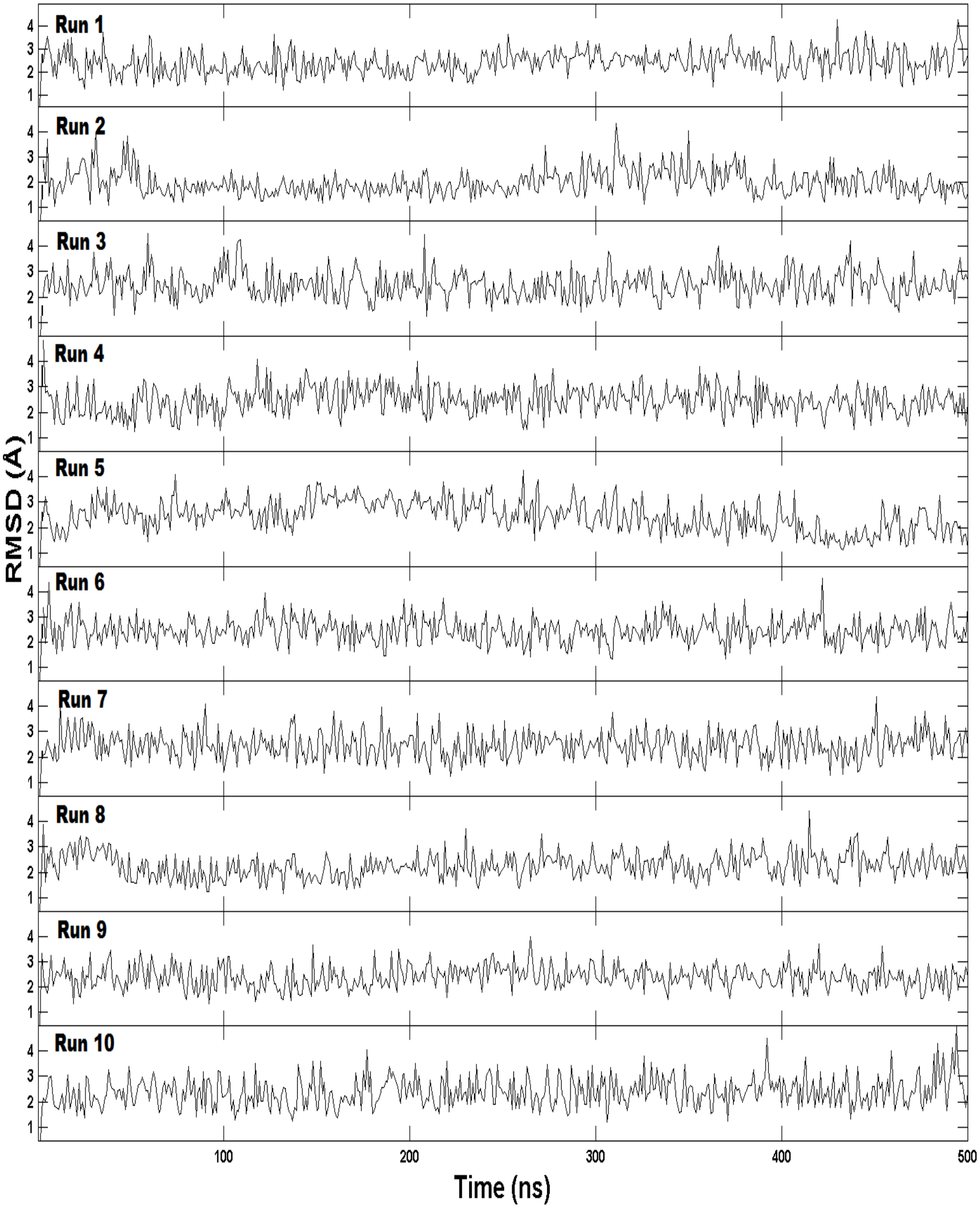

Figure S2. RMSD of the BRACO19-duplex DNA complex in each trajectory.

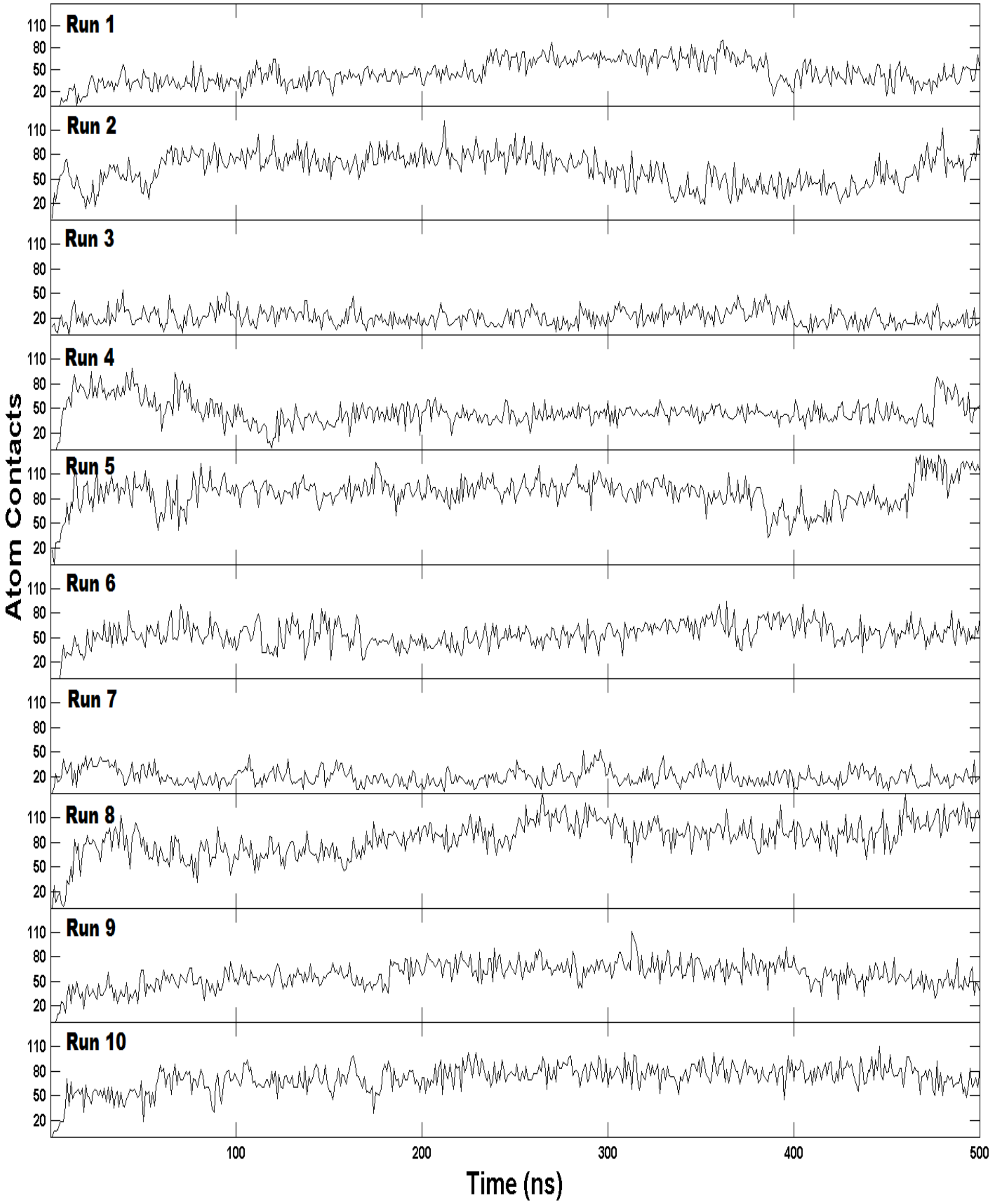

Figure S3. The contact number between BRACO19 and the duplex DNA in each trajectory.

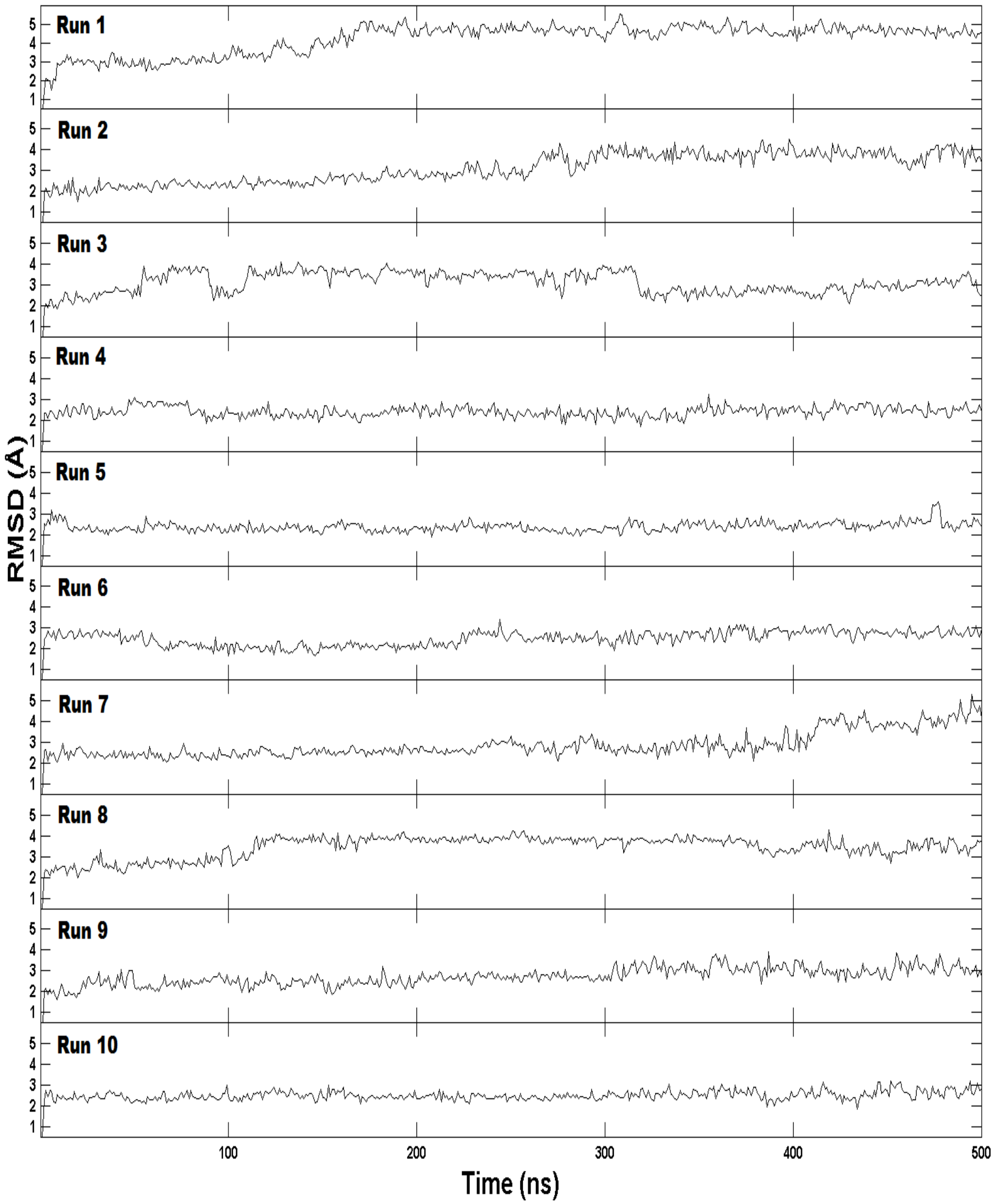

Figure S4. RMSD of the parallel topological fold of DNA in complex with BRACO19 in each trajectory.

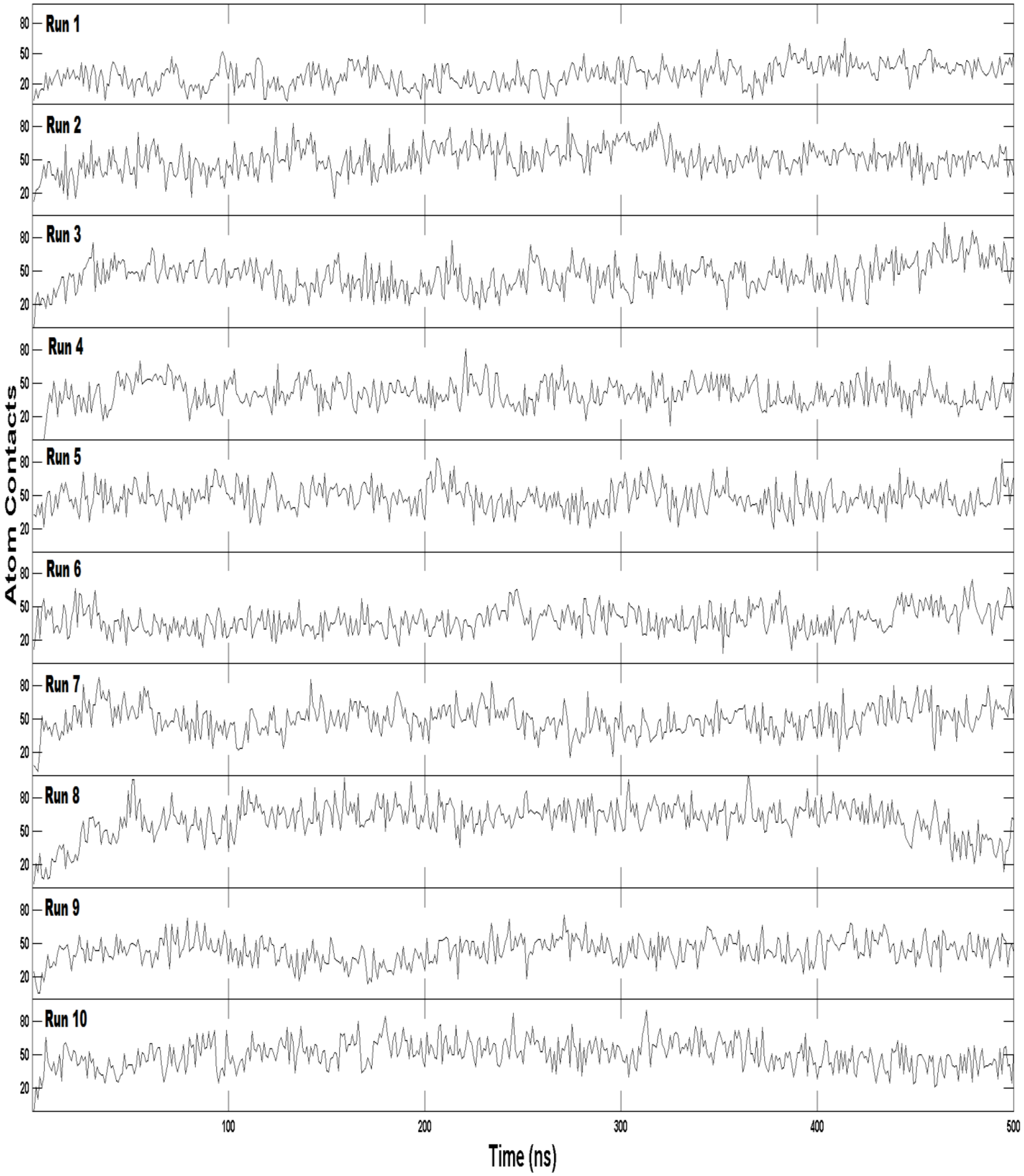

Figure S5. The contact number between the parallel topological fold of DNA and BRACO19 in each trajectory.

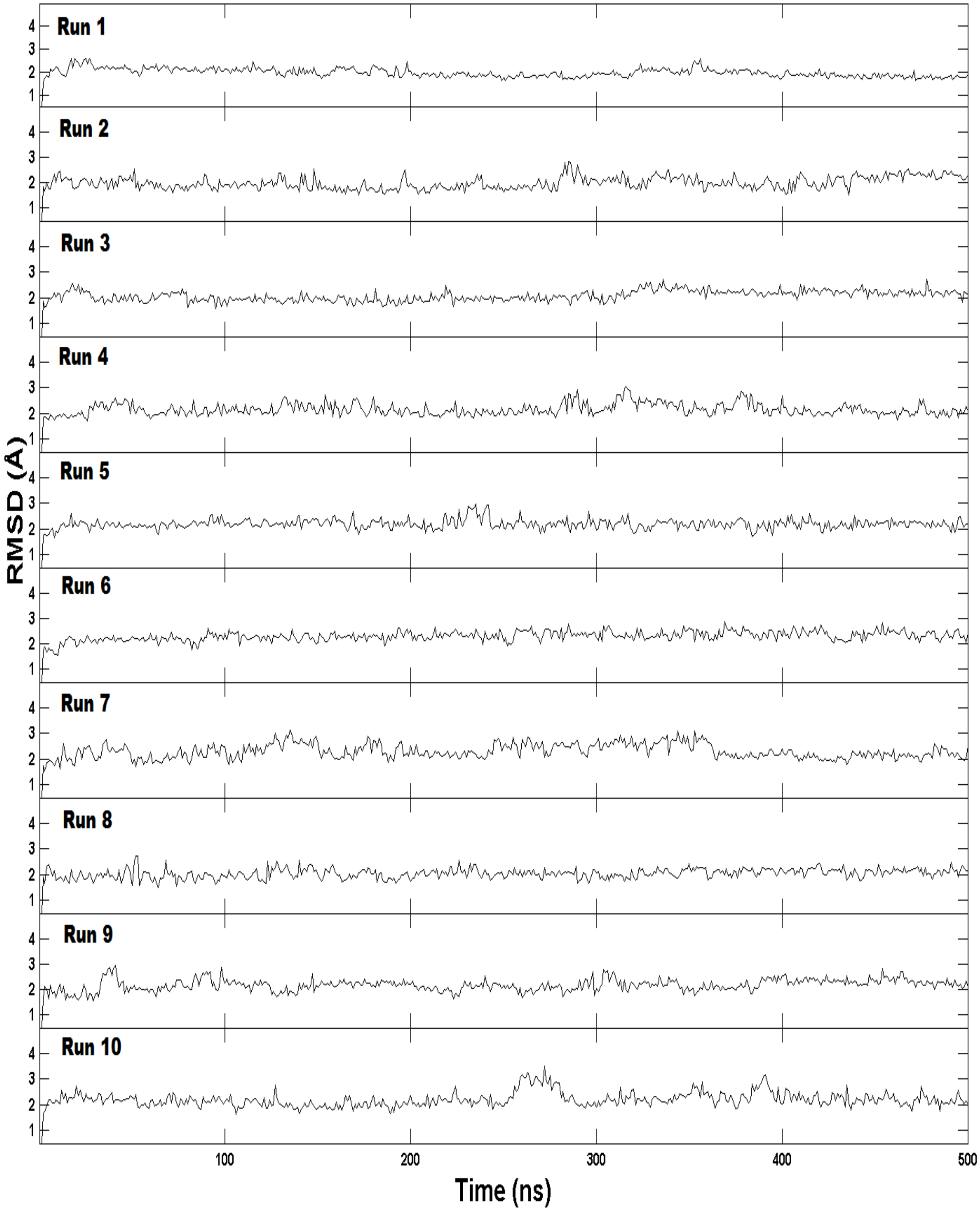

Figure S6. RMSD of the anti-parallel topological fold of DNA in complex with BRACO19 in each trajectory.

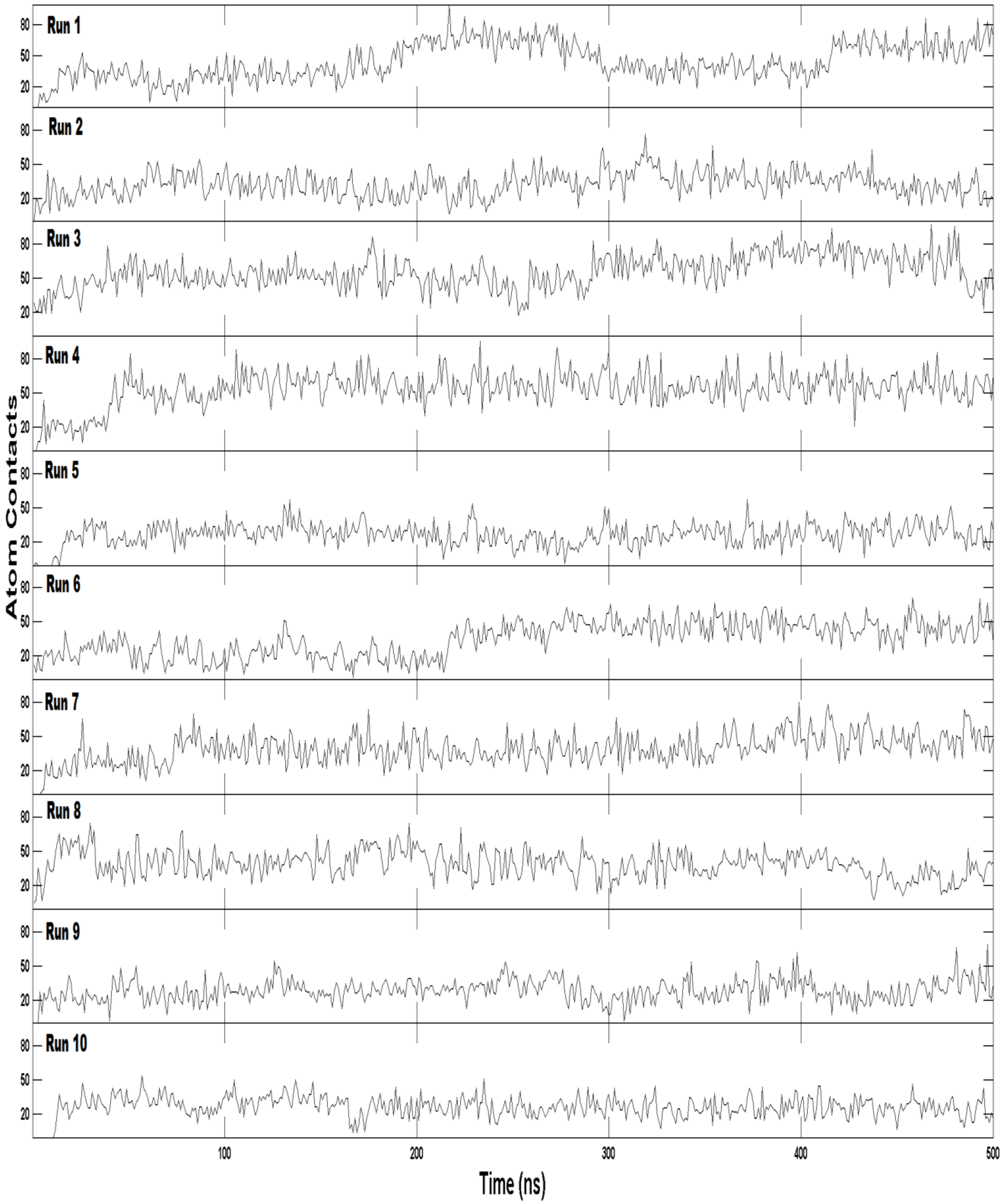

Figure S7. The contact number between the anti-parallel topological fold of DNA and BRACO19 in each trajectory

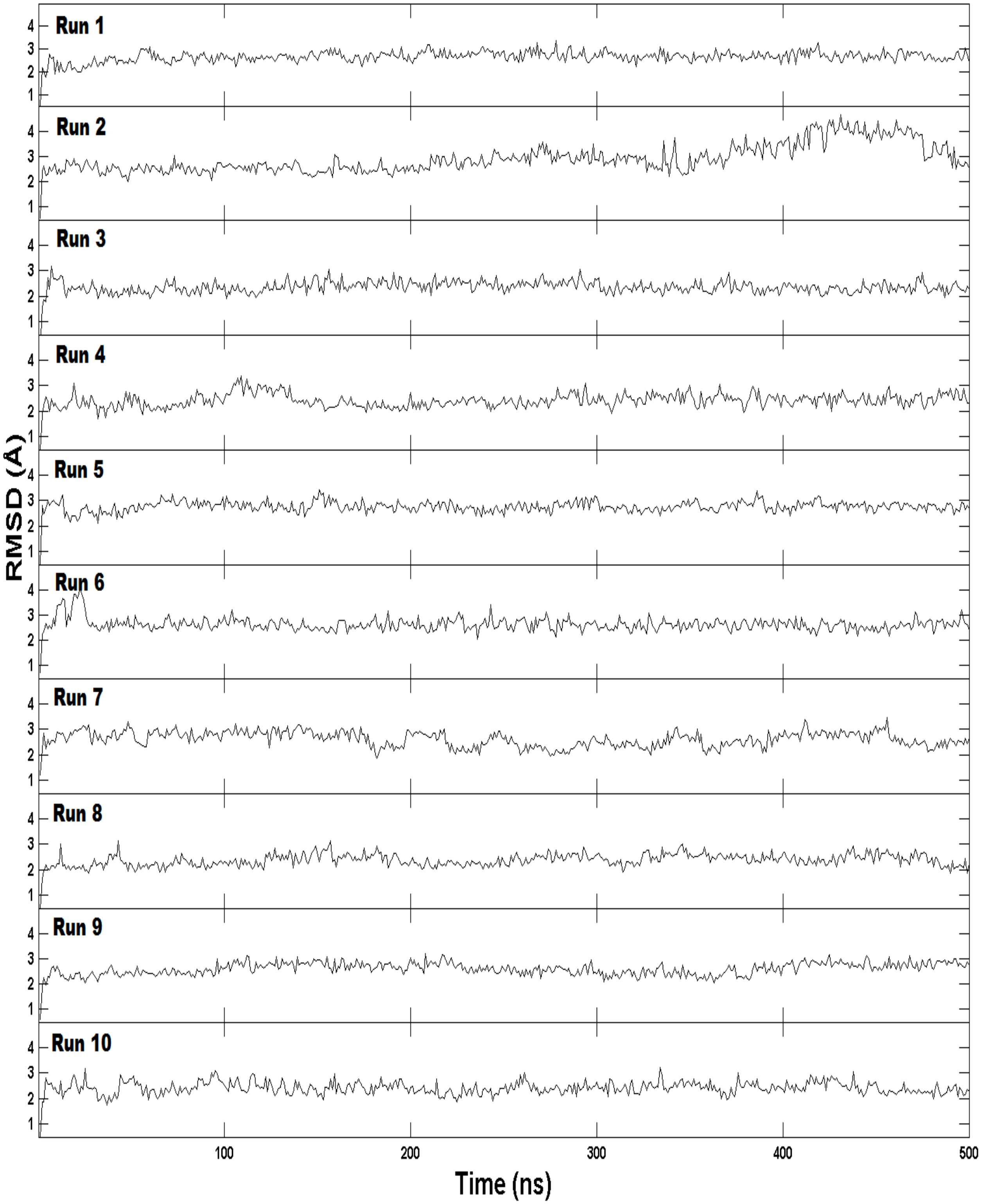

Figure S8. RMSD of the hybrid topological fold of DNA in complex with BRACO19 in each trajectory.

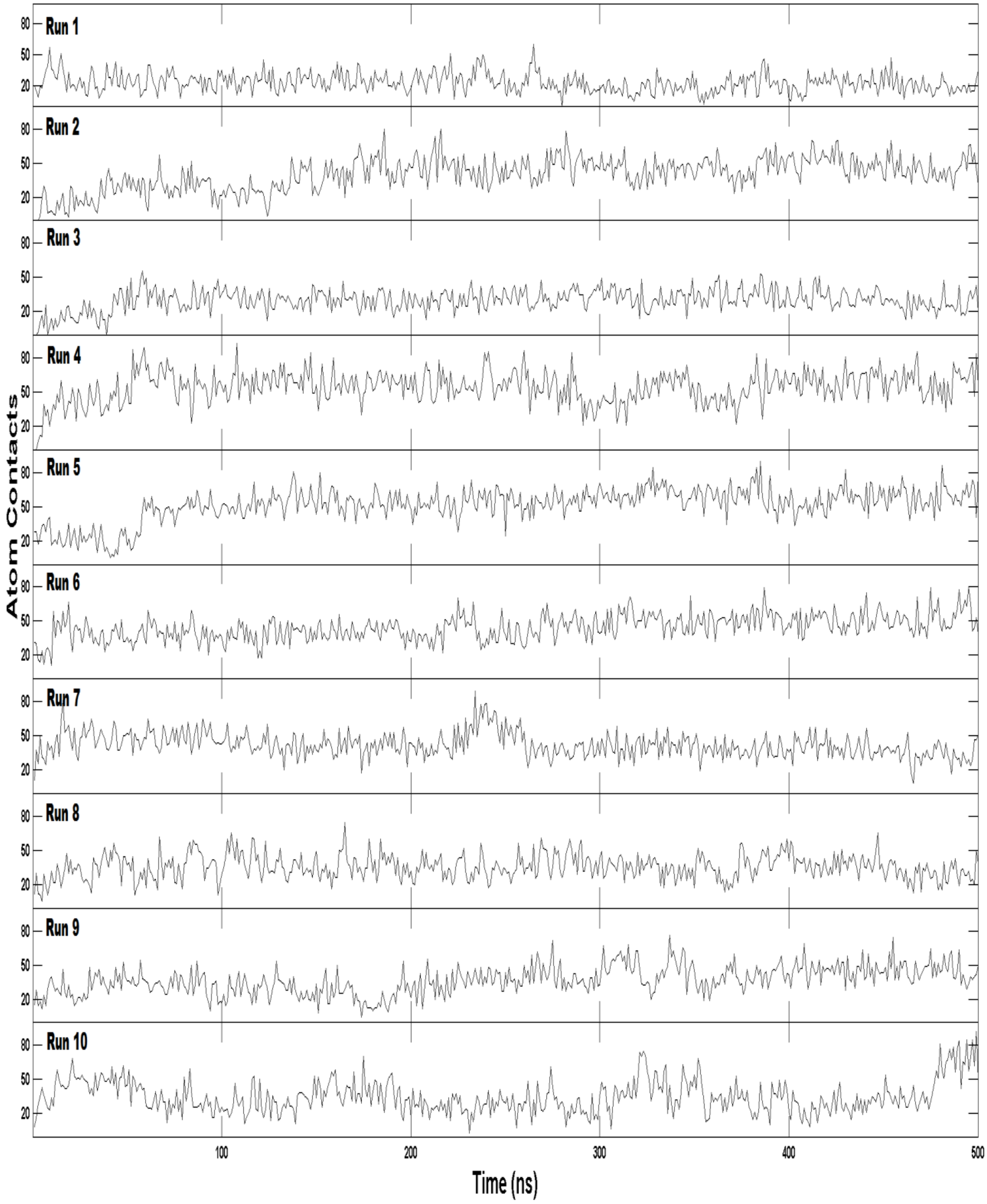

Figure S9. The contact number between the hybrid topological fold of DNA and BRACO19 in each trajectory.

Front View	Description	Front View	Description
Run 01 (Groove)		Run 06 (Groove)	
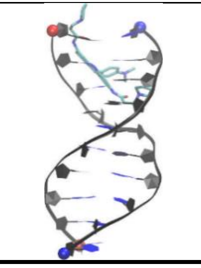	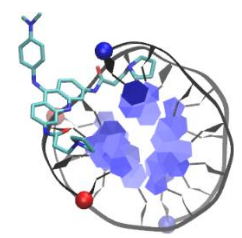	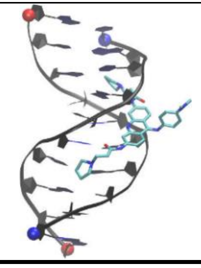	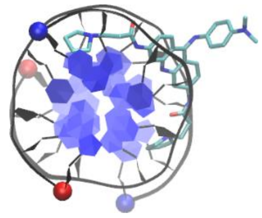
Run 02 (Groove)		Run 07 (Groove)	
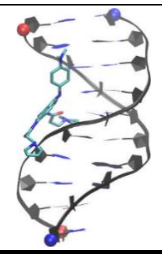	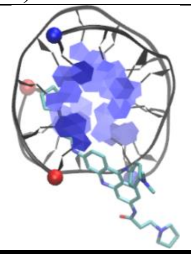	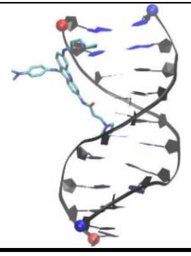	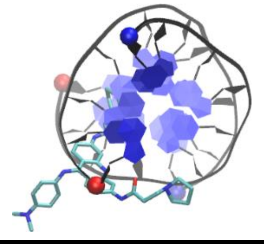
Run 03 (Groove)		Run 08 (Top)	
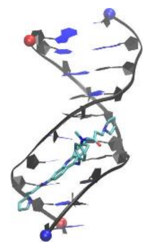	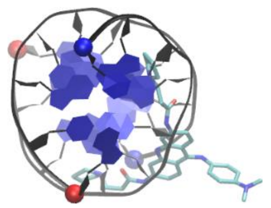	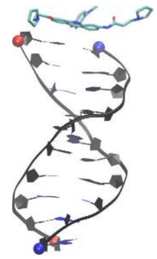	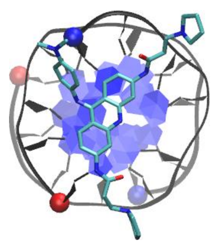
Run 04 (Bottom)		Run 09 (Groove)	
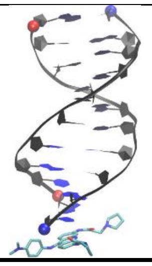	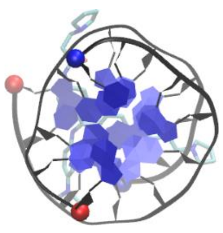	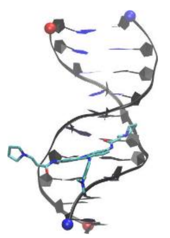	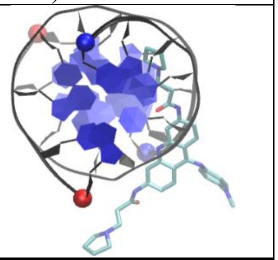
Run 05 (Groove)		Run 10 (Groove)	
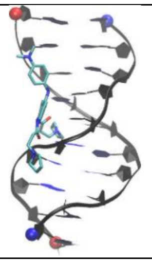	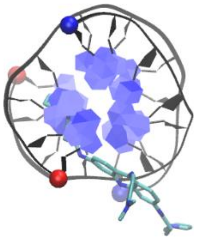	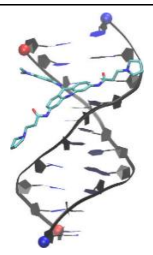	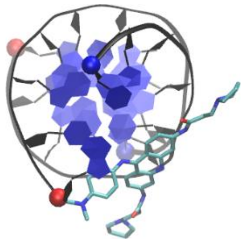

Figure S10. Last snapshots of the 10 BRACO19-duplex DNA complex simulations. 5’ and 3’ arc indicated by a red and blue ball, respectively.

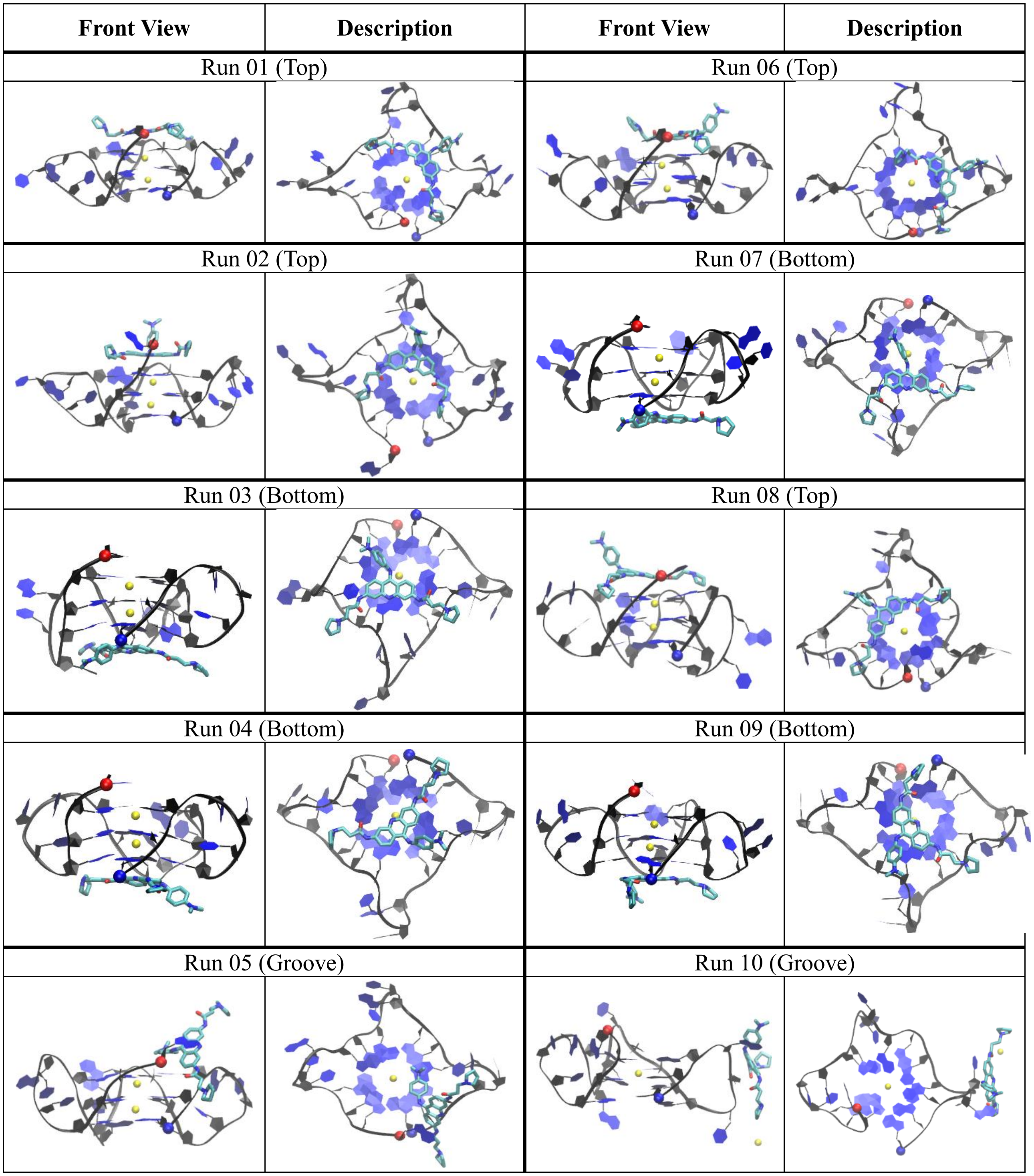

Figure S11. Last snapshots of the 10 3(-p-p-p)-BRACO19 complex simulations. 5' and 3' arc indicated by a red and blue ball, respectively.

Front View	Description	Front View	Description
Run 01 (Bottom Groove)		Run 06 (Top groove)	
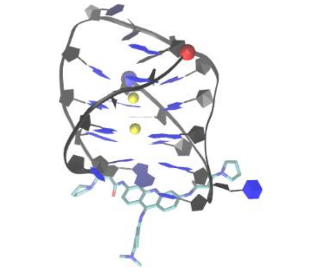	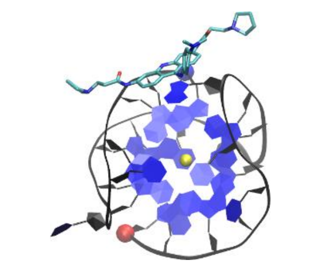	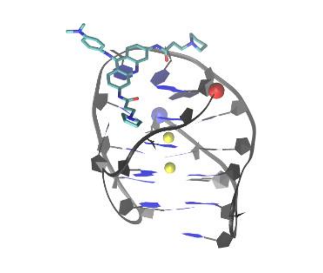	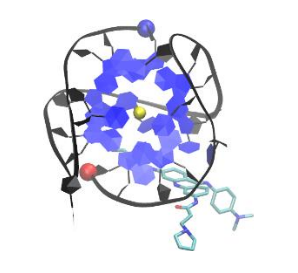
Run 02 (Bottom)		Run 07 (Bottom)	
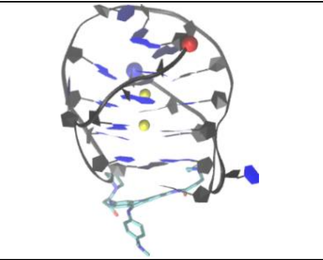	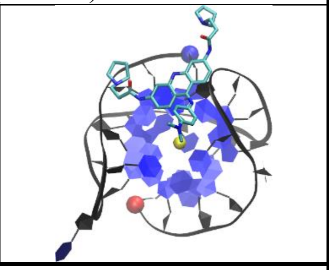	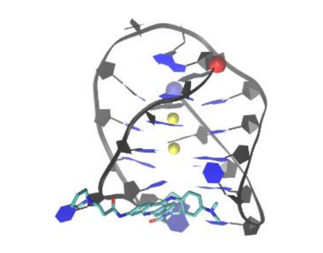	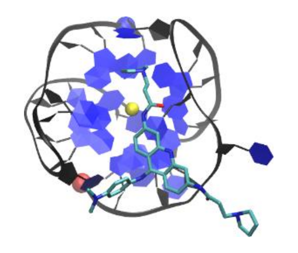
Run 03 (Groove)		Run 08(Top groove)	
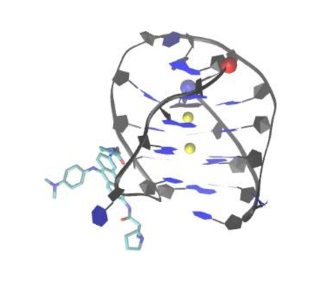	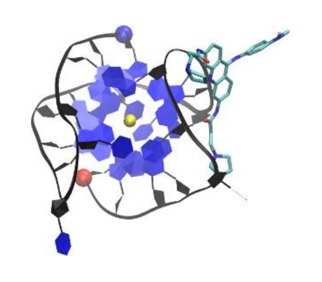	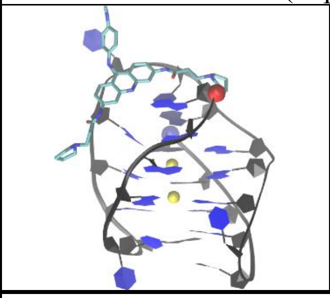	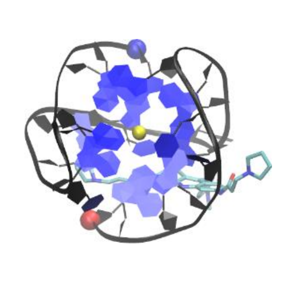
Run 04 (Bottom)		Run 09 (Bottom)	
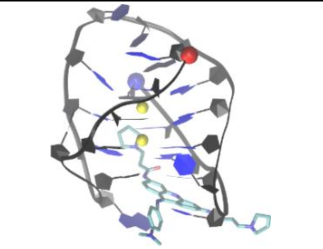	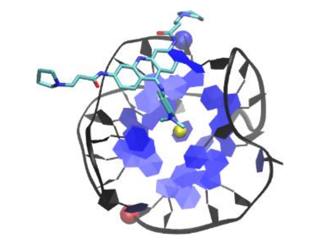	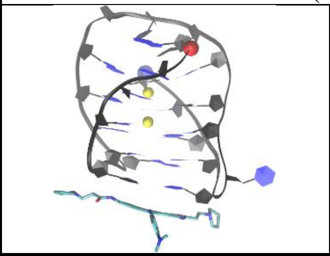	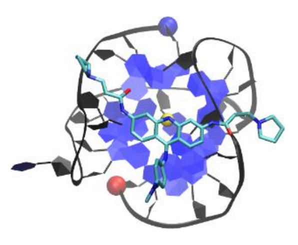
Run 05 (Top groove)		Run 10 (Bottom)	
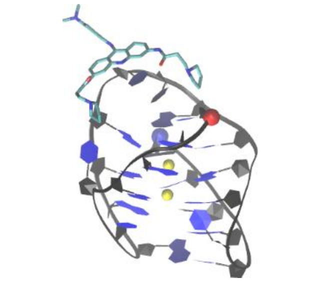	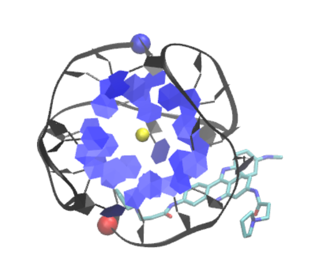	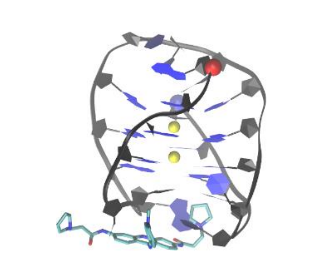	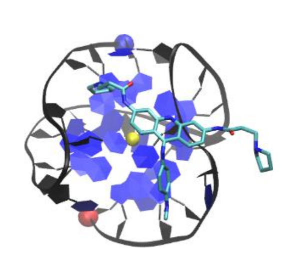

Figure S12. Last snapshots of the 10 3(-lwd+ln)-BRACO19 complex simulations. 5' and 3' arc indicated by a red and blue ball, respectively.

Front View	Description	Front View	Description
Run 01 (Top)		Run 06 (Bottom)	
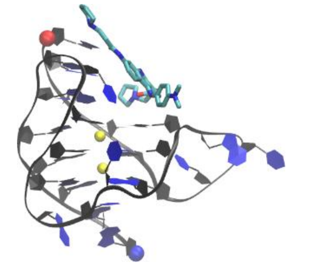	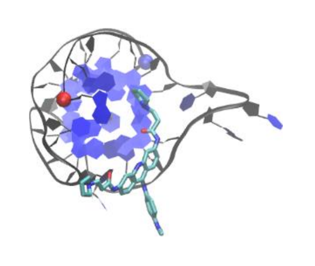	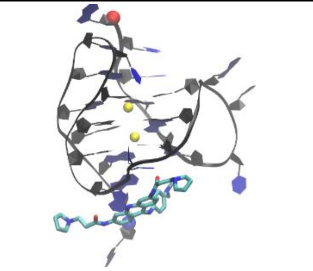	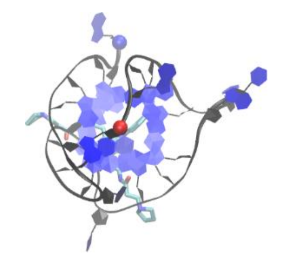
Run 02 (Bottom)		Run 07 (Top)	
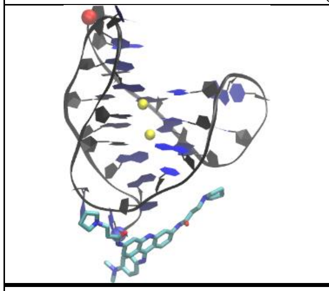	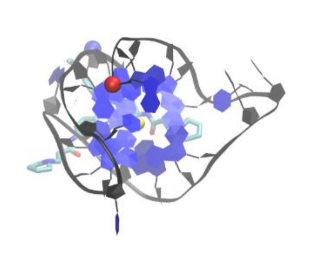	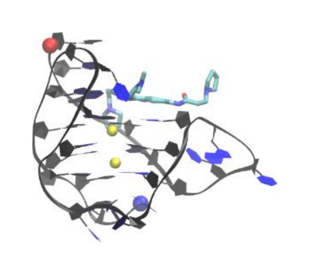	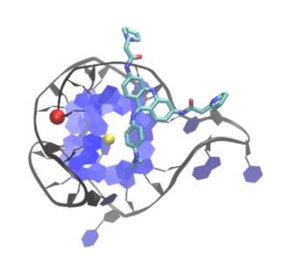
Run 03 (Groove)		Run 08 (Groove)	
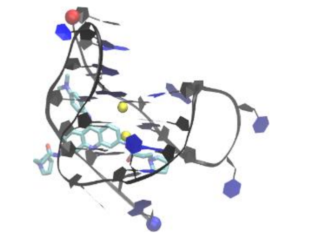	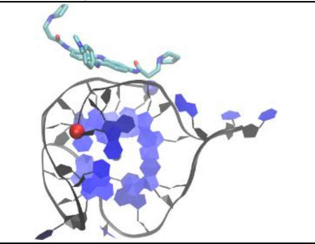	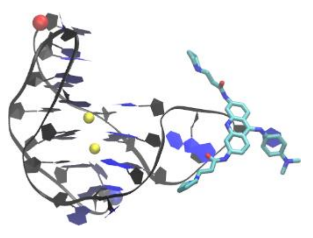	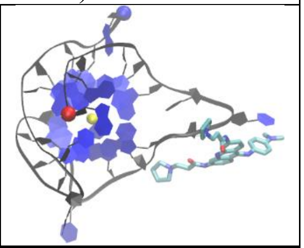
Run 04 (Groove)		Run 09 (Top)	
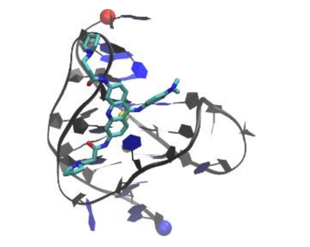	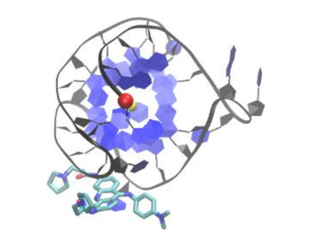	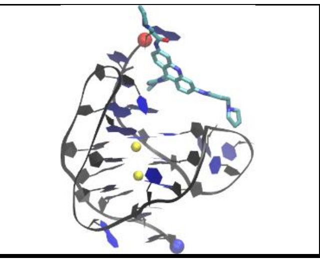	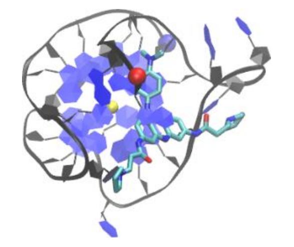
Run 05 (Groove)		Run 10 (Groove)	
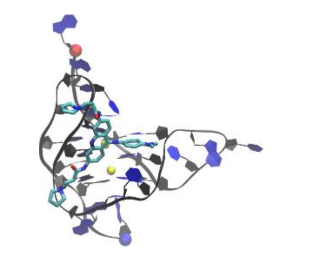	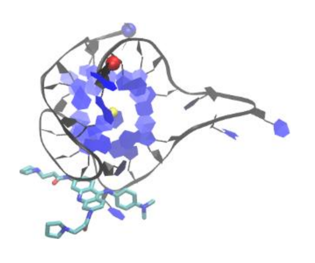	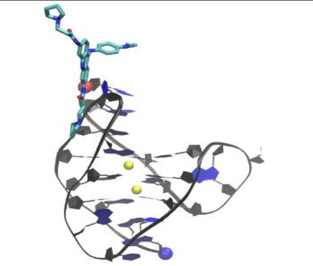	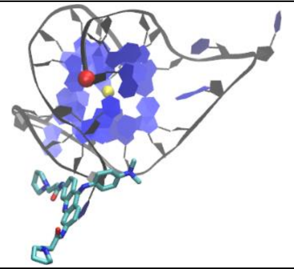

Figure S13. Last snapshots of 10 hybrid BRACO19 complex simulations. 5' and 3' arc indicated by a red and blue ball, respectively.

Binding model	Top Stacking	Groove Binding	Groove Binding
Cluster ID	A1	B1	B2
Representative Structure (Front View)	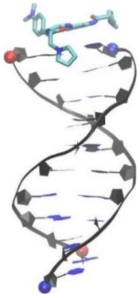	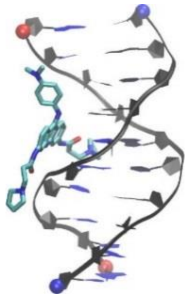	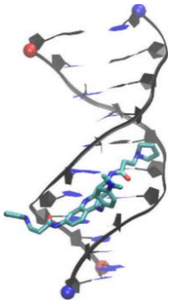
Representative Structure (Top/Bottom View)	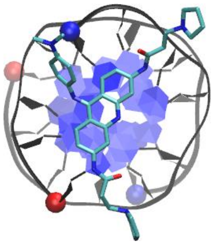	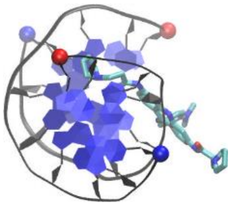	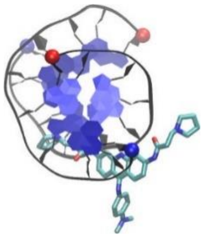
Population	4%	23%	19%
Binding Model	Groove Binding	Groove Binding	Groove Binding
Cluster ID	B3	B4	B5
Representative Structure (Front View)	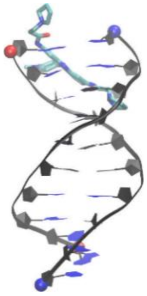	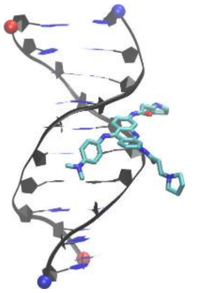	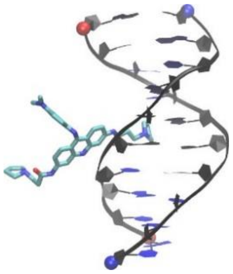
Representative Structure (Top/Bottom View)	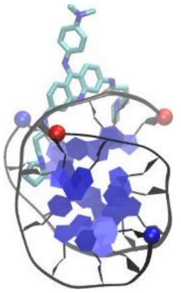	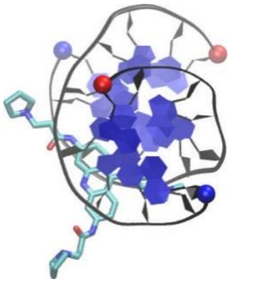	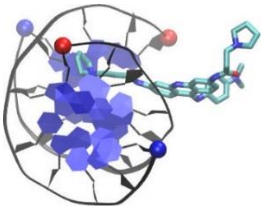
Population	10%	6%	5%

Binding Model	Groove Binding	Groove Binding	Groove Binding
Cluster ID	B6	B7	B8
Representative Structure (Front View)	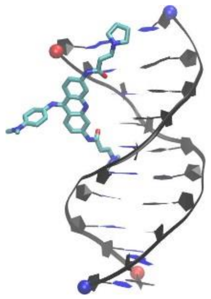	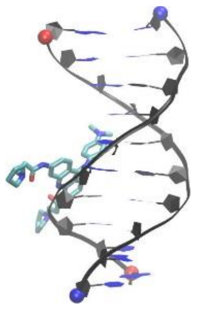	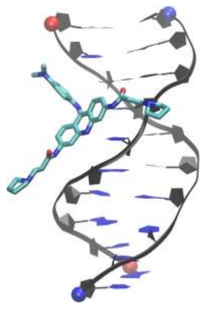
Representative Structure (Top/Bottom View)	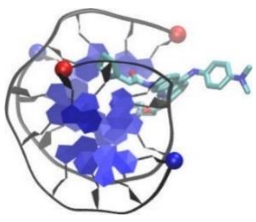	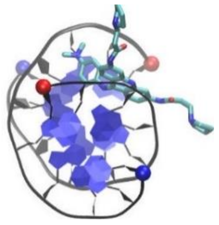	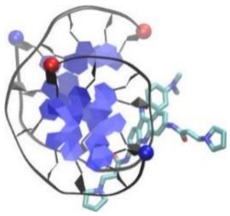
Population	4%	3%	3%
Binding Model	Groove Binding	Groove Binding	Groove Binding
Cluster ID	B9	B10	B11
Representative Structure (Front View)	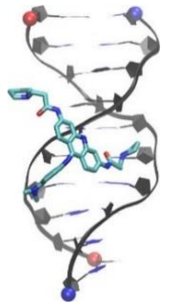	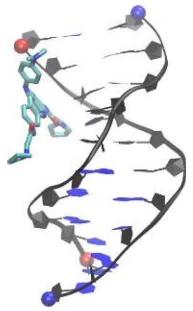	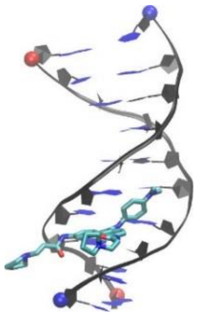
Representative Structure (Top/Bottom View)	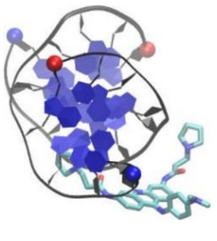	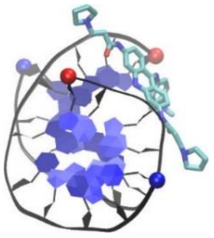	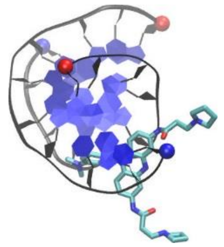
Population	2%	2%	2%

Binding model	Groove Binding	Bottom Binding	
Cluster ID	B12	C1	
Representative Structure (Front View)	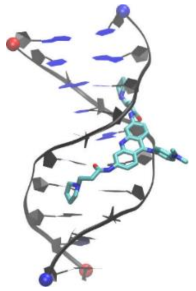	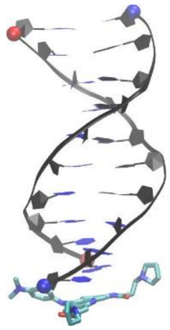	
Representative Structure (Top/Bottom View)	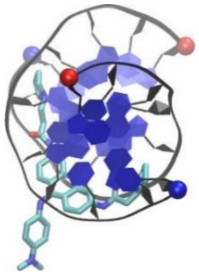	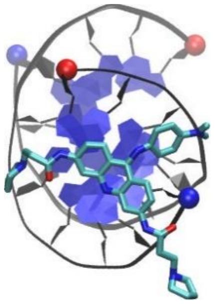	
Population	2%	2%	

Figure S14. Representative structures of the most populated complex structure families (population ≥ 1 %) from the clustering analysis of the combined binding trajectories. 5’ and 3’ are indicated by a red and blue ball, respectively for the DNA duplex-BRACO19 complex system.

Binding model	Top Stacking	Top Stacking	Top Stacking
Cluster ID	A1	A2	A3
Representative Structure (Front View)	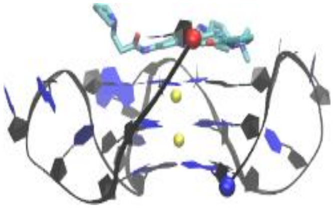	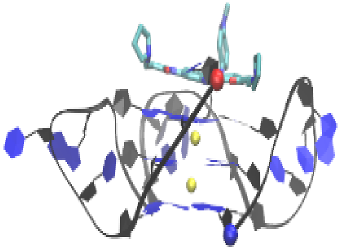	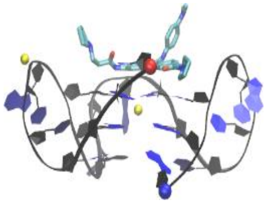
Representative Structure (Top/Bottom View)	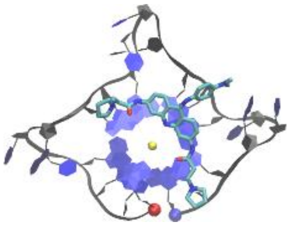	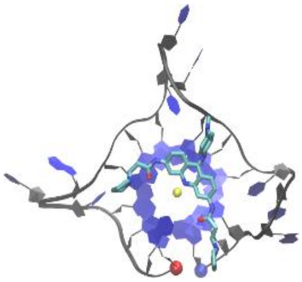	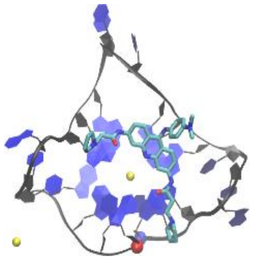
Population	16%	5%	4%
Binding Model	Top Stacking	Bottom Stacking	Bottom Stacking
Cluster ID	A4	B1	B2
Representative Structure (Front View)	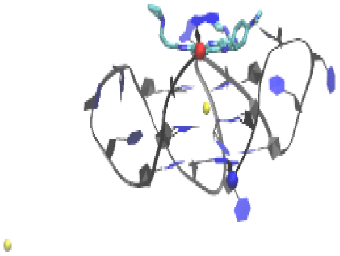	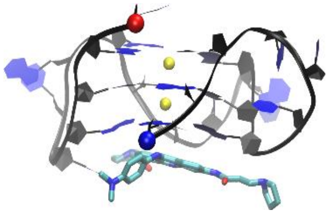	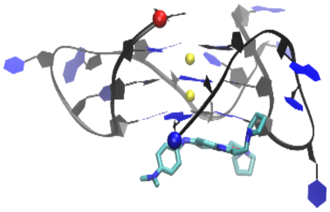
Representative Structure (Top/Bottom View)	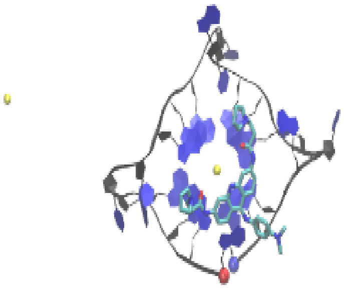	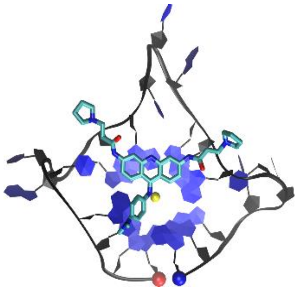	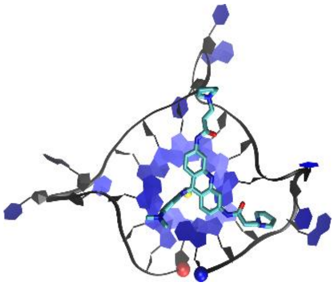
Population	3%	31%	10%

Binding Model	Groove Binding	Groove Binding	Groove Binding
Cluster ID	C1	C2	C3
Representative Structure (Front View)	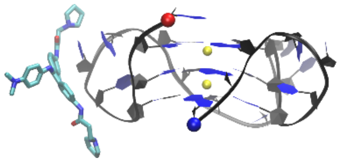	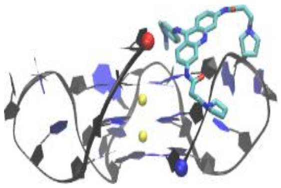	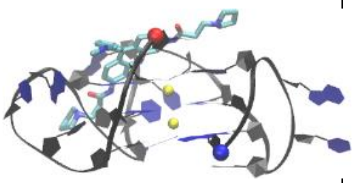
Representative Structure (Top/Bottom View)	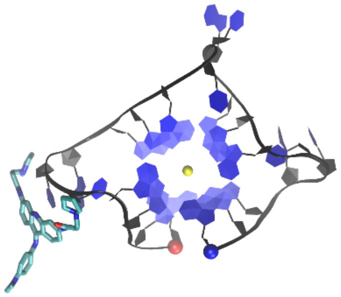	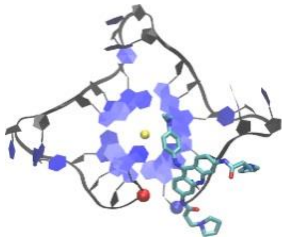	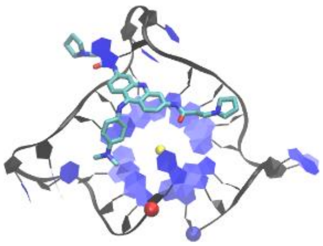
Population	9%	8%	7%
Binding Model	Groove Binding	Groove Binding	
Cluster ID	C4	B5	
Representative Structure (Front View)	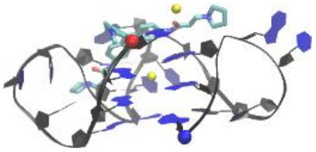	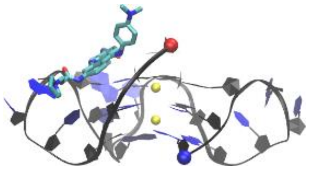	
Representative Structure (Top/Bottom View)	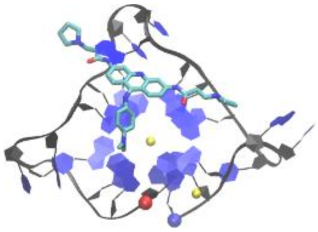	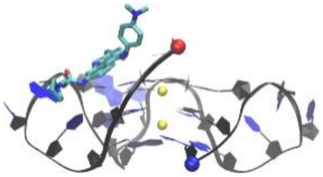	
Population	3%	2%	

Figure S15. Representative structures of the most populated complex structure families (population ≥ 1 %) from the clustering analysis of the combined binding trajectories. 5’ and 3’ are indicated by a red and blue ball, respectively for the 3(-p-p-p)-BRACO19 complex system.

Binding model	Top Stacking	Top Stacking	Top Stacking
Cluster ID	A1	A2	A3
Representative Structure (Front View)	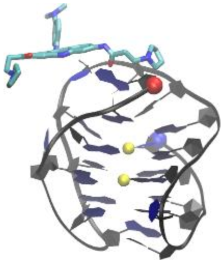	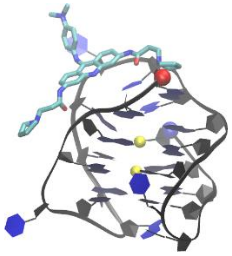	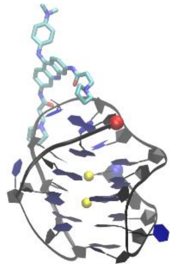
Representative Structure (Top/Bottom View)	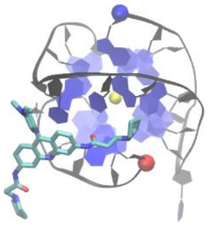	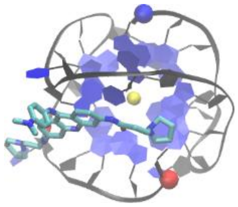	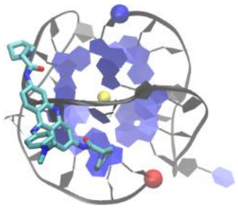
Population	19%	4%	2%
Binding Model	Groove Binding	Groove Binding	Groove Binding
Cluster ID	B1	B2	B4
Representative Structure (Front View)	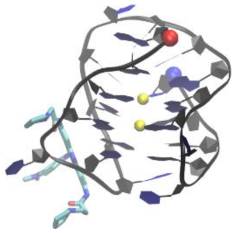	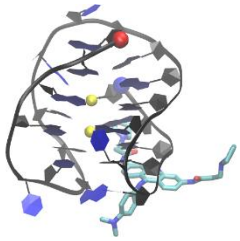	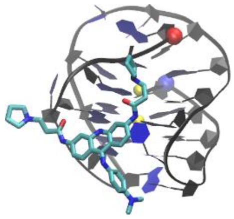
Representative Structure (Top/Bottom View)	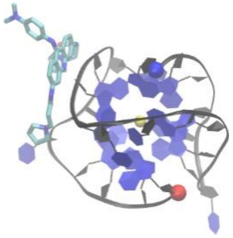	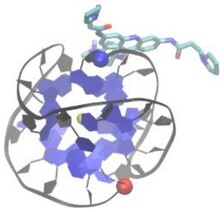	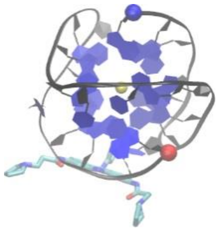
Population	15%	10%	3%
Binding Model	Groove Binding	Groove Binding	Bottom Binding
Cluster ID	B5	B6	C1

Representative Structure<br>(Front View)	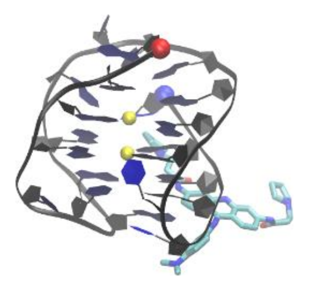	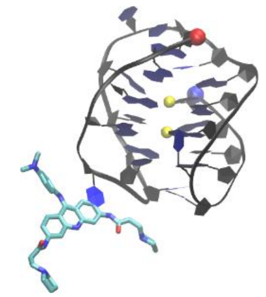	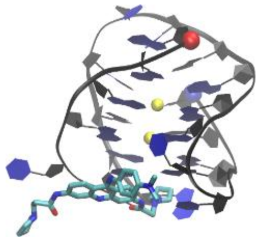
Representative Structure<br>(Top/Bottom View)	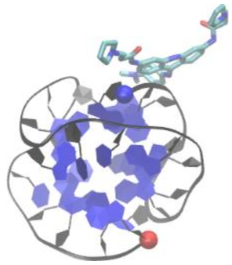	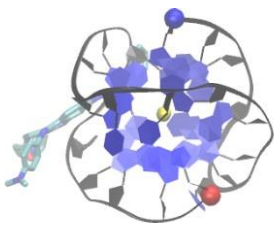	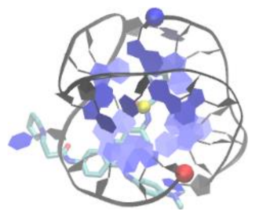
Population	1%	1%	45%

Figure S16. Representative structures of the most populated complex structure families (population ≥ 1 %) from the clustering analysis of the combined binding trajectories. 5' and 3' are indicated by a red and blue ball, respectively for the 3(-lwd+ln)-BRACO19 complex system.

Binding model	Top Stacking	Top Stacking	Top Stacking
Cluster ID	A1	A2	A3
Representative Structure (Front View)	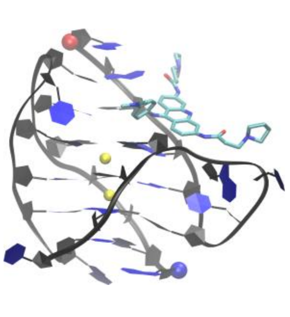	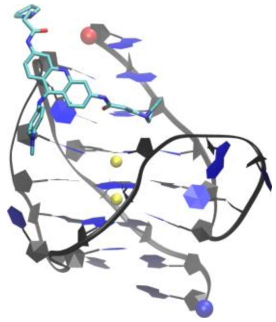	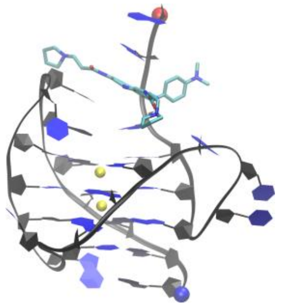
Representative Structure (Top/Bottom View)	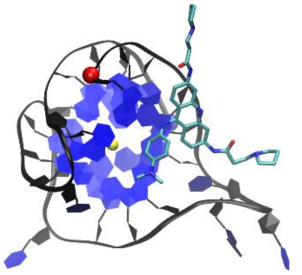	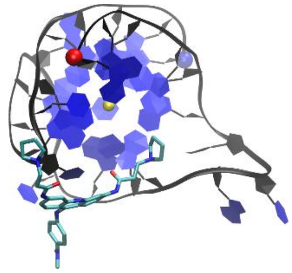	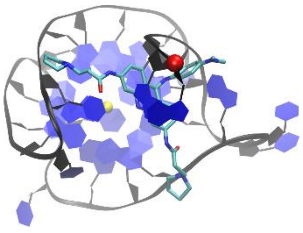
Population	12%	9%	7%
Binding Model	Top stacking	Groove Binding	Groove Binding
Cluster ID	A4	B1	B2
Representative Structure (Front View)	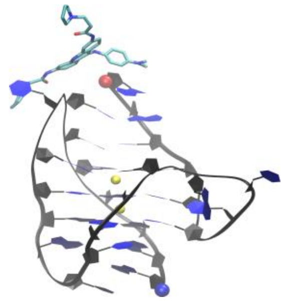	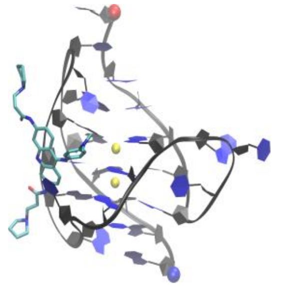	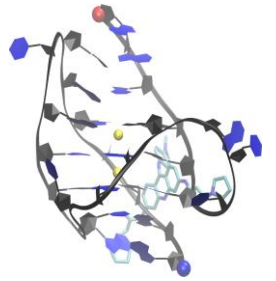
Representative Structure (Top/Bottom View)	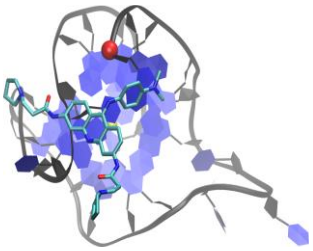	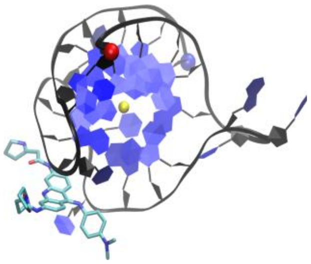	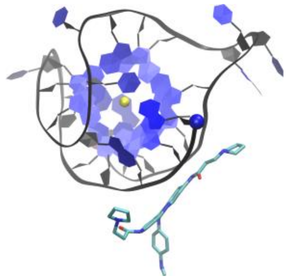
Population	5%	22%	10%

Binding Model	Groove Binding	Groove Binding	Bottom Binding
Cluster ID	B3	B4	C1
Representative Structure<br><br>(Front View)	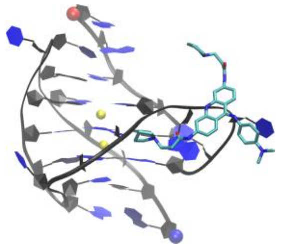	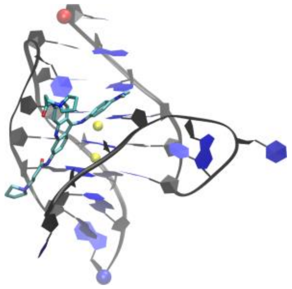	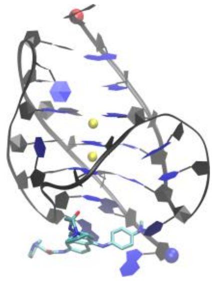
Representative Structure<br><br>(Top/Bottom View)	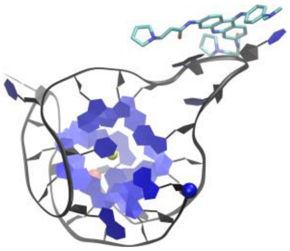	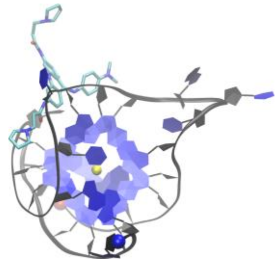	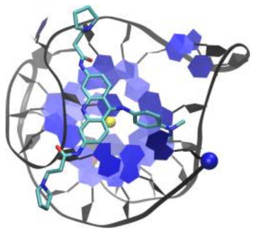
Population	9%	2%	10%
Binding Model	Bottom Binding	Bottom Binding	
Cluster ID	C2	C3	
Representative Structure<br><br>(Front View)	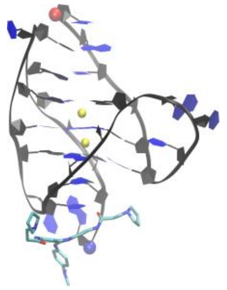	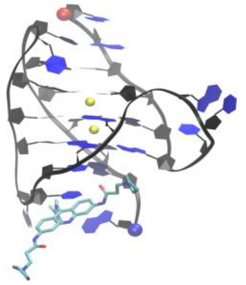	
Representative Structure<br><br>(Top/Bottom View)	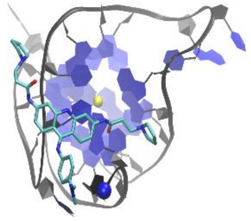	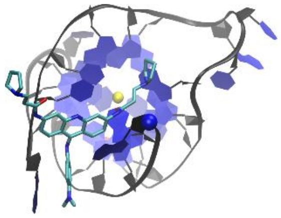	
Population	9%	1%	

Figure S17. Representative structures of the most populated complex structure families (population ≥ 1 %) from the clustering analysis of the combined binding trajectories. 5' and 3' are indicated by a red and blue ball, respectively for the 3(-p-lw-ln)-BRACO19 complex system.

<table>
<tr><th></th><th>2D interactions</th><th>2D interactions with expanded interacting residues</th></tr>
<tr><td>(A) Groove Binding Mode of Duplex</td><td>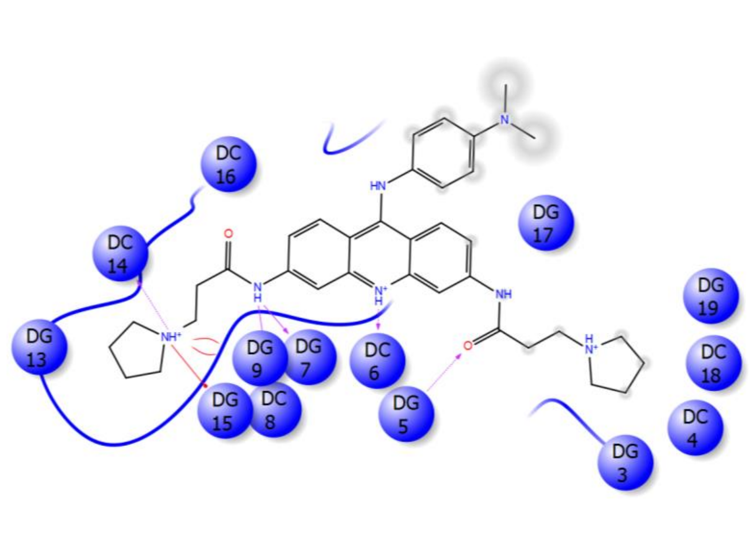
</td><td>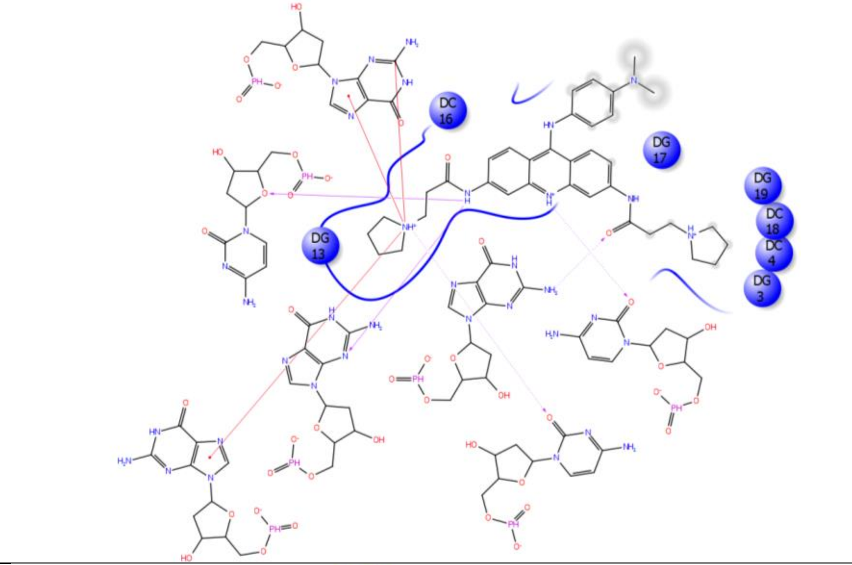
</td></tr>
<tr><td>(B) Top Stacking mode of parallel G-Quadruplex</td><td>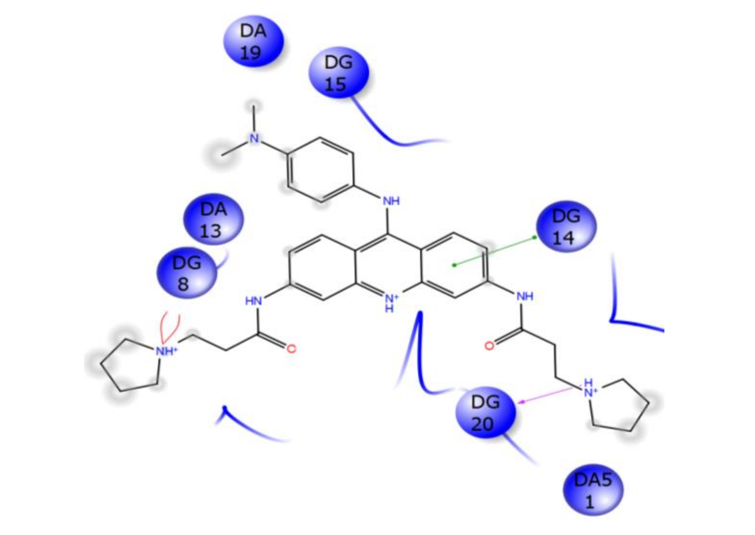
</td><td>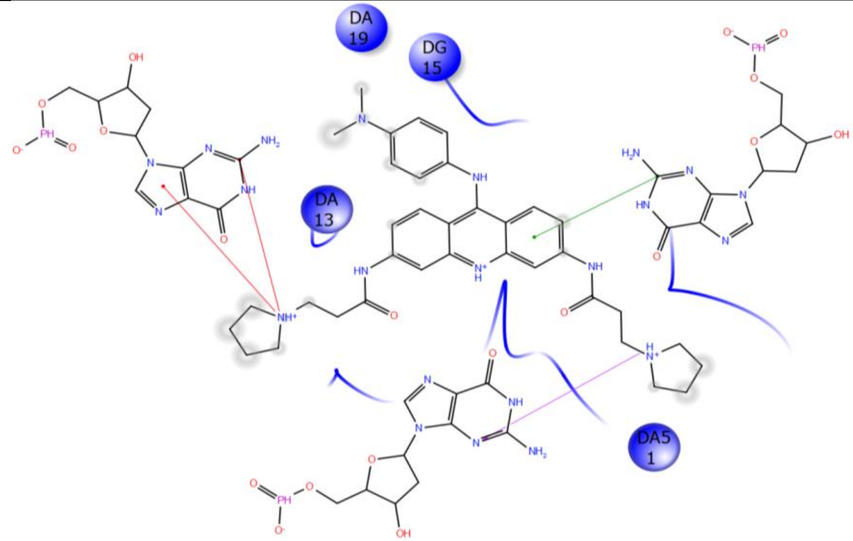
</td></tr>
</table>

(C) Groove Binding Mode of 3(-p-p-p) G-Quadruplex

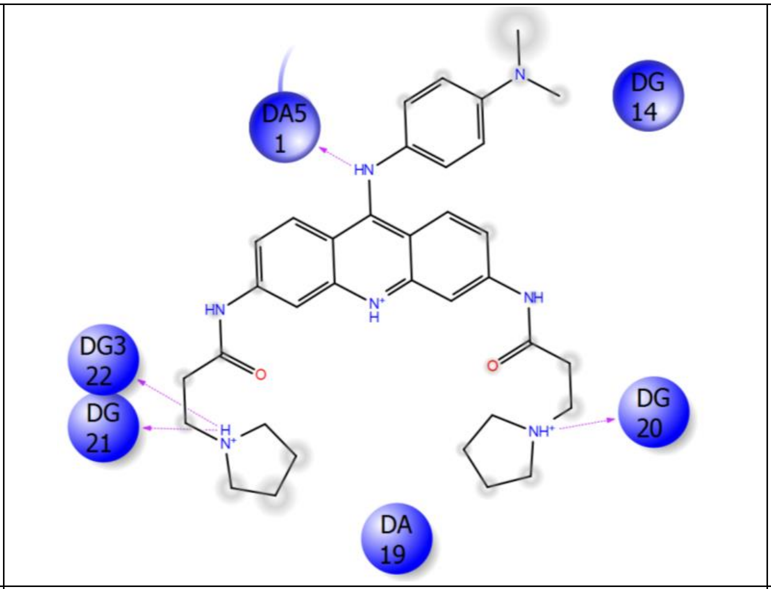

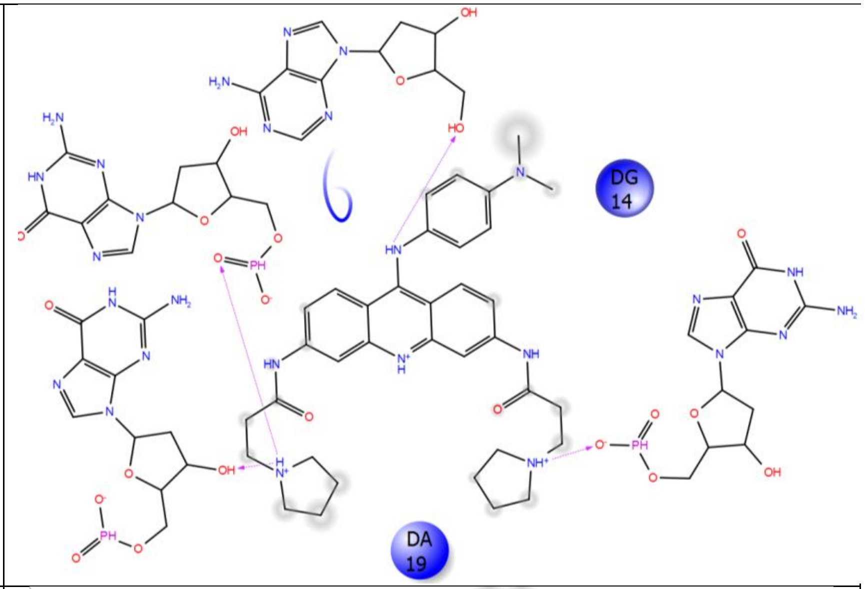

(D) Top Stacking Mode of anti-parallel G-Quadruplex

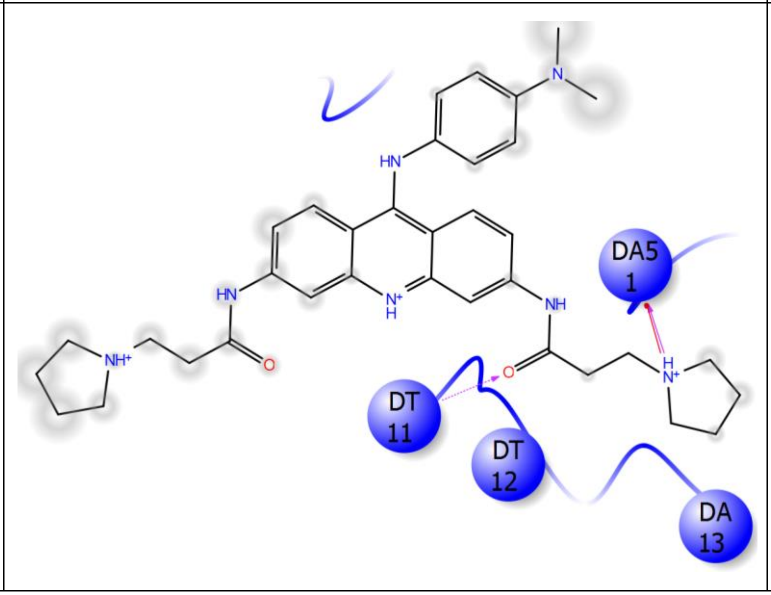

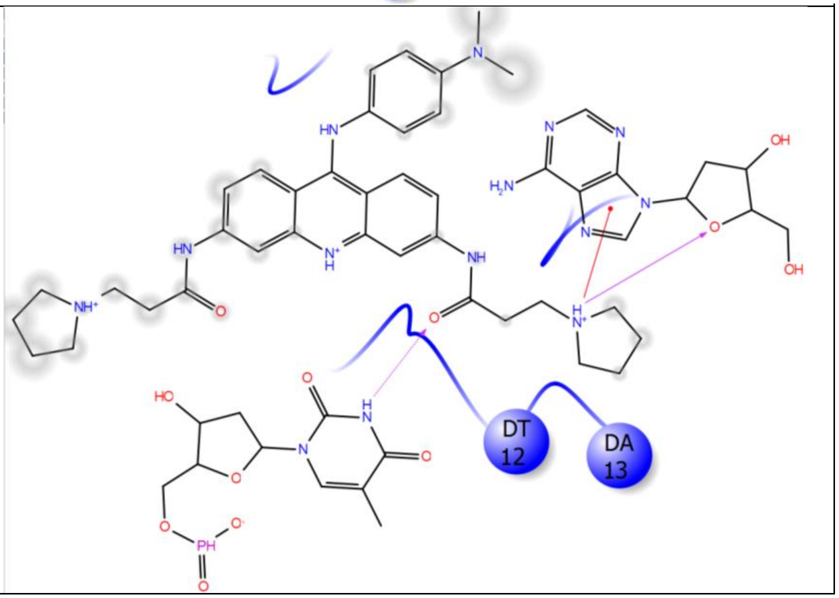

(E) Groove Binding Mode of anti-parallel G-Quadruplex

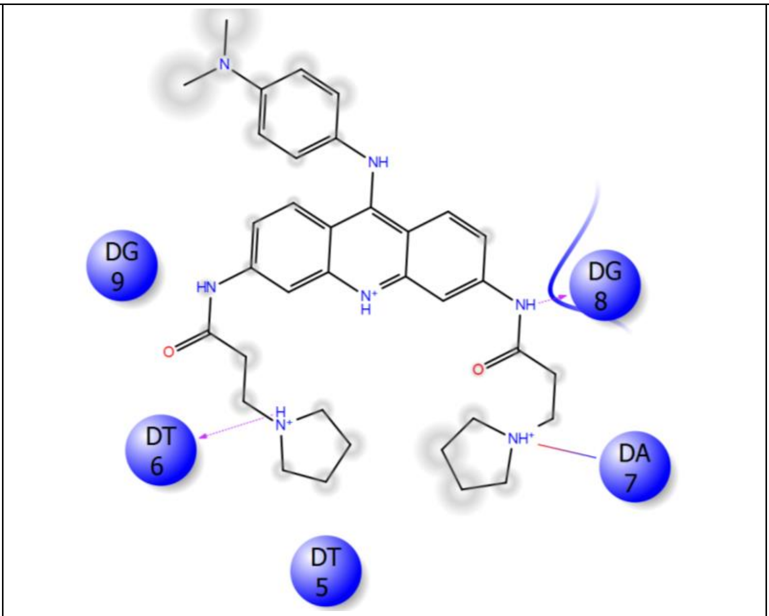

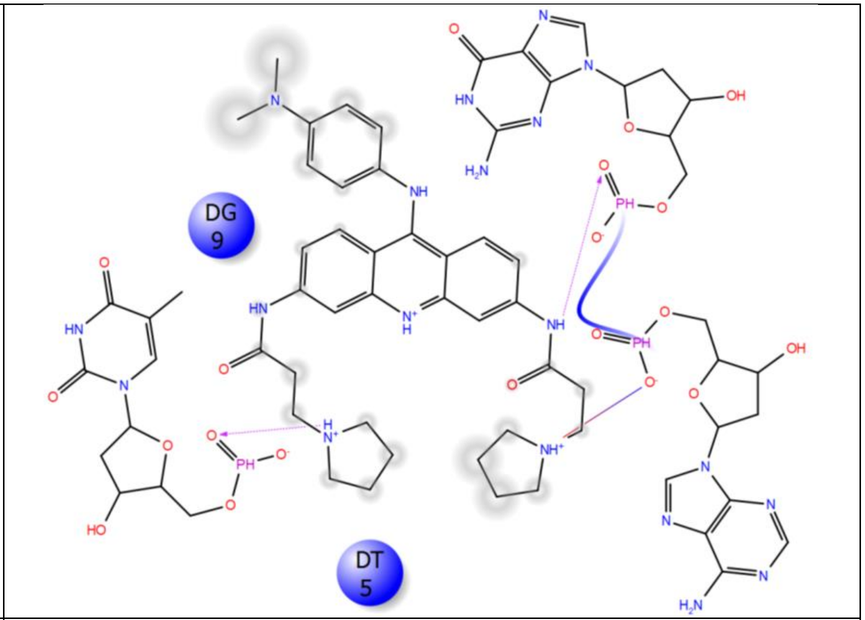

(F) Bottom Stacking Mode of anti-parallel G-Quadruplex

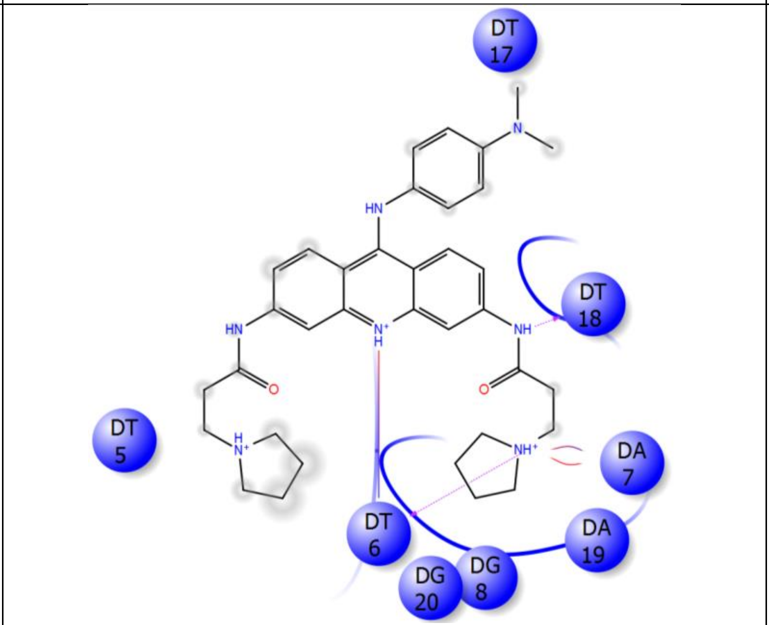

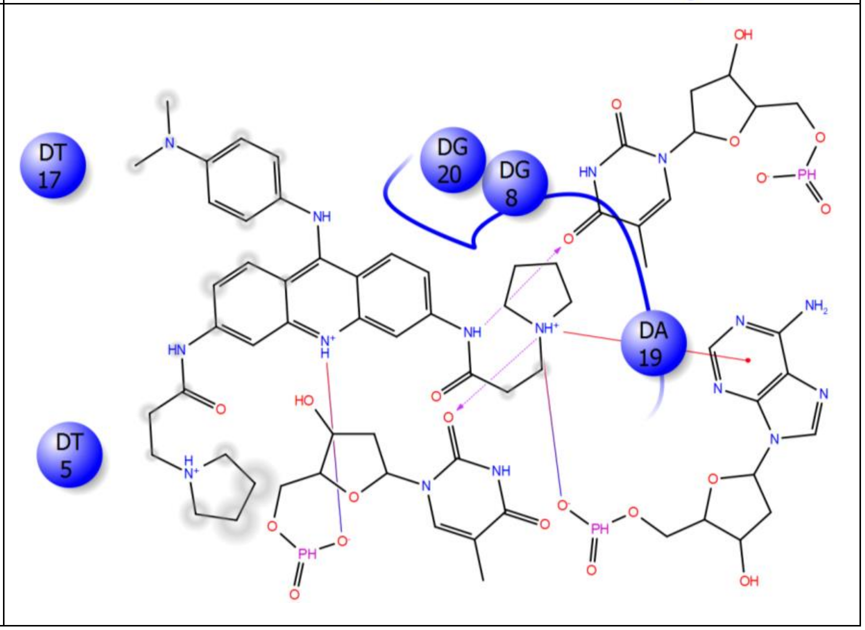

<table>
<tr>
<td>(G) Top Stacking mode of hybrid G-Quadruplex</td>
<td>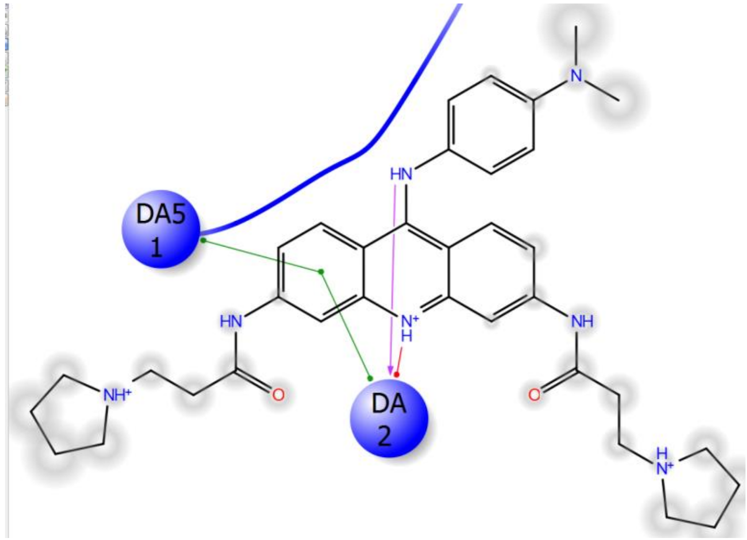
</td>
<td>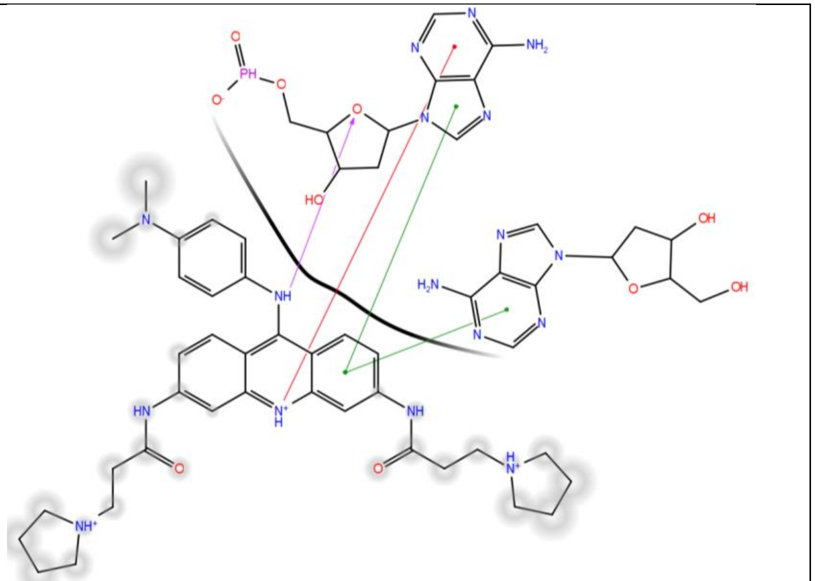
</td>
</tr>
<tr>
<td>(H) Groove Binding Mode of anti-parallel G-Quadruplex</td>
<td>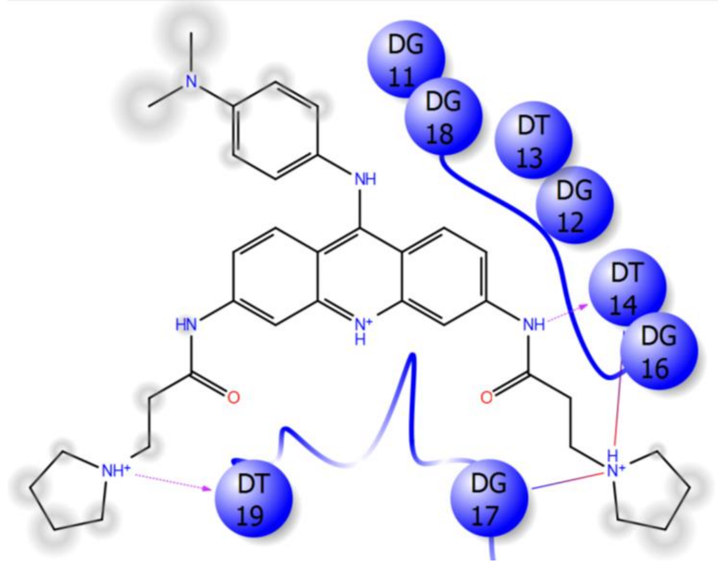
</td>
<td>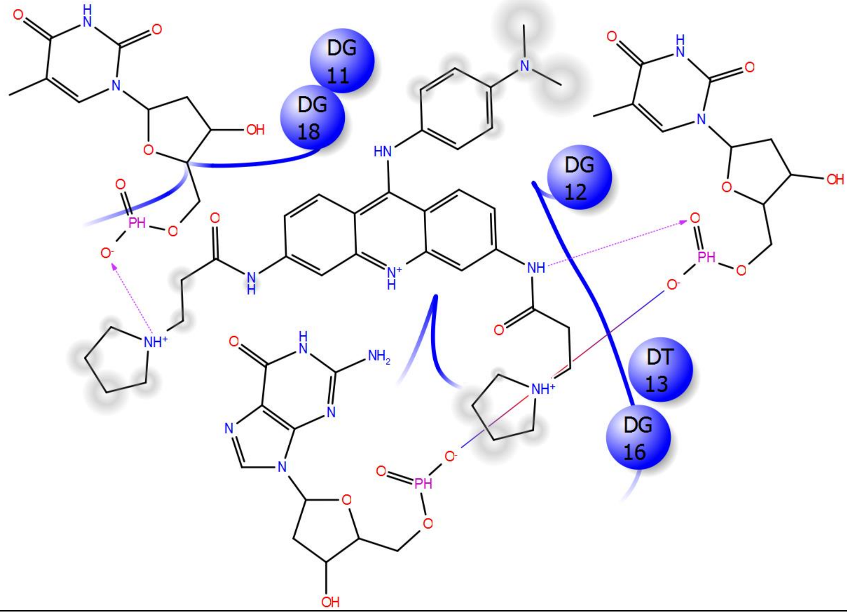
</td>
</tr>
</table>

(I) Bottom Stacking Mode of hybrid G-Quadruplex

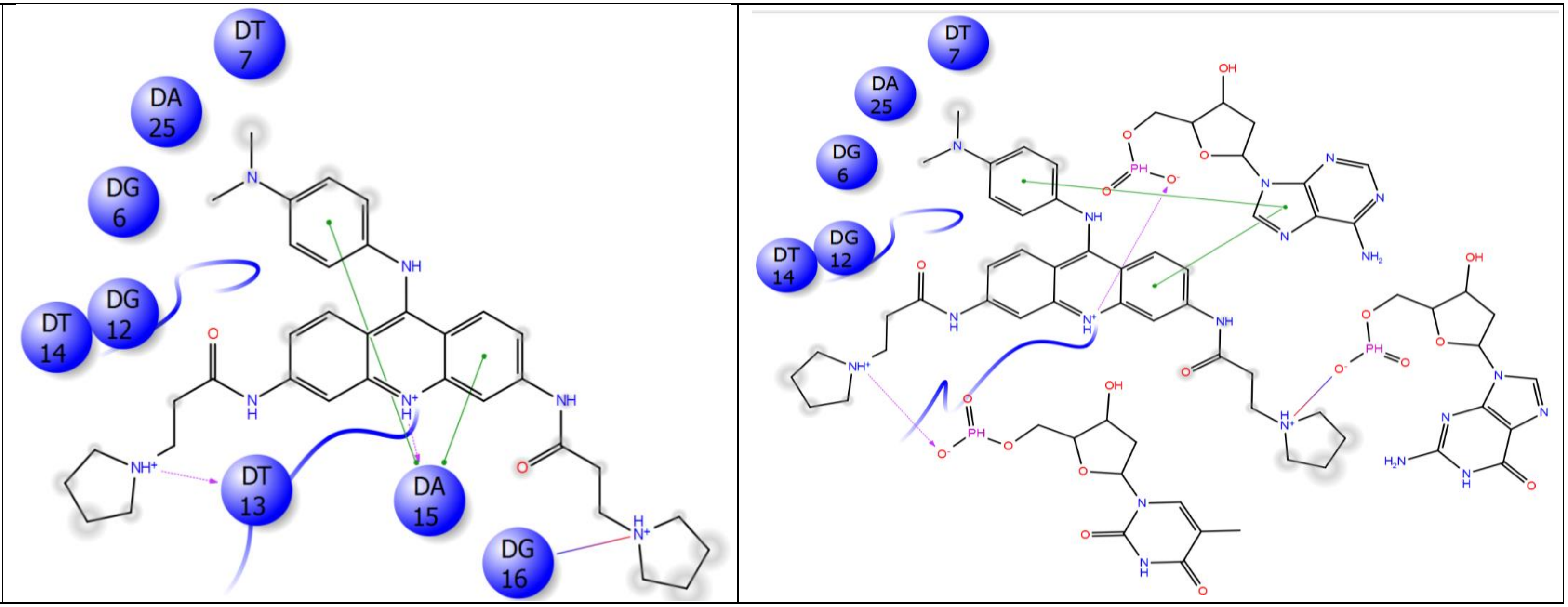

Figure S18. 2D interactions of BRACO19 in representative structures of (A) Groove Binding Mode of the Duplex DNA, (B) Top Stacking mode of the parallel G-Quadruplex, (C) Groove Binding Mode of the parallel G-Quadruplex, (D) Top Stacking mode of the anti-parallel G-Quadruplex, (E) Groove Binding Mode of the anti-parallel G-Quadruplex, (F) Bottom Stacking Mode of the anti-parallel G-Quadruplex, (G) Top Stacking mode of the hybrid G-Quadruplex, (H) Groove Binding Mode of the anti-parallel G-Quadruplex and (I) Bottom Stacking Mode of the hybrid G-Quadruplex.

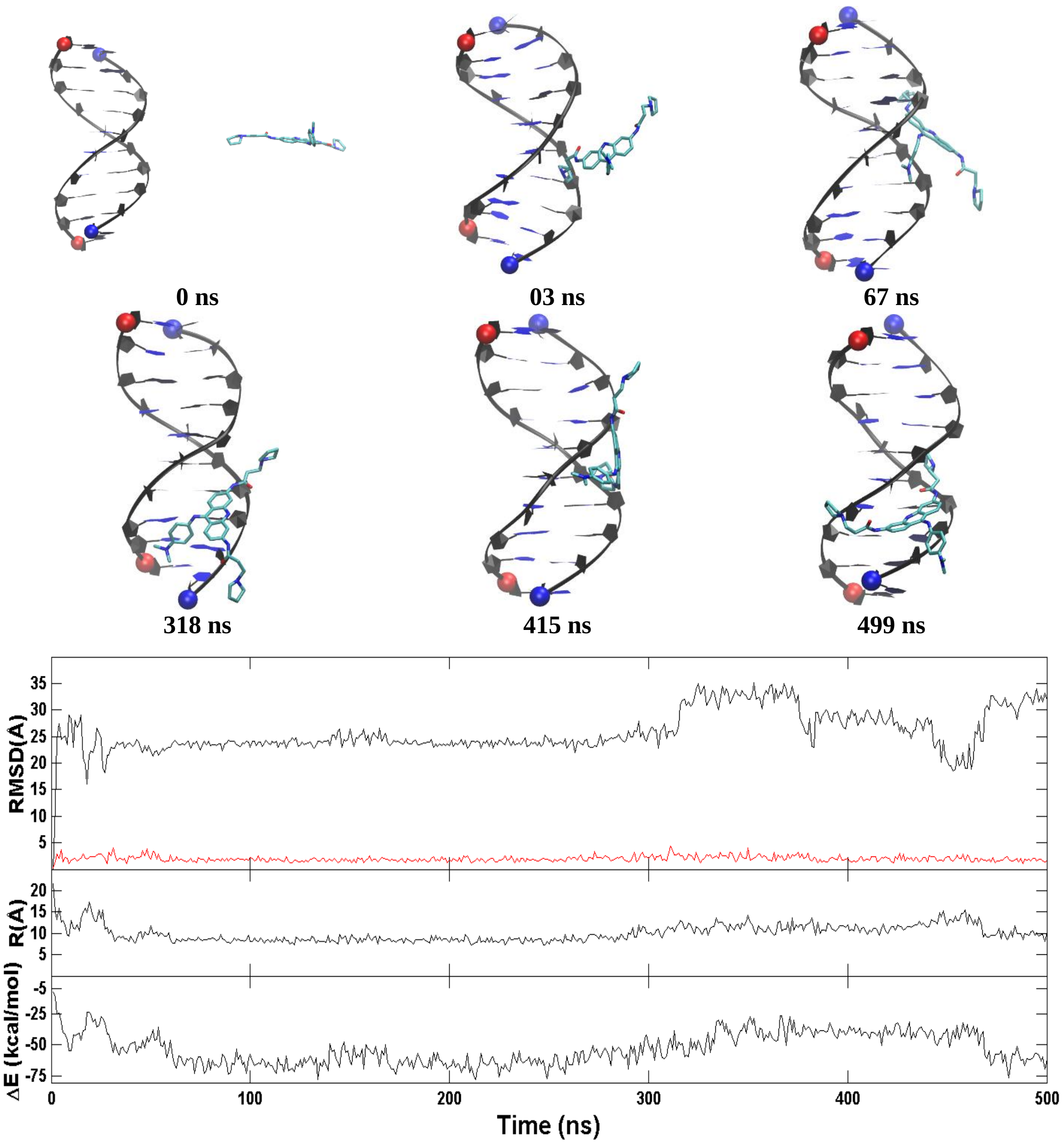

Figure S19. Another representative groove binding trajectory of the duplex DNA. **Top:** Representative structures with time annotation. 5' and 3' are indicated by a red and blue ball, respectively. **Bottom:** An order parameter plot depicting number of hydrogen bonds present in first base pair (green), second base pair (red) and third base pair (blue) tetrads of the DNA structure (figure 2), the drug-base dihedral angle, receptor (red) and ligand (black) RMSD relative to the original crystal pose, center-to-center distance and MM-GBSA binding energy (ΔE) (cf. methods section for definition).

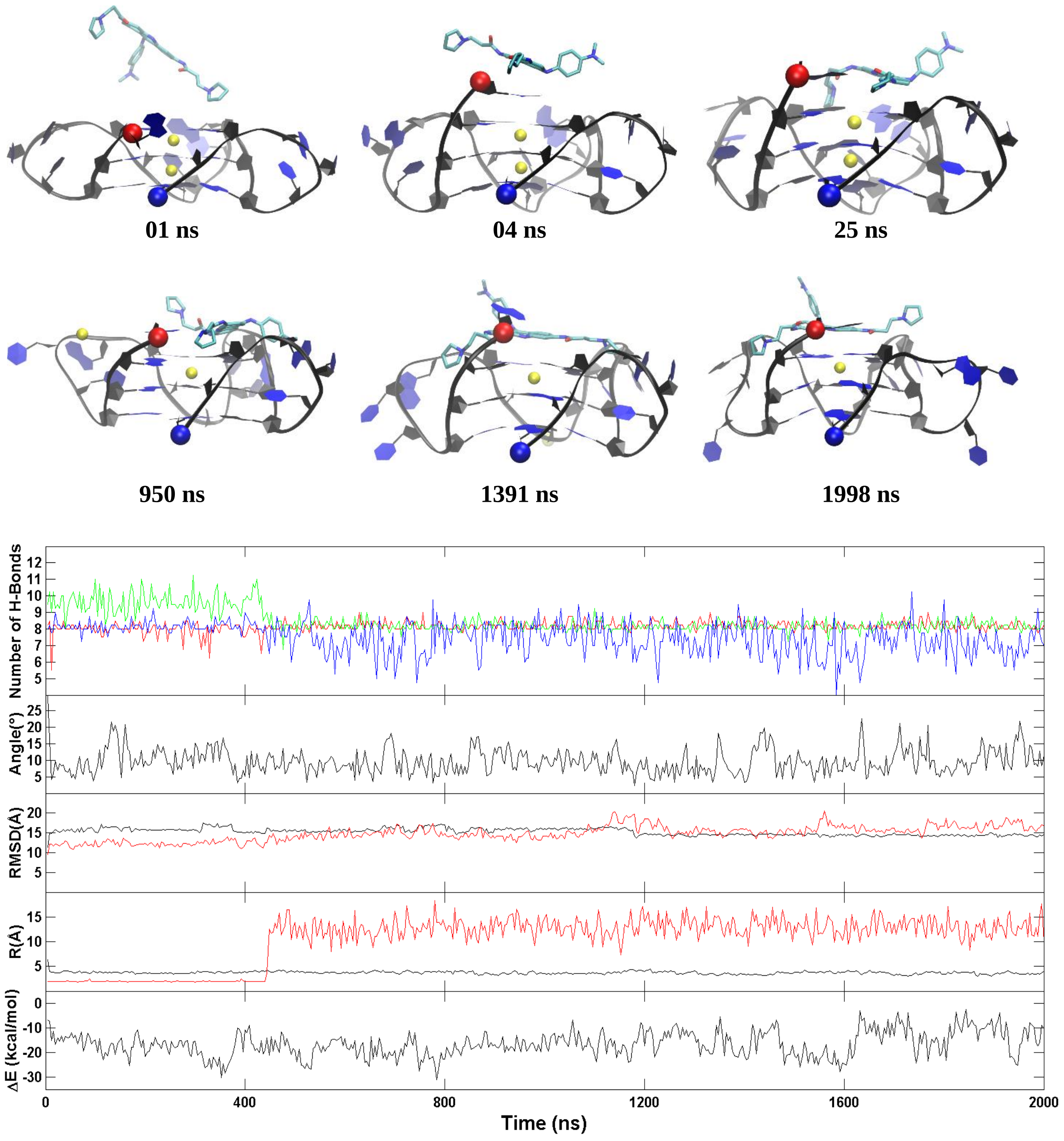

Figure S20. Another representative top stacking trajectory of the parallel G-quadruplex. **Top:** Representative structures with time annotation. 5' and 3' are indicated by a red and blue ball, respectively. Residues 1, 2, 13, 14 are indicated in purple and residues 12, 24 are indicated in red and the K^+ ions are represented in yellow. **Bottom:** An order parameter plot depicting number of hydrogen bonds present in first G4 (green), second G4 (red) and third G4 (blue) tetrads of the DNA structure (Figure 2), the drug-base dihedral angle, receptor (red) and ligand (black) RMSD relative to the original crystal pose, center-to-center distance (R/black) and K^+-K^+ distance (R/red) and MM-GBSA binding energy (ΔE) (cf. methods section for definition).

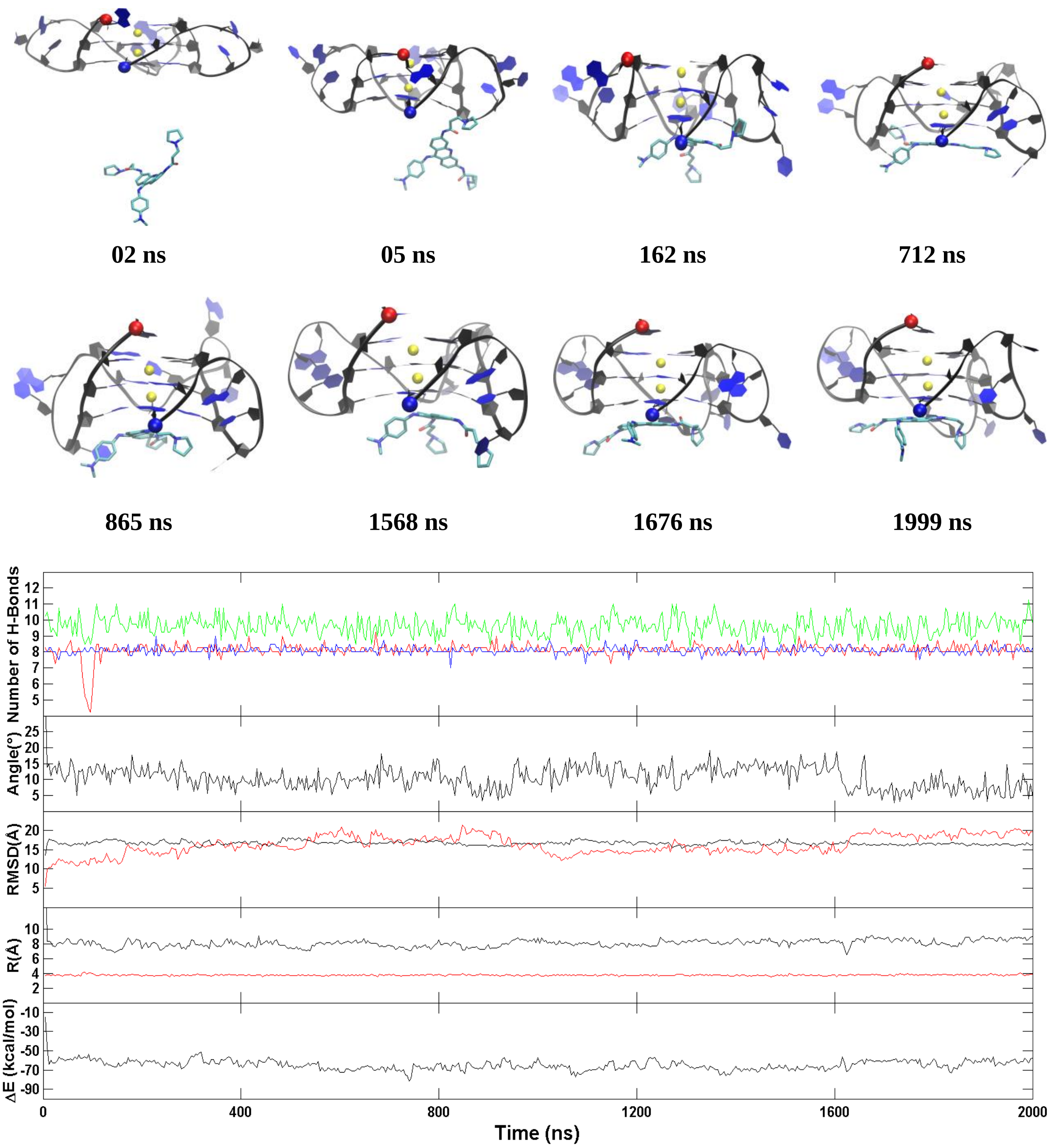

Figure S21. A representative bottom binding trajectory of the parallel G-quadruplex. **Top:** Representative structures with time annotation. 5' and 3' are indicated by a red and blue ball, respectively. Residues 1, 2, 13, 14 are indicated in purple and residues 12, 24 are indicated in red and the K^+ ions are represented in yellow. **Bottom:** An order parameter plot depicting number of hydrogen bonds present in first G4 (green), second G4 (red) and third G4 (blue) tetrads of the DNA structure (Figure 2), the drug-base dihedral angle, receptor (red) and ligand (black) RMSD relative to the original crystal pose, center-to-center distance (R/black) and K^+-K^+ distance (R/red) and MM-GBSA binding energy (ΔE) (cf. methods section for definition).

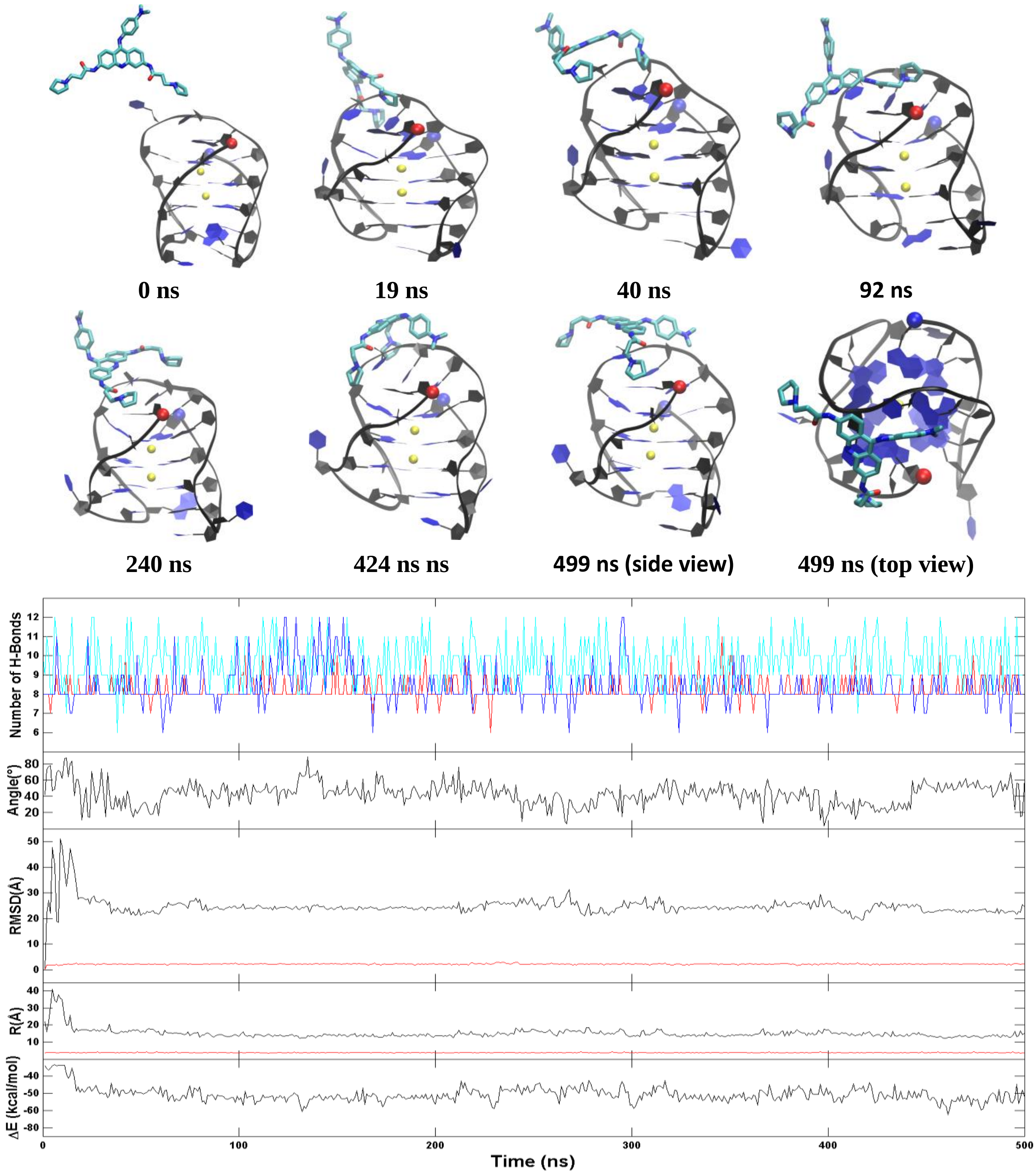

Figure S22. A representative top groove binding trajectory of the anti-parallel G-quadruplex. **Top:** Representative structures with time annotation. 5’ and 3’ are indicated by a red and blue ball, respectively. Residues 1, 2, 13, 14 are indicated in purple and residues 12, 24 are indicated in red and the K^+ ions are represented in yellow. **Bottom:** An order parameter plot depicting number of hydrogen bonds present in first (red), second G4 (cyan), third G4 (blue), fourth G4 (black) and fifth (green) layers of the DNA structure (Figure S9), the drug-base dihedral angle, receptor (red) and ligand (black) RMSD relative to the original crystal pose, center-to-center distance (R/black) and K^+-K^+ distance (R/red) and MM-GBSA binding energy (ΔE) (cf. methods section for definition).

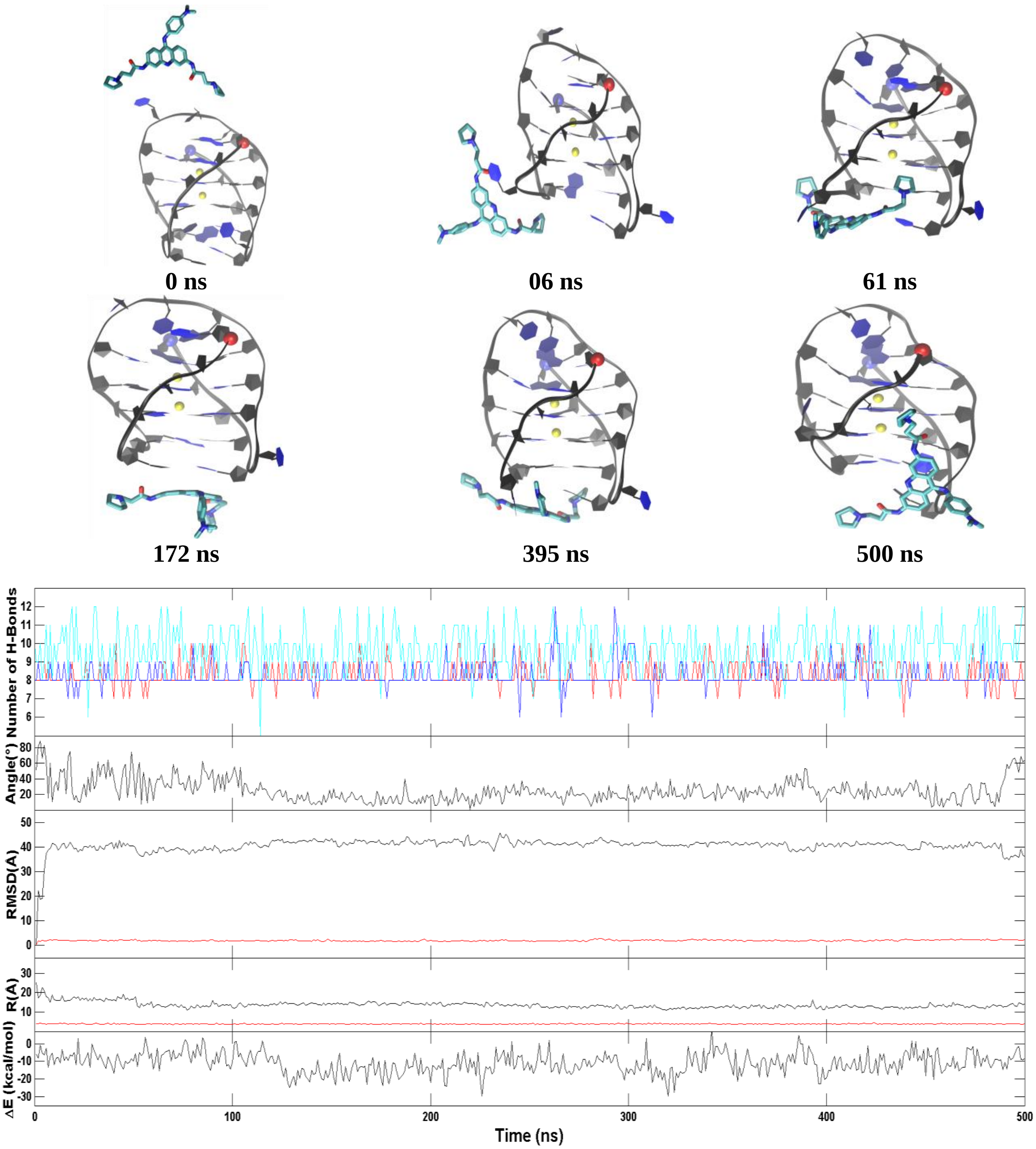

Figure S23. Another representative bottom stacking trajectory of the anti-parallel G-quadruplex. **Top:** Representative structures with time annotation. 5' and 3' are indicated by a red and blue ball, respectively. Residues 1, 2, 13, 14 are indicated in purple and residues 12, 24 are indicated in red and the K^+ ions are represented in yellow. **Bottom:** An order parameter plot depicting number of hydrogen bonds present in first G4 (green), second G4 (red) and third G4 (blue) tetrads of the DNA structure (Figure 2), the drug-base dihedral angle, receptor (red) and ligand (black) RMSD relative to the original crystal pose, center-to-center distance (R/black) and K^+-K^+ distance (R/red) and MM-GBSA binding energy (ΔE) (cf. methods section for definition).

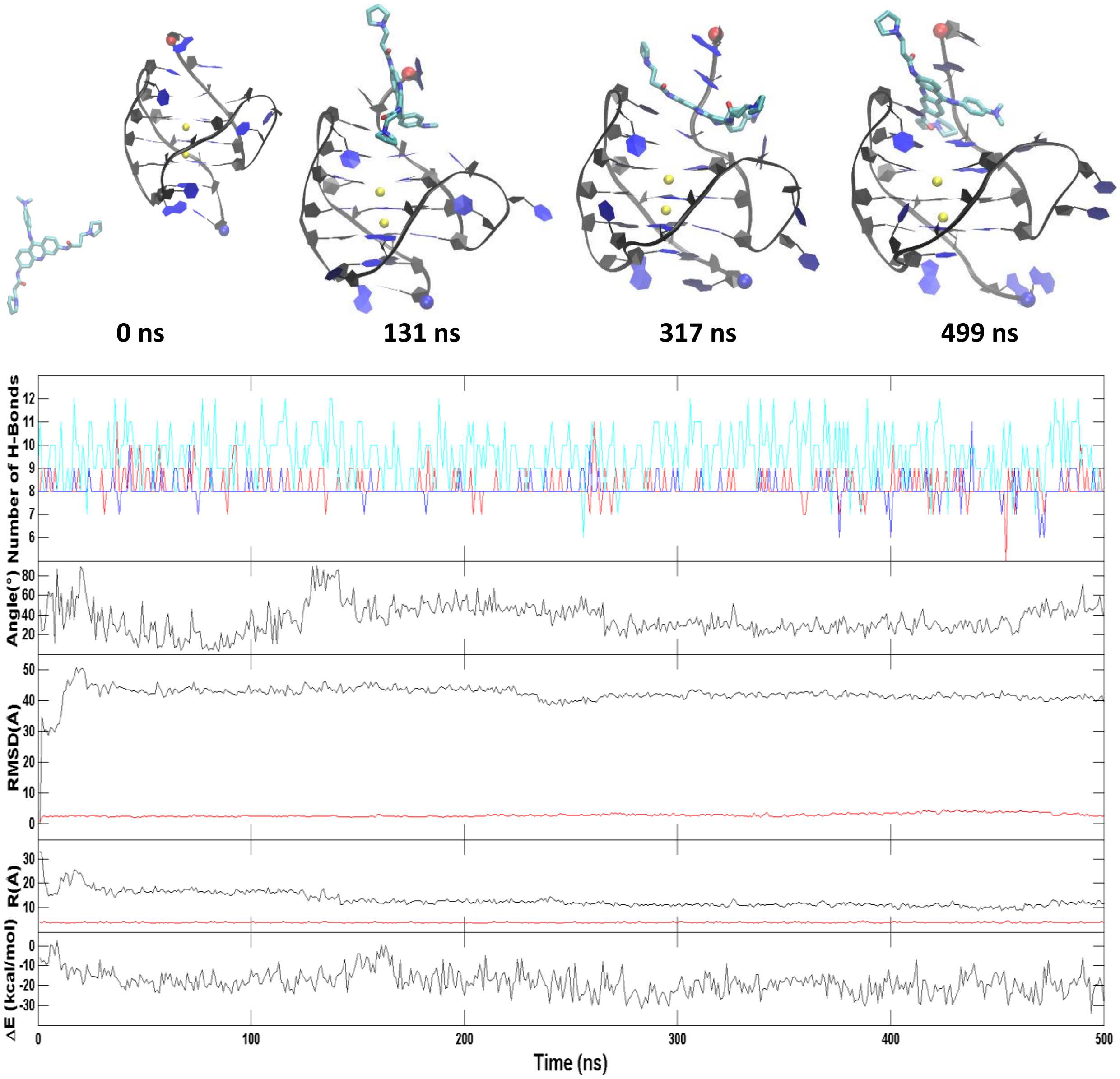

Figure S24. Another representative top binding trajectory of the hybrid G-quadruplex. **Top:** Representative structures with time annotation. 5' and 3' are indicated by a red and blue ball, respectively. Residues 1, 2, 13, 14 are indicated in purple and residues 12, 24 are indicated in red and the K^+ ions are represented in yellow. **Bottom:** An order parameter plot depicting number of hydrogen bonds present in first G4 (green), second G4 (red) and third G4 (blue) tetrads of the DNA structure (Figure 2), the drug-base dihedral angle, receptor (red) and ligand (black) RMSD relative to the original crystal pose, center-to-center distance (R/black) and K^+-K^+ distance (R/red) and MM-GBSA binding energy (ΔE) (cf. methods section for definition).

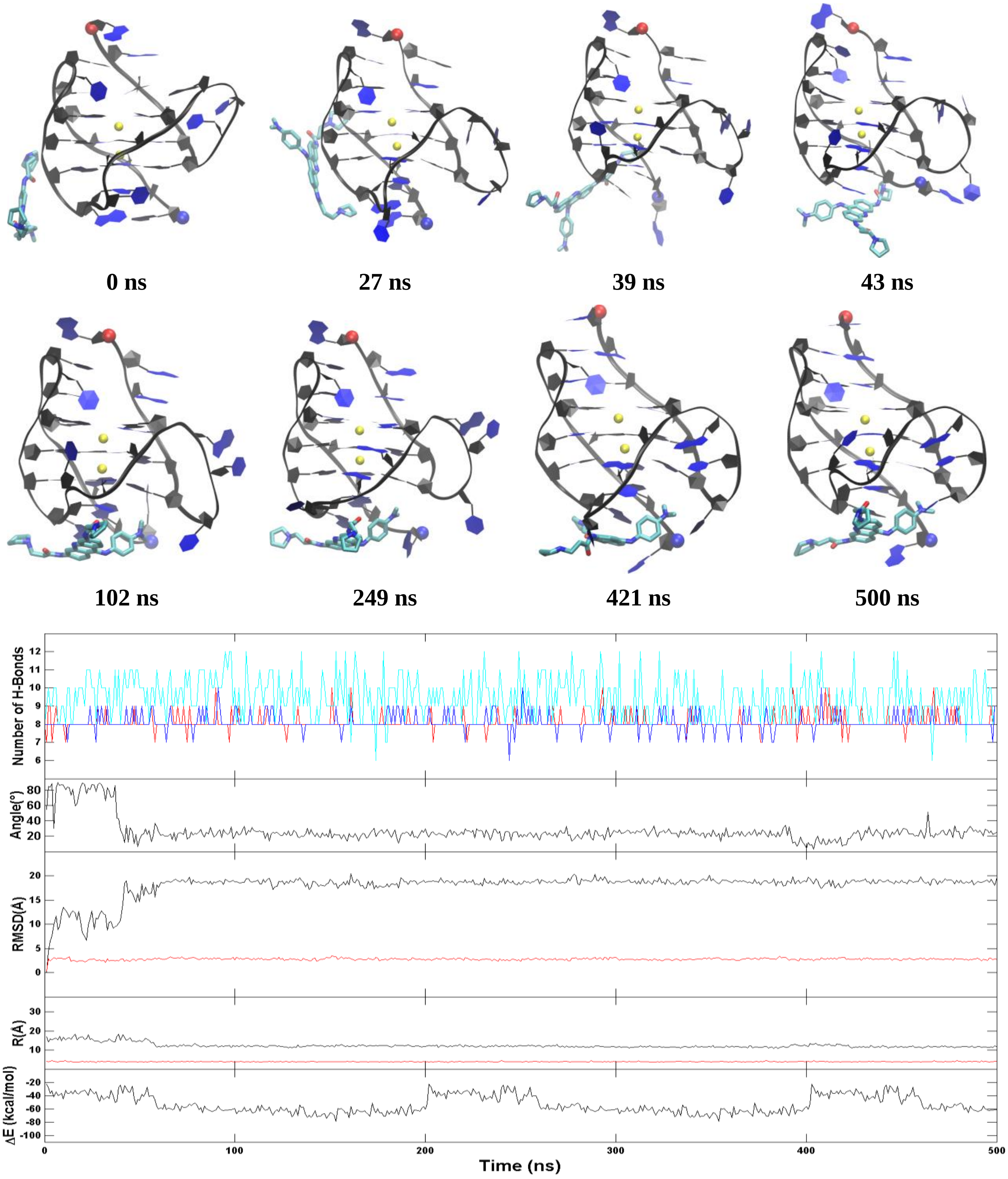

Figure S25. Another representative bottom binding trajectory of the hybrid G-quadruplex. **Top:** Representative structures with time annotation. 5' and 3' are indicated by a red and blue ball, respectively. Residues 1, 2, 13, 14 are indicated in purple and residues 12, 24 are indicated in red and the K^+ ions are represented in yellow. **Bottom:** An order parameter plot depicting number of hydrogen bonds present in first G4 (green), second G4 (red) and third G4 (blue) tetrads of the DNA structure (Figure 2), the drug-base dihedral angle, receptor (red) and ligand (black) RMSD relative to the original crystal pose, center-to-center distance (R/black) and K^+-K^+ distance (R/red) and MM-GBSA binding energy (ΔE) (cf. methods section for definition).

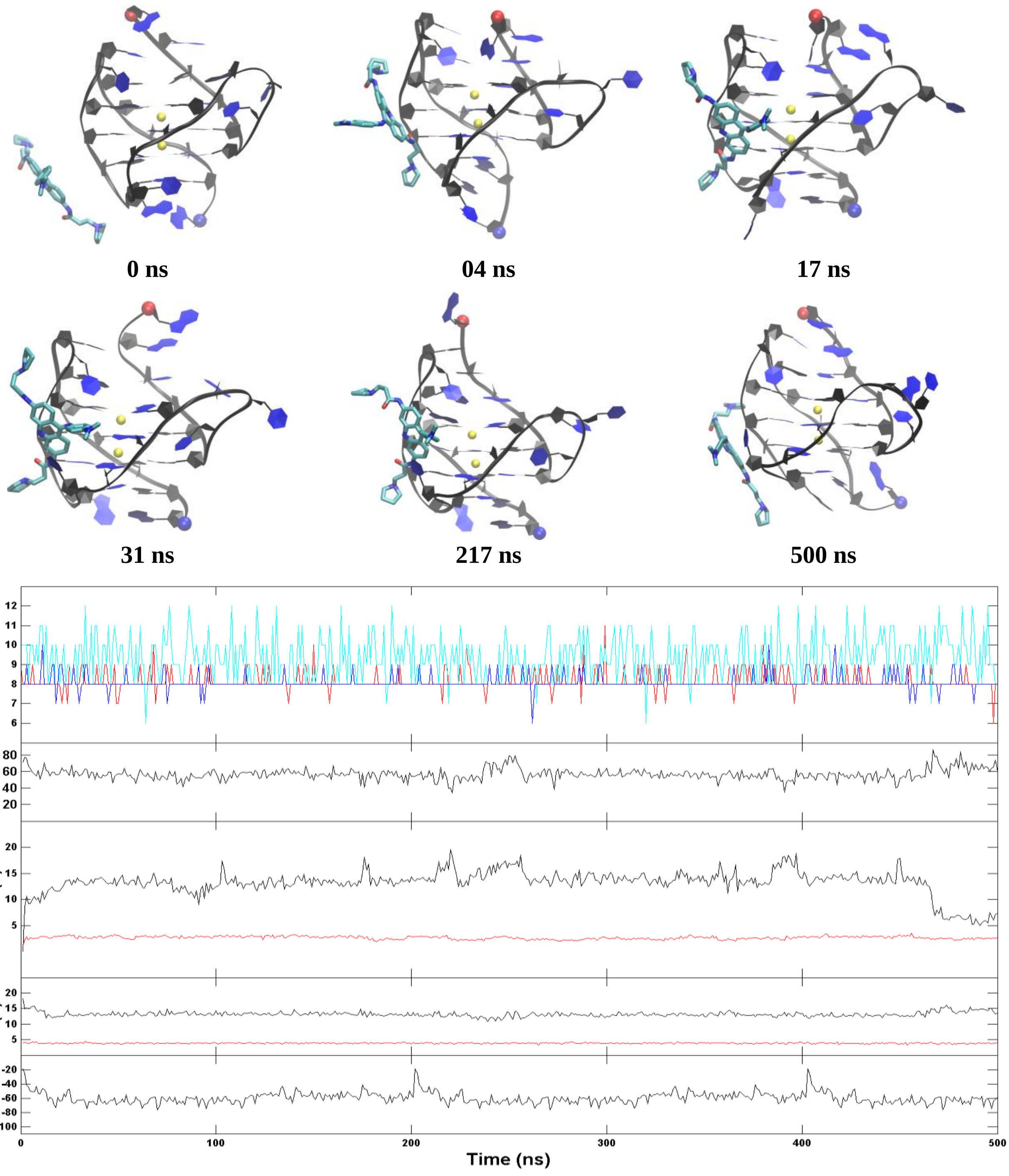

Figure S26. Another representative groove binding trajectory of the hybrid G-quadruplex. **Top:** Representative structures with time annotation. 5' and 3' are indicated by a red and blue ball, respectively. Residues 1, 2, 13, 14 are indicated in purple and residues 12, 24 are indicated in red and the K^+ ions are represented in yellow. **Bottom:** An order parameter plot depicting number of hydrogen bonds present in first G4 (green), second G4 (red) and third G4 (blue) tetrads of the DNA structure (Figure 2), the drug-base dihedral angle, receptor (red) and ligand (black) RMSD relative to the original crystal pose, center-to-center distance (R/black) and K^+-K^+ distance (R/red) and MM-GBSA binding energy (ΔE) (cf. methods section for definition).

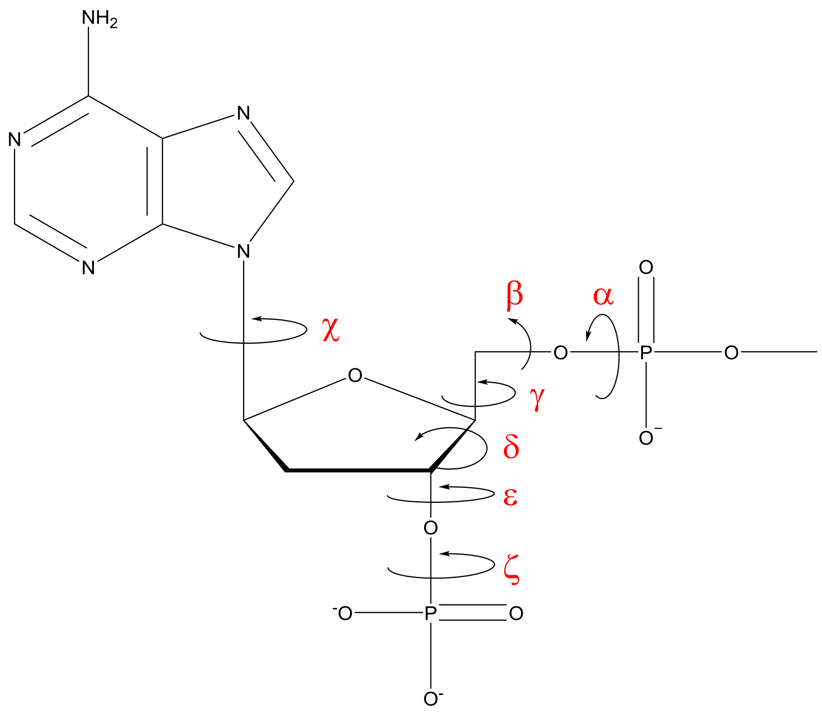

Figure S27. Backbone Torsion Angles of DNA.

System	Side View	Top View	Ligand Chemical Structure
Tel26_PtTripod (PDB ID: 5Z80)	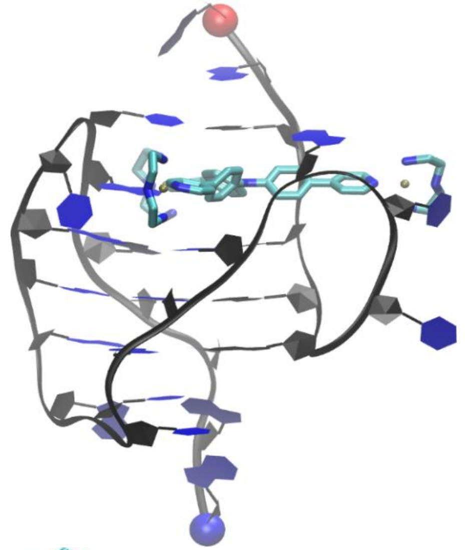	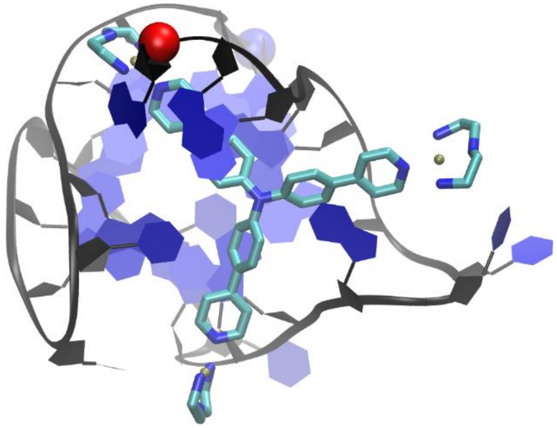	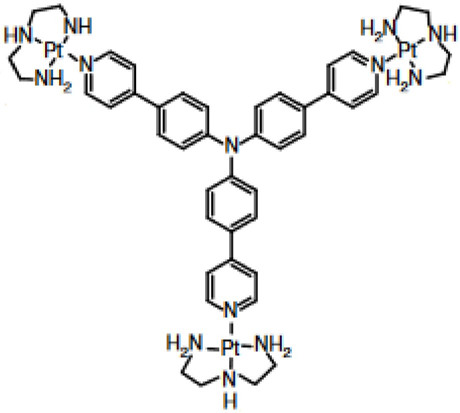
MD_Tel26_BRACO19	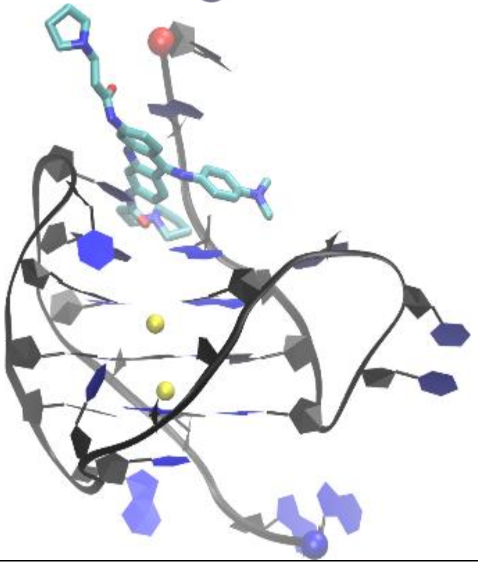	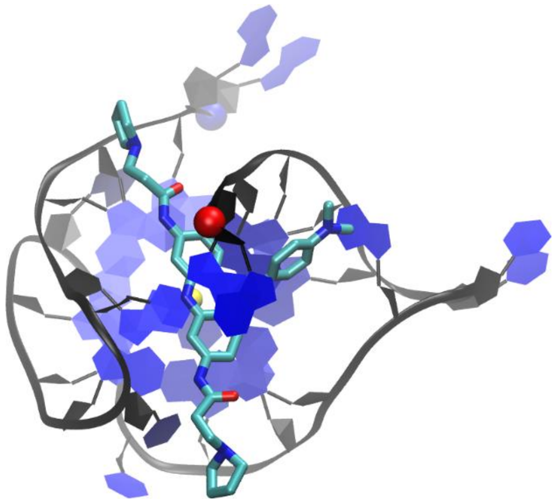	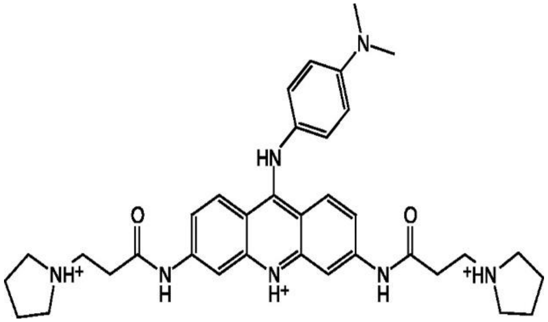

Figure S28. Comparison of Tel26 hybrid G-quadruplex-Pt Tripod Complex Crystal Structure (PDB ID: 5Z80) to Tel26 hybrid G-quadruplex-BRACO19 Complex. The 5' residues are represented by a red ball and the 3' residues in blue.

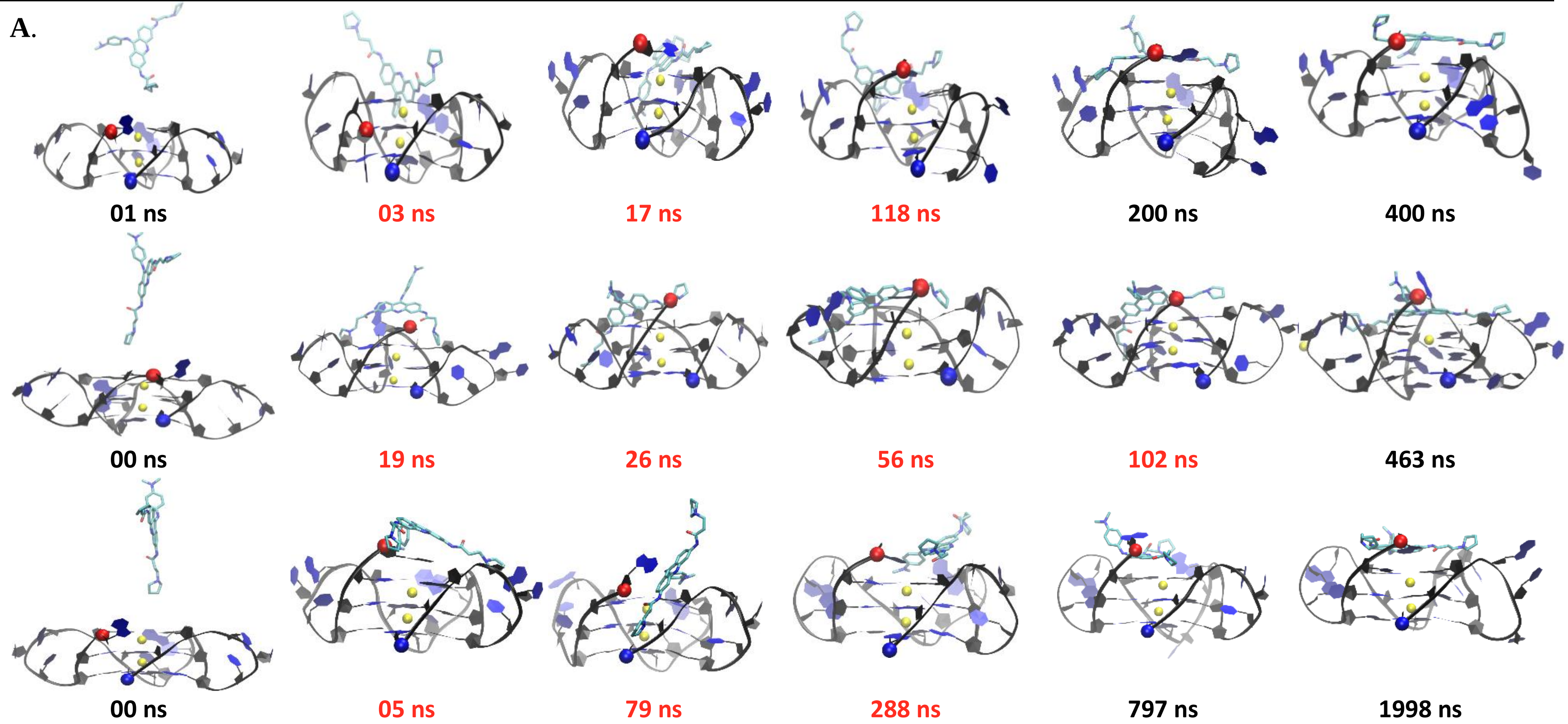
A.
01 ns
03 ns
17 ns
118 ns
200 ns
400 ns
00 ns
19 ns
26 ns
56 ns
102 ns
463 ns
00 ns
05 ns
79 ns
288 ns
797 ns
1998 ns

B.

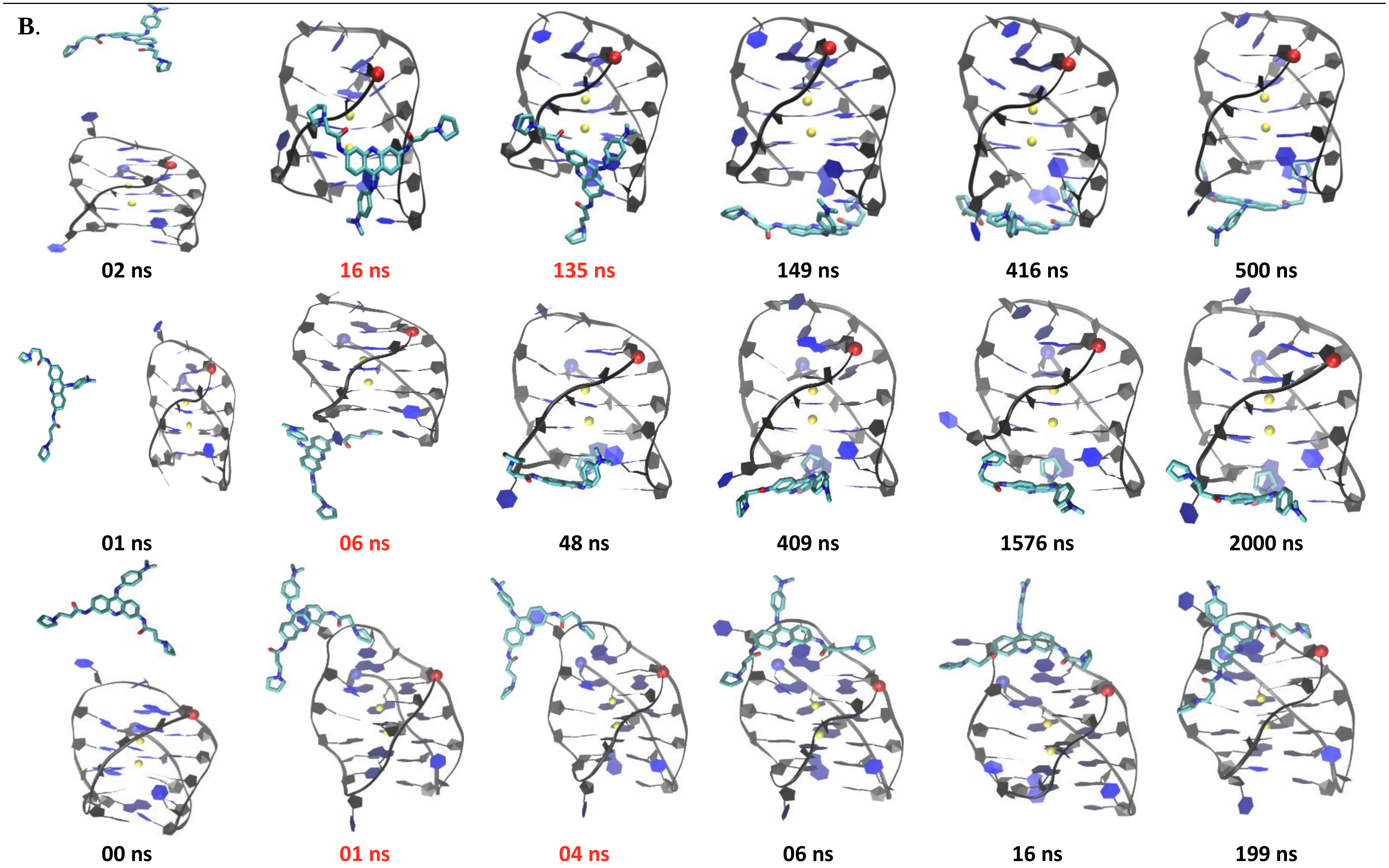
02 ns
16 ns
135 ns
149 ns
416 ns
500 ns
01 ns
06 ns
48 ns
409 ns
1576 ns
2000 ns
00 ns
01 ns
04 ns
06 ns
16 ns
199 ns

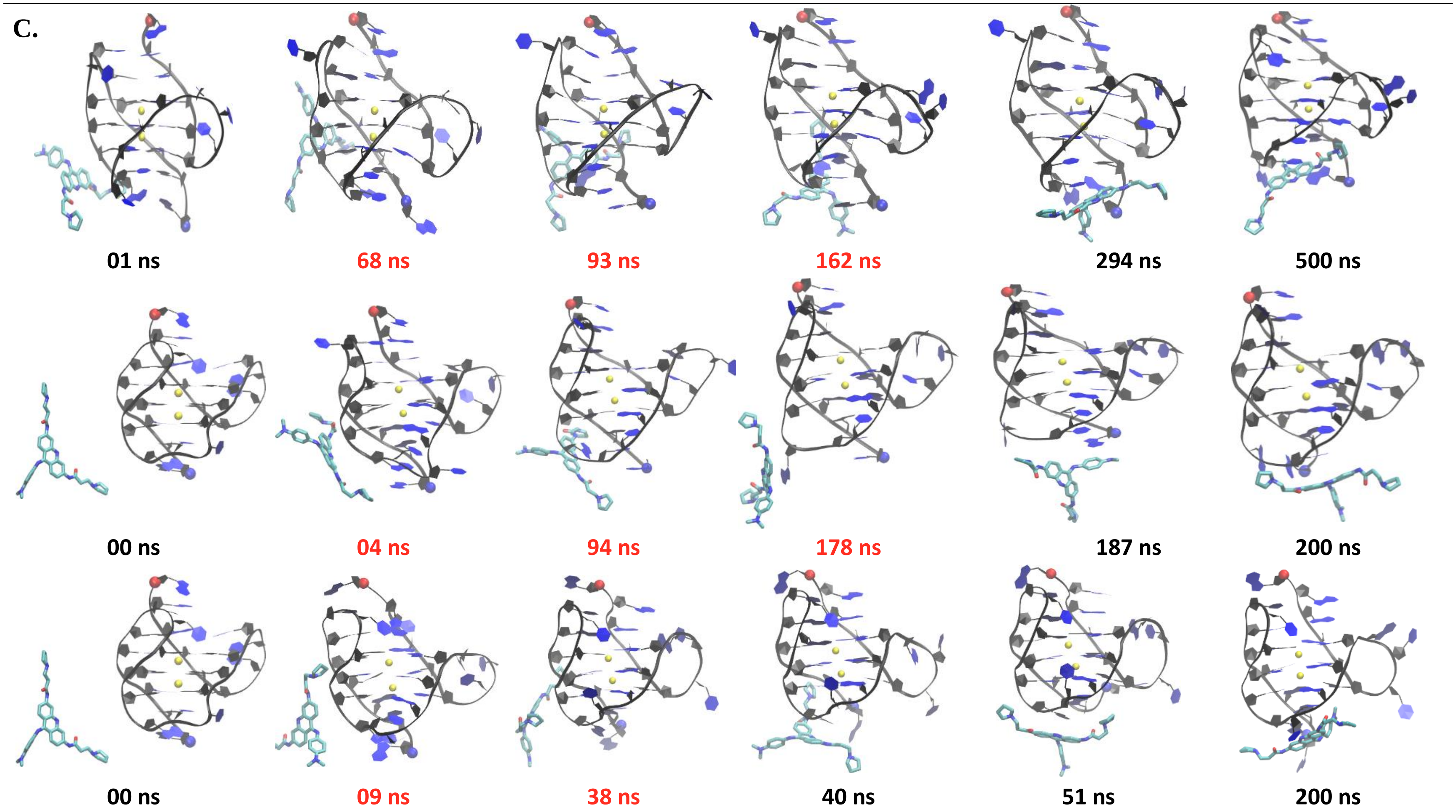

Figure S29. Representative snapshots of BRACO19 using groove binding as an intermediate state in each DNA G-quadruplex system: parallel (A), anti-parallel (B), and hybrid (C). The snapshots that BRACO19 is in a groove binding mode are in red. The 5' residues are represented by a red ball and the 3' residues in blue.

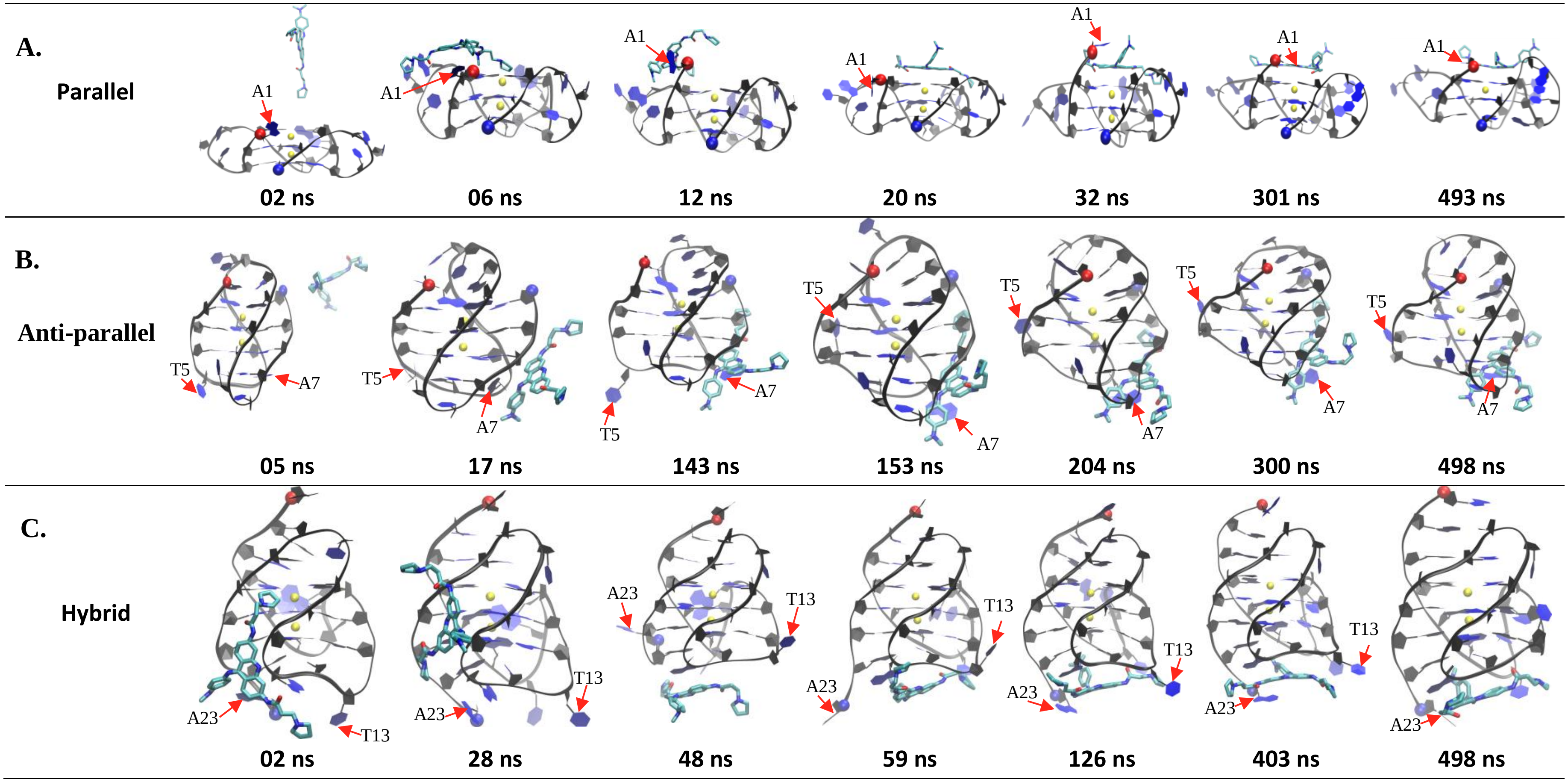

Figure S30. BRACO19 binding to the G-quadruplexes using an induced fit binding mechanism. The 5' residues are represented by a red ball and the 3' residues in blue.

Figure S31. Sampling plot of BRACO19 in complex with the (A) Parallel (B) Anti-Parallel (C) Hybrid and (D) DNA duplex systems.

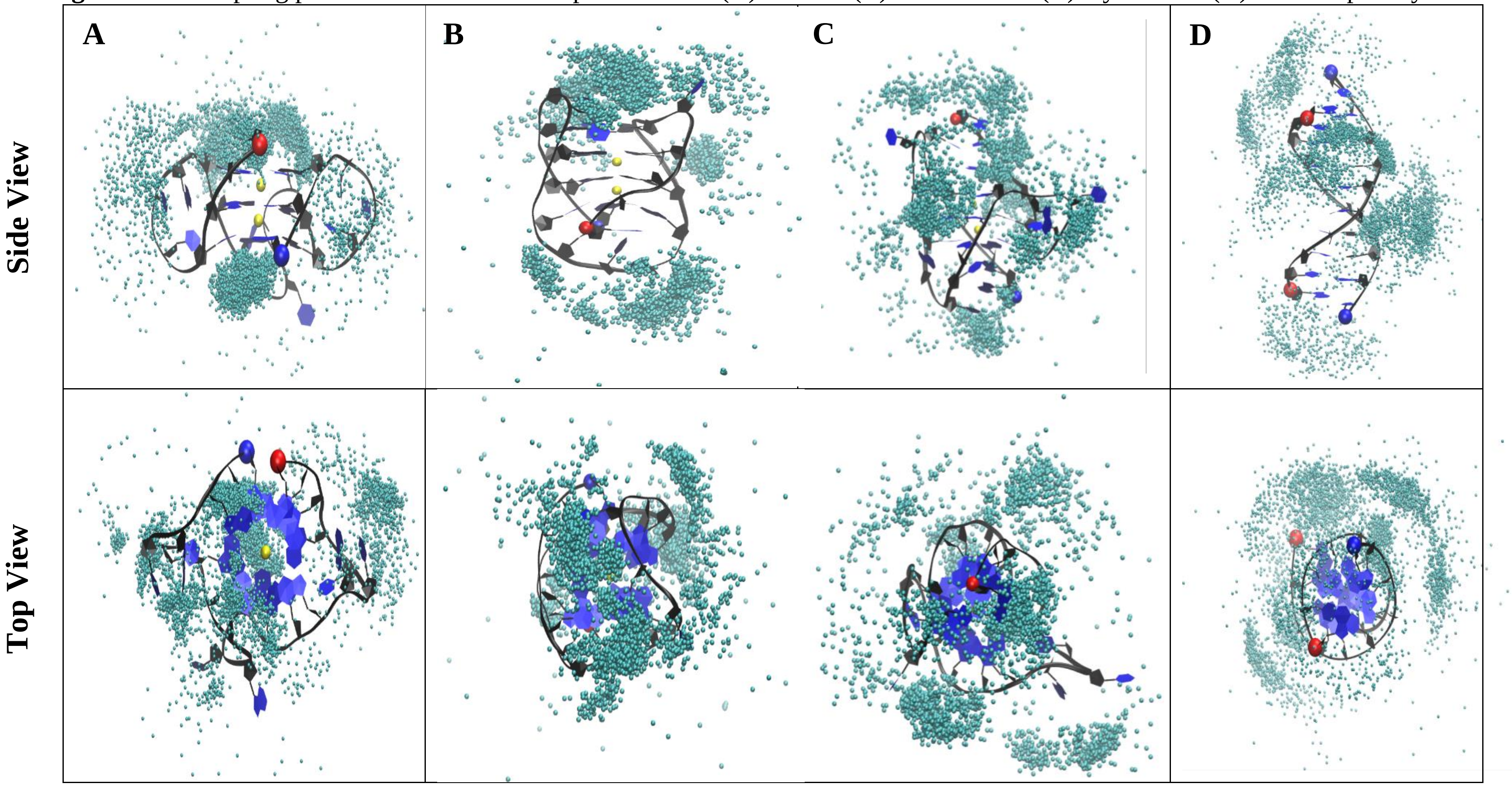

Supplement: Supplementary file 1 [file molecules-24-01010-s001.zip › supportingfiles/Supporting_finalrevision_unmarked.pdf]
